# Supplementary material for: COVID-19 among workers of a comprehensive cancer centre between first and second epidemic waves (2020): a seroprevalence study in Catalonia, Spain
Source: BMJ Open. 2022 Apr 21;12(4):e056637. doi: 10.1136/bmjopen-2021-056637 (PMC9023852; doi:10.1136/bmjopen-2021-056637)
Supplement: Supplementary data [file bmjopen-2021-056637supp001.pdf]

SUPPLEMENTARY MATERIAL

Accompanying the manuscript:

COVID-19 among workers of a Comprehensive Cancer Center between first and second epidemic waves (2020): a seroprevalence study in Catalonia, Spain.

Contents:

- Epidemiologic and behaviour questionnaire – ICO-Sero-COVID Study
- Supplementary Table 1. Demographic characteristics of on-site workers (always/ocassionally) and teleworkers
- Supplementary Table 2. Clinical characteristics associated with SARS-CoV-2 positive serology among those who report rRT-PCR previous to study serology (n=469).

Epidemiologic and behaviour questionnaire – ICO-Sero-COVID Study

I give my consent to participate in the study of seroprevalence of SARS-Cov-2 infection among ICO workers and related companies, which includes responding to an epidemiological survey with information on working conditions and obtaining a nasopharyngeal smear (to perform PCR test for virus detection) and/or to obtain blood sample by venipuncture (to perform serological tests for antibody determination and plasma cryopreservation at ICO biobank)

1 = Yes; 2 = No.

Thank you for participating in the COVID-19 seroprevalence survey among ICO workers. All information provided below will be treated confidentially, and all resulting results will be anonymized, with no individual data identifying participants.

A. *Socio-demographic data.*

1. **Name** string variable.
2. **Last name1** string variable.
3. **Last name2** string variable.
4. **CIP** string variable.
5. **DNI** numeric variable.
6. **Sex** numeric variable (1 = Woman; 2 = Man).
7. **E-mail** string variable.
8. **ICO center or external company** cathegoric variable (ICO-Gi, ICO-L'H, ICO-BDN, ICO-Tarragona-Terres Ebre, Arcasa, IDIBELL, ISS, Security, IDI, Pregecsa, Veolia).
9. **Professional category** numeric variable (1 = Nurse; 2 = Fac. Specialist (medicine, pharmacy, physics, psychologist); 3 = Higher Technician (Research, Predoc, Postdoc ...); 4 = MIR, FIR, PIR; 5 = Higher Technician; 6 = Porter; 7 = Administrative; 8 = Maintenance/Security; 9 = Cleaning; 10 = Restoration; 11 = Other (specify: string variable __________________).
10. **Work shift** numeric variable (1 = Morning; 2 = Afternoon; 3 = Night; 4 = Other).
11. **Did you telework for at least more than one day during the March to May 2020 period?** numeric variable (1 = Yes; 2 = No).
12. **How many days on average per week do you telework? |_ _|** numeric variable (1 to 7).

B. *Exposure and occupational safety measures data.*

13. **Have you worked in the "COVID area" during the period comprised between March and May 2020?** numeric variable (1 = No; 2 = Yes).
14. **Since the beginning of March 2020, have you had a suspected or confirmed clinical condition as COVID-19?** numeric variable (1 = Yes; 2 = No).
15. **Since the beginning of March 2020, have you had a nasopharyngeal smears sample?** numeric variable (1 = Yes; 2 = No).
16. **Do you belong to any of the groups considered to have an increased vulnerability to COVID -19?**
    a. **Cardiopathy / Hypertension** numeric variable (1 = Yes; 2 = No).
    b. **Respiratory disease** numeric variable (1 = Yes; 2 = No).
    c. **Hepatopathy** numeric variable (1 = Yes; 2 = No).
    d. **Nephropathy** numeric variable (1 = Yes; 2 = No).
    e. **Active Cancer** numeric variable (1 = Yes; 2 = No).
    f. **Immunosuppression** numeric variable (1 = Yes; 2 = No).
    g. **Diabetes mellitus** numeric variable (1 = Yes; 2 = No).
    h. **Pregnancy** numeric variable (1 = Yes; 2 = No).
17. **Have you had contact with patients with COVID-19 infection at ICO?** numeric variable (1 = Yes; 2 = No).
18. **Have you had contact with samples of COVID-19 patients at ICO?** numeric variable (1 = Yes; 2 = No).

19. **When you are in your workplace, do you wear a surgical mask?** numeric variable (1 = Yes; 2 = No).
20. **If you are in the COVID-19area, do you wear Personal Protective Equipment (PPE)?** numeric variable (1 = Yes; 2 = No, 3 = Not Applicable).
21. **Do you think that the Personal Protective Equipment (PPE) removal procedure is safe?** numeric variable (1 = Yes; 2 = No, 3 = I don't know, 4 = Not Applicable).
22. **Do you feel protected by the Personal Protective Equipment (PPE) used?** numeric variable (1 = Yes; 2 = No, 3 = I don't know, 4 = Not Applicable).
23. **Do you think that you may have been exposed to COVID-19 during personal relationships with your co-workers?** numeric variable (1 = Yes; 2 = No, 3 = I don't know).
24. **Do you think that the protection procedures implemented during this pandemic period will benefit you in your future professional development?** numeric variable (1 = Yes; 2 = No, 3 = I don't know).
25. **Do you think that the work activity carried out during this pandemic period has affected you or will affect you emotionally in the future?** numeric variable (1 = Yes; 2 = No, 3 = I don't know).

At work, do you wash your hands with soap or water or with a hydro-alcoholic solution...

26. **... before you start working?** numeric variable (1 = Yes; 2 = No).
27. **... every time you enter a new workspace?** numeric variable (1 = Yes; 2 = No).
28. **... before eating?** numeric variable (1 = Yes; 2 = No).
29. **... after handling money, mobile phone, other utensils ...?** numeric variable (1 = Yes; 2 = No).
30. **... less than 7 times during the working day?** numeric variable (1 = Yes; 2 = No).
31. **... at the end of the working day?** numeric variable (1 = Yes; 2 = No).

32. **When you eat, do you maintain a distance ≥ 2m from your colleagues?** numeric variable (1 = Yes; 2 = No, 3 = Not Applicable).
33. **Did your collegues cover thir face with their elbows when they sneeze / cough?** numeric variable (1 = Yes; 2 = No).

C. COVID-19 exposure outside working environament (home and social activities).

Outside working environment, do you wash your hands (with soap and water or hydro-alcoholic solution)...

34. **... when you get home?** numeric variable (1 = Yes; 2 = No).
35. **... before eating?** numeric variable (1 = Yes; 2 = No).
36. **... after handling money, mobile phone, other utensils** numeric variable (1 = Yes; 2 = No).
37. **... after cleaning?** numeric variable (1 = Yes; 2 = No).
38. **... after blowing your nose, sneezing or coughing?** numeric variable (1 = Yes; 2 = No).
39. **Do you shower and change clothes when you get home (or did you go to work)?:** numeric variable (1 = Yes; 2 = No).
40. **Do you wear a mask when you go shopping?** numeric variable (1 = Yes; 2 = No)
41. **Do the people you live with cover their elbows if they sneeze / cough?** numeric variable (1 = Yes; 2 = No)
42. **Do you have a cohabitant who has passed COVID-19 (with symptoms, with or without confirmation by PCR, or PCR + without symptoms)?** numeric variable (1 = Yes; 2 = No).
43. **Do you use public transport to go to work?** numeric variable (1 = Yes; 2 = No)
    If "yes", continue with question 44; if "no , jump to question 46.
44. **Which type of public transport?** numeric variable (1 = bus, 2 = metro, 3 = train, 4 = taxi, 5 = bicycle (multiple answer allowed)).
45. **How many days a week do you use public transport? |_ _|** numeric variable (1 to 7).
46. **Do you use private transportation to get to work?** numeric variable (1 = Yes; 2 = No).
    If "yes", continue with question 47; if "no", jump to question 49.
47. **Which private transport?** numeric variable (1 = single use car, 2 = shared car, 3 = single use bike, 4 = shared bike, 5 = bike (multiple answer allowed)).
48. **How many days per week do you use private transport? |_ _|** numeric variable (1 to 7).

49. **Do you walk the street for more than 15 minutes?** numeric variable (1 = Yes; 2 = No).
    If "yes", continue with question 50; if "no", jump to question 52.
50. **How many days a week do you go for a walk?** |_ _| numeric variable (1 to 7).
51. **For how many minutes a day do you go for a walk as an average:** |_ _| minutes / day numeric variable.

D. COVID-19 tests performed

52. **Have you had a COVID-19 PCR test?** numeric variable (1 = Yes, only one; 2 = Yes, several; 3 = No).
    If "1", continue with question 53; if "2", jump to question 55; if "3", jump to question 59.
53. **COVID-19 PCR test performed on day:** | *dd* | *mm* | *yy*|.
54. **COVID-19 PCR test result** numeric variable (1 = Negative; 2 = Positive).
55. **How many COVID-19 PCR test have you had in total?** |_ _| numeric variable (1 to 10).
    Depending on the answer, open as many questions with the number of PCR made to ask the date and result in the same format (A1 and A2; B1 and B2; etc).
    A1. **COVID-19 PCR test performed on day:** | *dd* | *mm* | *yy*|.
    A2. **COVID-19 PCR test result** numeric variable (1 = Negative; 2 = Positive).
56. **When you had your first COVID-19 PCR test, did you present any of these signs or symptoms?** numeric variable (multiple answer allowed) (1 = Febricula (>37.3ºC); 2 = Fever (>38°C); 3 = Cough; 4 = Odynophagia (sorethroat); 5 = Headache; 6 = Arthromyalgia (generalized pain); 7 = Asthenia (intense fatigue); 8 = Dyspnoea (shortness of breath); 9 = Anosmia (loss of smell); 10= Nausea, vomiting; 11 = Diarrhea; 12 = Skin lesions; 13 = Myoclonus (involuntary movements); 14 = Pneumonia; 15 = Other (specify: string variable __________________).
57. **Have you had a COVID-19 rapid antibody test?** numeric variable (1 = Yes; 2 = No).
58. **COVID-19 rapid antibody test result** numeric variable (1 = Negative; 2 = Positive).

Finally, we would like to complete the information provided with information about your lifestyle.

E. Lifestyle

59. **Do you drink any alcoholic beverage at least once a week?** numeric variable (1 = Yes; 2 = No).
    If "yes", continue with question 60; if "no", jump to question 64.
60. **How many glasses of wine do you drink every week?** |_ _| numeric variable
61. **How many beers do you drink every week?** |_ _| numeric variable
62. **How many glasses of cognac, gin or other spirits do you drink every week?** |_ _| numeric variable
63. **Has your alcohol consumption changed during the pandemic compared to your consumption previously?** numeric variable (1 = No, it is similar; 2 = Yes, it has increased; 3 = Yes, it has decreased).
64. **Regarding tobacco use:** numeric variable (1 = I have never smoked; 2 = I am a former smoker; 3 = I am a corrent smoker).
    If "1" or "2", jump to question 67; If "3", continue to question 65.
65. **How many roll-ypur-own cigarrettes do you smoke every day?** |_ _| numeric variable
66. **Has your tobacco consumption changed during the pandemic compared to your consumption previously?** numeric variable (1 = No, it is similar; 2 = Yes, it has increased; 3 = Yes, it has decreased).

F. End of the survey

Thank you very much for your participation. As mentioned before, all information from this survey and the tests performed is confidential and will be anonymized.
If you would like to leave us any further comments regarding the pandemic at the ICO Centers, please do so below:

67. **Commentaries**. *Open answer, leave space for about 5 lines of text.*

Supplementary Table 1. Demographic characteristics of on-site workers (always/ocassionally) and teleworkers

	**Teleworking**		
	**Never/ocassionally (n=981)**<br>n (%)	**Always (n=230)**<br>n (%)	***p-value***
**Sex**			
Male	240 (25)	47 (20)	
Female	736 (75)	183 (80)	*0,183*
**Age [median, (min-max)]**	43 (19-68.5)	44.9 (19-71.6)	*0,015*
<35y	271 (28)	38 (17)	
35-49y	429 (44)	122 (53)	
>49y	281 (29)	70 (30)	*0,002*
**ICO Center**			
ICO L'Hospitalet	684 (70)	184 (80)	
ICO Girona	182 (19)	17 (7)	
ICO Badalona	103 (11)	29 (13)	
ICO Tarragona / Terres de l'Ebre	12 (1)	0 (0)	*<0.0001*
**Health care workers**			
Yes	567 (59)	72 (32)	
No	402 (41)	152 (68)	*<0.0001*
Middle and superior technicians	*187 (19)*	*92 (41)*	
Porters	*17 (2)*	*3 (1)*	
Administrative staff	*90 (9)*	*35 (16)*	
Maintenance or security stafft	*29 (3)*	*2 (1)*	
Cleaning staff	*46 (5)*	*15 (7)*	
Restoration staff	*16 (2)*	*2 (1)*	
Others	*17 (2)*	*3 (1)*	*<0.0001*
**Any Comorbidity**	142 (15)	38 (17)	*0,4*
**Smoking history**			
Never	511 (54)	126 (56)	
Ever	438 (46)	98 (44)	*0,5*
Past	277 (29)	66 (29)	
Current	161 (17)	32 (14)	*0,6*
**Cohabiting**	889 (91)	209 (92)	*0,8*
**Cohabiting with covid-19**	115 (14)	27 (14)	*0,9*
**Reported rRT-PCR previous to serology**	422 (84)	42 (75)	*0,1*
**Positive of previous rRT-PCR**	62 (15)	10 (24)	*0,1*

Supplementary Table 2. Clinical characteristics associated with SARS-CoV-2 positive serology among those who report rRT-PCR previous to study serology (n=469).

	Total participants n (%)	SARS-CoV-2 seroprevalence n (%)	Prevalence (95%CI)	p-value[1]	Adjusted PR (95% CI)[2]
**Reported rRT-PCR previous to serology**	469 (38.0)	86 (78.2)	18.34 (15.08-22.11)		
**Result of previous rRT-PCR**					
Negative	397 (84.6)	27 (31.0)	6.80 (4.70-9.74)		REF
Positive	72 (15.4)	59 (68.6)	81.94 (71.31-89.23)	<0.001	12.15 (7.54-19.57)
**Number of symptoms***(mean, standard deviation)*	1.65 (2.10)	3.08 (2.61)		<0.001	
None	217 (46.3)	21 (24.0)	9.68 (6.39-14.4)		REF
One	61 (13)	7 (8.1)	11.48 (5.56-22.21)		1.13 (0.48-2.67)
2-3	109 (23.2)	22 (25.6)	20.18 (13.66-28.78)		2.03 (1.10-3.73)
≥4	81 (17.3)	35 (40.7)	43.21 (32.87-54.18)	<0.001	4.33 (2.48-7.59)
p-trend (among exposed)					*<0.001*
**Reporting COVID-19 compatible symptoms when rRT-PCR was performed**					
No	217 (46.3)	21 (24.0)	9.68 (6.39-14.4)		REF
Yes	251 (53.5)	64 (74.4)	25.5 (20.48-31.27)	<0.001	2.49 (1.51-4.10)
**COVID-19 symptoms**					
Headache	126 (26.9)	36 (41.9)	28.57 (21.35-37.08)	<0.001	1.87 (1.20-2.93)
Cough	119 (25.4)	37 (43.0)	31.09 (23.42-39.97)	<0.001	2.25 (1.44-3.52)
Asthenia	110 (23.5)	36 (41.9)	32.73 (24.6-42.04)	<0.001	2.38 (1.53-3.72)
Arthromyalgia	80 (17.1)	57 (66.0)	36.25 (26.47-47.31)	<0.001	2.32 (1.47-3.67)
Low-grade fever (37.3ºC-38ºC)	73 (15.6)	26 (30.2)	35.62 (25.5-47.21)	<0.001	2.71 (1.67-4.39)
Odynophagia	64 (13.6)	14 (16.3)	21.88 (13.39-33.65)	0.40	1.18 (0.65-2.13)
Diarrhoea	58 (12.4)	16 (18.6)	27.59 (17.62-40.43)	0.05	1.47 (0.83-2.60)
Anosmia	42 (9)	33 (38.4)	78.57 (63.65-88.48)	<0.001	6.09 (3.86-9.60)
Dyspnoea	40 (8.5)	11 (12.8)	27.50 (15.91-43.2)	0.12	1.56 (0.81-3.00)
Fever (>38ºC)	28 (6)	15 (17.4)	53.57 (35.4-70.84)	<0.001	3.06 (1.71-5.46)
Nausea / vomiting	17 (3.6)	6 (7)	35.29 (16.75-59.66)	0.07	1.86 (0.80-4.36)
Skin lesions	8 (1.7)	1 (1.2)	12.50 (1.72-53.86)	0.66	0.74 (0.10-5.38)
Pneumonia	3 (0.6)	2 (2.3)	66.67 (15.27-95.69)	0.03	2.99 (0.71-12.63)
Myoclonus	2 (0.4)	0		0.50	

Numbers do not always sum up the total due to some missing values (none of the categories present more than 5% of missing values). PR: Prevalence Ratio, CI: Confidence Interval. [1] Chi-squared test for categorical variables (Fisher's exact test corrected for continuity) and median test for continuous variables. [2] Adjusted for sex, age (continuous), ICO center, care staff, telework and cohabitants.

BMJ Publishing Group Limited (BMJ) disclaims all liability and responsibility arising from any reliance
placed on this supplemental material which has been supplied by the author(s)
