# Supplementary material for: COVID-19 among workers of a comprehensive cancer centre between first and second epidemic waves (2020): a seroprevalence study in Catalonia, Spain
Source: BMJ Open. 2022 Apr 21;12(4):e056637. doi: 10.1136/bmjopen-2021-056637 (PMC9023852; doi:10.1136/bmjopen-2021-056637)
Supplement: Author's manuscript [file bmjopen-2021-056637.draft_revisions.pdf]

BMJ Open is committed to open peer review. As part of this commitment we make the peer review history of every article we publish publicly available.

When an article is published we post the peer reviewers' comments and the authors' responses online. We also post the versions of the paper that were used during peer review. These are the versions that the peer review comments apply to.

The versions of the paper that follow are the versions that were submitted during the peer review process. They are not the versions of record or the final published versions. They should not be cited or distributed as the published version of this manuscript.

BMJ Open is an open access journal and the full, final, typeset and author-corrected version of record of the manuscript is available on our site with no access controls, subscription charges or pay-per-view fees (http://bmjopen.bmj.com).

If you have any questions on BMJ Open's open peer review process please email info.bmjopen@bmj.com

BMJ Open

COVID-19 seroprevalence among workers of a Comprehensive Cancer Center in Catalonia, Spain.

Journal:	*BMJ Open*
Manuscript ID	bmjopen-2021-056637
Article Type:	Original research
Date Submitted by the Author:	27-Aug-2021
Complete List of Authors:	Peremiquel-Trillas , Paul; Catalan Institute of Oncology, Tobacco Control Unit<br>Saura-Lazaro, Anna; Catalan Institute of Oncology, Tobacco Control Unit<br>Benavente Moreno, Yolanda<br>Casabonne, Delphinne; Catalan Institute of Oncology<br>Loureiro, Eva; Catalan Institute of Oncology<br>Cabrera, Sandra; Catalan Institute of Oncology<br>Duran, Angela; Catalan Institute of Oncology<br>Garrote, Lidia; Catalan Institute of Oncology<br>Brao, Immaculada; Catalan Institute of Oncology<br>Trelis, Jordi; Catalan Institute of Oncology<br>Galán, Maica; Catalan Institute of Oncology<br>Soler, Francesc; Catalan Institute of Oncology<br>Julia, Joaquim; Catalan Institute of Oncology<br>Cortasa, Dolça; Catalan Institute of Oncology<br>Domínguez, Maria Ángeles; Bellvitge University Hospital<br>Albasanz-Puig, Adaia; Catalan Institute of Oncology<br>Gudiol, Carlota; Catalan Institute of Oncology<br>Ramírez, Dolors; Catalan Institute of Oncology<br>Muniesa, Joan; Catalan Institute of Oncology<br>Rivas, Juan; Catalan Institute of Oncology<br>Muñoz-Montplet, Carles; Catalan Institute of Oncology<br>Sedano, Ana; Catalan Institute of Oncology<br>Plans, Àngel; Catalan Institute of Oncology<br>Calvo-Cerrada, Beatriz; Catalan Institute of Oncology<br>Calle, Candela; Catalan Institute of Oncology<br>Clopés, Ana; Catalan Institute of Oncology<br>Carnicer-Pont, Dolors; Hospital Duran i Reynals,<br>Alemany, L.; Institut Catala d' Oncologia,<br>Fernandez, Esteve; Catalan Institute of Oncology, Tobacco Control Unit
Keywords:	COVID-19, Adult oncology < ONCOLOGY, Epidemiology < INFECTIOUS DISEASES, Epidemiology < ONCOLOGY

SCHOLARONE™
Manuscripts

For peer review only - http://bmjopen.bmj.com/site/about/guidelines.xhtml

I, the Submitting Author has the right to grant and does grant on behalf of all authors of the Work (as defined in the below author licence), an exclusive licence and/or a non-exclusive licence for contributions from authors who are: i) UK Crown employees; ii) where BMJ has agreed a CC-BY licence shall apply, and/or iii) in accordance with the terms applicable for US Federal Government officers or employees acting as part of their official duties; on a worldwide, perpetual, irrevocable, royalty-free basis to BMJ Publishing Group Ltd ("BMJ") its licensees and where the relevant Journal is co-owned by BMJ to the co-owners of the Journal, to publish the Work in this journal and any other BMJ products and to exploit all rights, as set out in our licence.

The Submitting Author accepts and understands that any supply made under these terms is made by BMJ to the Submitting Author unless you are acting as an employee on behalf of your employer or a postgraduate student of an affiliated institution which is paying any applicable article publishing charge ("APC") for Open Access articles. Where the Submitting Author wishes to make the Work available on an Open Access basis (and intends to pay the relevant APC), the terms of reuse of such Open Access shall be governed by a Creative Commons licence – details of these licences and which Creative Commons licence will apply to this Work are set out in our licence referred to above.

Other than as permitted in any relevant BMJ Author's Self Archiving Policies, I confirm this Work has not been accepted for publication elsewhere, is not being considered for publication elsewhere and does not duplicate material already published. I confirm all authors consent to publication of this Work and authorise the granting of this licence.

For peer review only

COVID-19 seroprevalence among workers of a Comprehensive Cancer Center in Catalonia, Spain.

Paula Peremiquel-Trillas, Anna Saura-Lázaro*, Yolanda Benavente-Moreno, Delphine Casabonne, Eva Loureiro, Sandra Cabrera, Angela Durán, Lidia Garrote, Inmaculada Brao, Jordi Trelis, Maica Galán, Francesc Soler, Joaquim Julià, Dolça Cortasa, M. Angeles Domínguez, Adaia Albasanz-Puig, Carlota Gudiol, Dolors Ramírez, Joan Muniesa, Juan Pedro Rivas, Carles Muñoz-Montplet, Ana Sedano, Àngel Plans, Beatriz Calvo-Cerrada, Candela Calle, Ana Clopés, Dolors Carnicer-Pont†, Laia Alemany†, Esteve Fernández†*

*Contributed equally

†Joint senior authors

Authors' affiliations

PPT Cancer Epidemiology Research Programme, Cancer Epidemiology and Prevention Department, Institut Català d'Oncologia (ICO), L'Hospitalet de Llobregat, Spain; Epidemiology and Public Health Programme, Institut d'Investigació Biomèdica de Bellvitge (IDIBELL) , L'Hospitalet de Llobregat, Spain; CIBER in epidemiology and public health (CIBERESP), Madrid, Spain; School of Medicine and Clinical Sciences, Universitat de Barcelona, Barcelona, Spain. @PeremiquelPaula. ORCID: 0000-0002-8636-1725

ASL Cancer Epidemiology Research Programme, Cancer Epidemiology and Prevention Department, Institut Català d'Oncologia (ICO), L'Hospitalet de Llobregat, Spain; Epidemiology and Public Health Programme, Institut d'Investigació Biomèdica de Bellvitge (IDIBELL) , L'Hospitalet de Llobregat, Spain. 0000-0001-9742-2725

YBM Cancer Epidemiology Research Programme, Cancer Epidemiology and Prevention Department, Institut Català d'Oncologia (ICO) , L'Hospitalet de Llobregat, Spain; Epidemiology and Public Health Programme, Institut d'Investigació Biomèdica de Bellvitge (IDIBELL) , L'Hospitalet de Llobregat, Spain; CIBER of Epidemiology and Public Health (CIBERESP), Madrid, Spain. ORCID: 0000-0003-1422-4614

DC Cancer Epidemiology Research Programme, Cancer Epidemiology and Prevention Department, Institut Català d'Oncologia (ICO) , L'Hospitalet de Llobregat, Spain; Epidemiology and Public Health Programme, Institut d'Investigació Biomèdica de Bellvitge (IDIBELL) , L'Hospitalet de Llobregat, Spain; CIBER of Epidemiology and Public Health (CIBERESP), Madrid, Spain. ORCID: 0000-0002-7874-3707

EL Computer Science Services, Technology & Physics, Institut Català d'Oncologia (ICO) , L'Hospitalet de Llobregat, Spain; Computational Science and Artificial Intelligence, Faculty of Computer Science of Coruña, University of Coruña (UDC), Coruña, Spain.

SC Research Nursing Department, Institut Català d'Oncologia (ICO), L'Hospitalet de Llobregat, Spain. @scabrerajaime. ORCID: 0000-0003-3013-8812

AD Nursing Department, Institut Català d'Oncologia (ICO), L'Hospitalet de Llobregat, Spain.

LG Nursing Department, Institut Català d'Oncologia – Badalona (ICO), Badalona, Spain.

IB Nursing Department, Institut Català d'Oncologia – Girona (ICO), Girona, Spain

JT Palliative Care Department and Medical Director, Institut Català d'Oncologia (ICO), L'Hospitalet de Llobregat, Spain. @JordiTrelis.

MG Esofagogastric Tumours Functional Unit and Medical Director, Institut Català d'Oncologia (ICO), L'Hospitalet de Llobregat, Spain.

FS Pharmacy Service and Medical Director, Institut Català d'Oncologia – Girona (ICO), Girona, Spain

JJT Palliative Care Department and Medical Director. Institut Català d'Oncologia (ICO) Badalona, Badalona, Spain. @drjjulia. ORCI: 0000-0002-1462-3167

DC Medical Director, Institut Català d'Oncologia -Tarragona/Terres de l'Ebre, Tarragona (ICO).

MADL Microbiology Department. Hospital Universitari de Bellvitge; Infectious Diseases Programme, Institut d'Investigació Biomèdica de Bellvitge (IDIBELL); Department of Pathology Experimental Therapeutics, Universitat de Barcelona, Barcelona, Spain.

AAP Infectious Disease Department, Hospital Universitari de Bellvitge, Institut Català d'Oncologia (ICO), L'Hospitalet de Llobregat, Spain. ORCID: 0000-0001-9852-5574

CG Infectious Disease Department, Hospital Universitari de Bellvitge, Institut Català d'Oncologia (ICO), L'Hospitalet de Llobregat, Spain. @Carlotasway3. ORCID: 0000-0003-3095-4422

For peer review only - http://bmjopen.bmj.com/site/about/guidelines.xhtml

DR Preventive Medicine Unit, Institut Català d'Oncologia (ICO), L'Hospitalet de Llobregat, Spain.

JM Computer Science Services, Technology & Physics Department, Institut Català d'Oncologia (ICO), L'Hospitalet de Llobregat, Spain.

JPR Computer Science Services, Technology & Physics Department, Institut Català d'Oncologia (ICO), L'Hospitalet de Llobregat, Spain.

CMM Technology and Physics Department, Institut Català d'Oncologia (ICO), L'Hospitalet de Llobregat, Spain; Medical Physics and Radiation Protection Department, Institut Català d'Oncologia (ICO) – Girona, Girona, Spain; Department of Medical Sciences, Universitat de Girona, Girona, Spain. @CMunozMontplet. ORCID: 0000-0002-7324-8889

AS Human Resources Department, Institut Català d'Oncologia (ICO), L'Hospitalet de Llobregat, Spain.

AC Scientific Direction, Institut Català d'Oncologia (ICO), L'Hospitalet de Llobregat, Spain. ORCID: 0000-0002-1857-362X

BCC Occupational Health Unit, Institut Català d'Oncologia (ICO), L'Hospitalet de Llobregat, Spain.

CC General Direction, Institut Català d'Oncologia (ICO), L'Hospitalet de Llobregat, Spain. @CandelaCalle.

AP Occupational Health Unit, Institut Català d'Oncologia (ICO), L'Hospitalet de Llobregat, Spain. ORCID: 0000-0003-3199-0361

DCP Cancer Prevention and Control Programme, Cancer Epidemiology and Prevention Department, Catalan Institute of Oncology (ICO) , L'Hospitalet de Llobregat, Spain; Epidemiology and Public Health Programme, Institut d'Investigació Biomèdica de Bellvitge (IDIBELL), L'Hospitalet de Llobregat, Spain. ORCID: 0000-0002-3475-8704

LA Cancer Epidemiology Research Programme, Cancer Epidemiology and Prevention Department, Institut Català d'Oncologia (ICO) , L'Hospitalet de Llobregat, Spain; Epidemiology and Public Health Programme, Institut d'Investigació Biomèdica de Bellvitge (IDIBELL), L'Hospitalet de Llobregat, Spain; CIBER of Epidemiology and Public Health (CIBERESP), Madrid, Spain. ORCID: 0000-0003-0945-6015

EF Cancer Prevention and Control Programme, Cancer Epidemiology and Prevention Department, Institut Català d'Oncologia (ICO) , L'Hospitalet de Llobregat, Spain; Epidemiology and Public Health Programme, Institut d'Investigació Biomèdica de Bellvitge (IDIBELL), L'Hospitalet de Llobregat, Spain; School of Medicine and Clinical Sciences, Universitat de Barcelona, Barcelona, Spain; CIBER of Respiratory Diseases (CIBERES). @StvFdz. ORCID: 0000-0003-4239-723X

Word counts.

Abstract: 292**.** Main text: 3,626. One figure, three tables, one supplementary table.

Correspondence to: Prof. Esteve Fernández, MD, MPH, PhD. Tobacco Control Unit, Cancer Prevention and Control Programme, Institut Català d'Oncologia (ICO). Avinguda de la Granvia de l'Hospitalet, 199-203, 08908 L'Hospitalet de Llobregat, Barcelona, Spain. Phone: +34 93 2607345. E-mail: efernandez@iconcologia.net (Prof. E. Fernández).

For peer review only

ABSTRACT

Objectives Cancer patients are at higher risk for severe COVID-19 infection. COVID-19 surveillance of workers in oncological centres is crucial to assess infection burden and prevent transmission. We estimate the SARS-CoV-2 seroprevalence among health care workers (HCW) of a comprehensive cancer centre in Catalonia, Spain, and analyse its association with sociodemographic characteristics, exposure factors and behaviours.

Design Cross-sectional study (21st May – 26th June 2020)

Setting A comprehensive cancer centre in Catalonia, Spain,

Participants All HCW (*N*=1,969) were invited to complete an online self-administered epidemiological survey and to provide a blood sample for SARS-CoV-2 antibodies detection.

Primary outcome measure Prevalence (%) and 95% confidence intervals (CI) of seropositivity together with adjusted prevalence ratios (aPR) and 95%CI were estimated.

Results A total of 1,266 HCW filled the survey (participation rate: 64.0%) and 1,238 underwent serological testing (97.8%). The median age was 43.7 years (p25-p75: 34.8-51.0 years), 76.0% were female, 52.0% were nursing or medical staff, and 79.0% worked on-site during the pandemic period. SARS-CoV-2 seroprevalence was 8.9% (95%CI: 7.44-10.63), with no differences by age and sex. No significant differences in terms of seroprevalence were observed between onsite workers and teleworkers. Seropositivity was associated with living with a person with COVID-19 (aPR: 3.86, 95%CI: 2.49-5.98). Among on-site workers, seropositive participants were twofold more likely to be nursing or medical staff. Nursing and medical staff working in a COVID-19 zone showed a higher seroprevalence than other staff (aPR: 2.45, 95% CI: 1.08-5.52).

Conclusions At the end of the first wave of the pandemic in Spain, SARS-CoV-2 seroprevalence among ICO HCW was lower than the reported in other Spanish hospitals. The main risk factors were sharing household with infected people and contact with COVID-19 patients and colleagues. Strengthening preventive measures and health education among HCW is fundamental.

Keywords SARS-CoV-2; COVID-19; seroprevalence; antibody; health care workers; epidemiology.

For peer review only

For peer review only - http://bmjopen.bmj.com/site/about/guidelines.xhtml

ARTICLE SUMMARY

Strenghts and limitations

- This study is the first seroepidemiological study with such a large sample size settled in an oncological health centre and has had a high response rate (64.3%)
- Questionnaire completeness was very high, with no variables presenting more than 5% of missing values.
- Some recall bias is possible as the data for the correlates of SARS-CoV-2 infection rely on a self-administered questionnaire. Also, results regarding the accomplishment of preventive measures, might be overestimated.
- Response and perception biases must be considered, as well as complacency bias.
- Answers reported in the questionnaire could be influenced by the participants' knowledge regarding their COVID status.

Highlights

- First SARS-CoV-2 health care workers seroprevalence study in an oncological monographic centre
- Health care workers seroprevalence knowledge may help hospitals to characterize risk and reduce the risk of infection.
- Protecting HWC health is of paramount importance for reducing morbidity and mortality, reducing transmission, and maintaining the health system capacity
- Strengthening preventive measures among health care workers is fundamental in oncological settings.

For peer review only

INTRODUCTION

Frontline health care workers (HCW) dealing with COVID-19 have higher exposure to SARS-CoV-2 than the general population (1) and they can contribute to the spread of COVID-19 as per their exposure to vulnerable patients. Data regarding the prevalence of SARS-CoV-2 infections in HCW are scarce, variable, and characterized by underlying limitations related to the lack of information on tests performance (2). The European Centre for Disease Prevention and Control (ECDC) has analysed surveillance data with known HCW status from 15 countries in Europe, reporting an overall percentage of HCW among COVID-19 cases of 23.0% but no data on prevalence by workplace or speciality is available (3). In Spain the HCW have been highly affected: a total amount of 40,921 cases among HCW were already officially notified by the 11th of May 2021(4) at the end of the third wave.

Cancer patients are vulnerable, presenting a high risk for COVID-19 infection and more severe outcomes due to their immunosuppression status (5). The pandemic has presented unprecedented professional and personal challenges for the oncology community (6). Data are lacking on the seroprevalence of SARS-CoV-2 among HCW in oncological centres. The present study aims to estimate the seroprevalence of SARS-CoV-2 and associated sociodemographic and behavioural risk factors among workers of the Catalan Institute of Oncology (ICO), a Comprehensive Cancer Centre comprised of four hospitals in Catalonia (Spain), covering around 40% of the adult population in Catalonia (7).

PARTICIPANTS AND METHODS

Study design and setting

A cross-sectional study including blood sample collection and a self-administered questionnaire was conducted between 21st May and 26th June 2020 in the four ICO centres (L'Hospitalet de Llobregat, Badalona, Tarragona/Terres de l'Ebre and Girona).

The study population were HCW delivering care and services to patients (directly or indirectly), and support staff, including those who do not deliver care but work in other tasks within the hospital. A total of 1,969 employees of ICO were invited to participate in the study through an email that allowed access to the study information. The inclusion criteria were: a)

For peer review only - http://bmjopen.bmj.com/site/about/guidelines.xhtml

to be an active worker during the epidemic period, (1st February - 26th June 2020) and b) to be aged ≥18years. The participants filled in an online epidemiological questionnaire and were scheduled for serology testing by the Occupational Health Department. 1,266 HCW filled in the online epidemiological questionnaire (participation rate: 64.3%) and 1,238 of them (97.8%) underwent a serology test. Three participants with inconclusive serological results were excluded. The final analysis included 1,235 participants (**Figure 1**).

Figure 1 about here

For peer review only

Epidemiological questionnaire and study variables

An epidemiological questionnaire was programmed online to collect information regarding sociodemographic characteristics, working information, compliance of personal protective equipment (PPE) measures at work, at home and history of previous COVID-19 infection.

Sociodemographic characteristics included information on age and sex and ICO centre of recruitment, presence of comorbidities, smoking history, pregnancy and cohabitants.

Work-related conditions included the professional category, teleworking status, type of shift, working on a COVID19 zone, contact with COVID-19 cases, contact with biological samples and reporting to be exposed to COVID-19.

In relation to PPE measures at work, participants were asked about feeling protected with PPE and compliance of PPE measures. In respect of preventive measures at home, participants were asked about using face mask when shopping, shower and clothes changing after work or upon home arrival and hand cleaning. Among those participants reporting cohabitants, information about COVID-19 cases and protective measures were also collected. Participants were also asked about the type of transport used to go to work.

Participants were asked about a previous diagnose (and date) of COVID-19 by rRT-PCR or serology, as well as reporting COVID-19 compatible symptoms, and the type of symptoms.

SARS-CoV-2 laboratory testing

Serum samples from participants at L'Hospitalet, Girona and Tarragona/Terres de l'Ebre were studied at the Microbiology Department of Hospital de Bellvitge; whereas samples from health-care workers at ICO Badalona were analysed at the MetroNord Regional Clinical Laboratory. Detection of SARS-CoV-2 antibodies was carried out using the quantitative SARS-CoV-2 S1/S2 IgG LIAISON® test (DiaSorin, Vercelli, Italy) on the LIAISON XL platform, following the manufacturer's instructions. This test discriminates among negative (<12AU/mL; with 3.8 as IgG detection limit), equivocal (12.0–15.0AU/mL) and positive (>15.0AU/mL) subjects. In those cases in which a) IgG anti S1/S2 quantification was higher than the limit of detection (i.e.>3.8AU/mL) but did not reach the limit of discrimination (i.e.<15AU/mL) and/or b) when the healthcare workers answered the questionnaire saying that he or she had been diagnosed of COVID-19 but IgG anti S1/S2 where lower than 15

For peer review only - http://bmjopen.bmj.com/site/about/guidelines.xhtml

AU/ml, an additional serological study was performed using a different antigen (N) as a target. In this case, a SARS-CoV-2 IgG test (Abbott Diagnostics, Sligo, Ireland) was run on an Architect i2000 platform. This test discriminates among negative (<1.4Index (S/C)) and positive (≥1.4Index (S/C)) subjects.

Case definition

A seropositive case of SARS-CoV-2 was defined as seropositivity to IgG independently of previous self-reported results.

Patient and Public Involvement

No patient involved

Statistical analysis

Crude global and by subgroups SARS-CoV-2 seroprevalences and their 95% confidence intervals (CI) were calculated. Differences in the distribution of study variables between seropositive and seronegative participants were assessed by means of chi-squared test for categorical variables, and parametric or non-parametric tests were performed for normal and non-normal continuous variables, respectively. Prevalence ratios (PR) and 95% confidence intervals (CI) were estimated using Poisson regression models with robust variance(8). Prevalence ratios were adjusted (aPR) for statistically significant variables in the bivariate analysis and those considered relevant for the study design. Thus, adjusted models include sex, ICO centre of recruitment, age, type of HCW, teleworking and cohabitants. Linear trends of number of symptoms among those reporting COVID-19 compatible symptoms when rRT-PCR was performed was assessed by fitting the model with the ordinal variable as a continuous. *P*-values were based on 2-sided hypothesis tests and considered significant at $p<0.05$. All analyses were conducted by using Stata version 16.0 (StataCorp LP, College Station, Texas).

Ethical considerations

The present study was approved by the Hospital Universitari de Bellvitge Ethics Committee (PR205/20). The study follows the Helsinki Declaration and subsequent amendments, and Spanish data confidentiality laws (General data protection regulation Organic Law 3/2018, the EU General data protection Regulation 2016/679 and Law 14/2007 for biomedical

For peer review only - http://bmjopen.bmj.com/site/about/guidelines.xhtml

research). All participants signed an informed consent form after receiving information of the study and prior to obtaining biological samples. The biological material obtained was kept at ICO and processed under the appropriate measures in order to preserve the confidentiality of the results and data.

RESULTS

A total of 1,235 HCW with serological results **(Figure 1)** were included in the analysis: 76.0% were female, the median age was 43.7 years (p25-p75: 34.8-51.0 years), 52.2% were nursing or medical staff and 18.6% of the participants teleworked full-time during the study period (**Table 1**). No differences in baseline characteristics between the participants' teleworking and the rest were found (data not shown). Up to 14.7% of the participants reported at least one comorbidity. Regarding smoking habits, 16.0% were current smokers and 28.2% reported to be former smokers (**Table 1**). Seven women were pregnant and none of them showed seropositivity.

The overall crude SARS-CoV-2 seroprevalence was 8.9% (95%CI: 7.44-10.63), with no statistically significant differences by neither age group nor sex, and the seroprevalence for nursing and medical staff was 11.6% (95%CI: 9.37-14.34). After fully adjustment, the main determinants of higher seroprevalence included working at ICO Girona compared to workers at ICO L'Hospitalet (aPR: 1.52, 95%CI: 0.97-2.38), and nursing or medical staff compared to other groups (aPR: 2.04, 95%CI: 1.33-3.14) (**Table 1**).

For peer review only

Table 1. Sociodemographic characteristics associated with SARS-CoV-2 positive serology among study participants (*N*=1,235).

	Total participants *n* (%)	SARS-CoV-2 seroprevalence *n* (%)	Prevalence (95%CI)	*p-value* [6]	aPR (95% CI) [7]
**Study participants**	1,235	110	8.91 (7.44-10.63)		
**Sex**					
Male	291 (23.6)	27 (24.5)	9.28 (6.44-13.20)		REF
Female	939 (76.0)	83 (75.5)	8.84 (7.18-10.83)	*0.82*	0.82 (0.53-1.28)
**Age [median, (p25-p75)]**	43.7 (34.8-51.0)	42.8 (32.0-50.1)		*0.62*	0.99 (0.97-1.01)
<35 years	313 (25.3)	33 (30.0)	10.54 (7.59-14.46)		REF
35-49 years	566 (45.8)	47 (42.7)	8.30 (6.29-10.88)		0.85 (0.55-1.34)
>49 years	356 (28.8)	30 (27.3)	8.43 (5.95-11.80)	*0.5*	0.88 (0.53-1.46)
**ICO Center**					
ICO L'Hospitalet	885 (71.7)	73 (66.4)	8.25 (6.61-10.25)		REF
ICO Girona	204 (16.5)	29 (26.4)	14.22 (10.06-19.72)		1.52 (0.97-2.38)
ICO Badalona	134 (10.9)	7 (6.4)	5.22 (2.51-10.56)		0.54 (0.25-1.19)
ICO Tarragona / Terres de l'Ebre	12 (1.0)	1 (0.9)	8.33 (1.16-41.38)	*0.02*	1.07 (0.15-7.83)
**Professional category**					
Nursing staff[1]	380 (30.8)	43 (39.0)	11.32 (8.50-14.92)		REF
Medical Staff[2]	265 (21.5)	32 (29.1)	12.08 (8.67-16.58)		1.07 (0.65-1.76)
Middle and superior technicians	285 (23.1)	14 (12.7)	4.91 (2.93-8.13)		0.41 (0.22-0.77)
Service staff[3]	114 (9.2)	2 (1.8)	7.02 (3.55-13.42)		0.69 (0.31-1.54)
Watchmen	21 (1.7)	8 (7.3)	9.52 (2.39-31.16)		0.74 (0.17-3.24)
Administratives	129 (10.4)	8 (7.3)	6.20 (3.13-11.92)		0.54 (0.25-1.16)
Other	20 (1.6)	1 (0.9)	5.00 (0.70-28.26)	*0.03*	0.50 (0.07-3.71)
Nursing or medical staff[4]	645 (52.2)	75 (68.2)	11.63 (9.37-14.34)	*<0.001*	2.04 (1.33-3.14)
Other staff[5]	569 (46.1)	33 (30.0)	5.80 (4.15-8.05)		REF
**Telework**					
Never/Occasionally	981 (79.4)	86 (78.1)	8.77 (7.15-10.71)		REF
Always	230 (18.6)	23 (20.9)	10.00 (6.72-14.63)	*0.56*	1.60 (0.98-2.59)

For peer review only - http://bmjopen.bmj.com/site/about/guidelines.xhtml

Table 1 (continued)

	Total participants	SARS-CoV-2 seroprevalence	Prevalence (95%CI)	*p-value*[7]	aPR (95% CI)[8]
**Shift work**					
Morning	545 (44.1)	49 (45.0)	8.99 (6.86-11.7)		REF
Evening	140 (11.3)	10 (9.1)	7.14 (3.88-12.77)		0.56 (0.34-0.93)
Split shift (morning-evening)	417 (33.8)	38 (34.5)	9.11 (6.7-12.28)		0.88 (0.57-1.37)
Night	88 (7.1)	10 (9.1)	11.36 (6.22-19.86)		0.95 (0.46-1.96)
Other	25 (2)	3 (2.7)	12 (3.92-31.32)	*0.83*	1.15 (0.35-3.75)
**Comorbidities**[6]					
None	1,054 (85.3)	99 (90.0)	9.39 (7.77-11.31)		REF
Yes	181 (14.7)	11 (10.0)	6.08 (3.4-10.64)	*0.15*	0.67 (0.36-1.25)
**Smoking history**					
Never	650 (52.6)	80 (72.7)	12.31 (9.99-15.07)		REF
Past	348 (28.2)	22 (20.0)	6.32 (4.20-9.42)		0.57 (0.35-0.93)
Current	198 (16.0)	8 (7.3)	4.04 (2.03-7.87)	*0.0002*	0.38 (0.18-0.79)
**Cohabitants**					
Yes	1,119 (90.6)	95 (86.0)	8.49 (6.99-10.27)		REF
No	104 (8.4)	15 (13.6)	14.42 (8.88-22.57)	*0.04*	1.48 (0.83-2.66)

Numbers do not always sum up the total due to some missing values (none of the categories present more than 5% of missing values).
aPR: adjusted Prevalence Ratio, CI: Confidence Interval, p25: 25% percentile, p75: 75% percentile.
[1] Nursing staff: nurses and nursing assistants.
[2] Medical staff: resident physicians and specialists.
[3] Service staff: security, maintenance, cleaning and kitchen.
[4] Nurses, nursing assistants, resident physicians and specialists.
[5] Middle and superior technicians, security, maintenance, cleaning, kitchen, watchmen, administrative, and other.
[6] Comorbidities: hypertension, obesity (BMI≥30), heart disease, liver disease, diabetes, chronic respiratory disease, renal disease, cancer, autoimmune disorders and other immunological disorders.
[7] Chi-squared test for categorical variables (Fisher's exact test corrected for continuity) and median test for continuous variables.
[8] Adjusted for sex, age (continuous), ICO centre, type of health care workers, telework and cohabitants.

For peer review only - http://bmjopen.bmj.com/site/about/guidelines.xhtml

Seroprevalence among on-site workers was 8.8% (95%CI:7.15-10.71) (**Table 2**). Onsite workers were younger, mostly health care workers, and reported more frequently rRT-PCR previous to serology than teleworkers but no differences were observed in sex, self-reported comorbidities, smoking history, cohabiting with COVID-19 positive case between them and teleworkers (data not shown). Among this group (*N*=981) of professionals who never or occasionally teleworked SARS-CoV-2 seropositivity was not associated with not working in a COVID-19 zone (aPR: 1.29, 95%CI: 0.81-2.06), nor being in contact with COVID-19 biological samples (aPR: 1.30, 95%CI: 0.77-2.20) nor being in contact with patient with COVID-19 (aPR: 1.09, 95%CI: 0.66-1.79) were associated with SARS-CoV-2 positivity (**Table 2**). On-site nursing or medical staff who worked in a COVID zone had twofold SARS-CoV-2 seroprevalence than others who did not work in COVID zone (aPR: 2.45, 95%CI: 1.08-5.52). Seropositivity was higher among those who referred being exposed by interacting with colleagues (aPR: 3.26, 95%CI: 1.49-7.15). On-site workers who self-reported symptoms of COVID-19 were almost 10-fold more likely to be seropositive than those who did not (aPR: 9.5, 95%CI: 5.34-17.03). Most of on-site workers were highly adherent to the recommendation of hand hygiene at work. Hand-washing before eating or working were followed by more than 97% of on-site workers, whereas around 24% of them reported not hand hygiene after working or a low frequency of hand washing during the workday. In relation to protective measures at work 17.4% of the on-site workers did not feel protected with PPE and 12.1% did not use PPE with confirmed or suspicious COVID-19 cases. In reference to colleagues' behaviour, 2m safety distance from colleagues when having lunch was reported to be unfollowed by 14.1% (**Table 2**).

For peer review only

Table 2. Occupational factors associated with SARS-CoV-2 positive serology among on-site workers (*N*=981).

	Total participants *n* (%)	SARS-CoV-2 seroprevalence *n* (%)	Prevalence (95%CI)	*p-value* [2]	Adjusted PR (95% CI) [3]
**On-site workers**	981 (79.4)	86 (78.1)	8.77 (7.15-10.71)	*0.56*	
**Type of transport to work**					
Private	751 (76.6)	66 (76.7)	8.79 (6.96-11.04)		REF
Public	154 (15.7)	15 (17.4)	9.74 (5.95-15.54)		1.32 (0.74-2.36)
Private and Public	35 (3.6)	2 (2.3)	5.71 (1.43-20.19)		0.63 (0.15-2.58)
Walking	37 (3.8)	3 (3.5)	8.11 (2.63-22.34)	*0.89*	0.57 (0.14-2.35)
**Working in a COVID-19 zone**					
No	398 (40.6)	29 (33.7)	7.29 (5.11-10.29)		REF
Yes	545 (55.6)	55 (63.9)	10.09 (7.83-12.92)	*0.14*	1.29 (0.81-2.06)
**Type of and COVID zone** [1]					
Non-assisting HCW & never worked in a COVID-19 zone	148 (15.1)	7 (8.0)	4.73 (2.27-9.6)		REF
Non-assisting HCW & ever worked in a COVID-19 zone	230 (23.4)	13 (15.1)	5.65 (3.31-9.5)		1.12 (0.44-2.82)
Assisting HCW & never worked in a COVID-19 zone	244 (24.9)	22 (25.6)	9.02 (6.01-13.32)		1.81 (0.77-4.26)
Assisting HCW & ever worked in a COVID-19 zone	311 (31.7)	40 (46.5)	12.86 (9.57-17.07)	*0.006*	2.45 (1.08-5.52)
p-trend					0.26
**Contact with COVID-19 cases**					
No	333 (33.9)	23 (26.7)	6.91 (4.63-10.18)		REF
Yes	536 (54.6)	57 (66.3)	10.63 (8.29-13.54)	*0.07*	1.30 (0.77-2.20)
**Contact with COVID-19 biological samples**					
No	646 (65.9)	51 (59.3)	7.89 (6.05-10.24)		REF
Yes	282 (28.7)	30 (34.9)	10.64 (7.54-14.81)	*0.17*	1.09 (0.66-1.79)
**Reporting to be exposed to COVID-19 by interacting with colleagues at work**					
No	242 (24.7)	66 (76.7)	2.89 (1.38-5.95)		REF
Yes	608 (62.0)	7 (8.1)	10.86 (8.62-13.59)	*<0.0001*	3.26 (1.49-7.15)
**Reporting COVID-19 compatible symptoms**					
No	623 (63.5)	15 (17.4)	2.41 (1.46-3.96)		REF
Yes	306 (31.2)	68 (79.1)	22.22 (17.91-27.23)	*<0.0001*	9.53 (5.34-17.03)

For peer review only - http://bmjopen.bmj.com/site/about/guidelines.xhtml

Table 2 (continued)

	Total participants	SARS-CoV-2 seroprevalence	Prevalence (95%CI)	*p-value* [2]	Adjusted PR (95% CI) [3]
**Not following protection measures at work**					
Felt protected with PPE	132 (17.4)	12 (16.9)	9.09 (5.23-15.34)	*0.83*	0.98 (0.51-1.88)
Colleagues cover themselves with their elbows when sneezing/coughing	155 (15.8)	21 (24.4)	13.55 (9.00-19.90)	*0.01*	1.70 (1.01-2.87)
2m safety distance from colleagues during lunch	127 (14.1)	12 (15.6)	9.45 (5.44-15.91)	*0.71*	1.06 (0.56-1.99)
Use of PPE with confirmed or suspicious COVID-19 patients	79 (12.1)	7 (10.45)	8.86 (4.28-17.46)	*0.63*	1.01 (0.45-2.26)
PPE removal safety	48 (7.3)	3 (4.6)	6.25 (2.03-17.68)	*0.33*	0.54 (0.17-1.74)
Personal use of mask	34 (3.5)	1 (1.2)	2.94 (0.41-18.17)	*0.21*	0.41 (0.06-2.99)
Colleagues use of surgical mask	7 (0.7)	1 (1.2)	14.29 (1.96-58.12)	*0.62*	1.68 (0.23-12.29)
**Not following hand hygiene at work**					
≤7 times during workday	233 (23.8)	15 (17.4)	6.44 (3.92-10.41)	*0.13*	0.71 (0.39-1.28)
After money, phone and other personal tools manipulation	175 (17.8)	16 (18.6)	9.14 (5.67-14.41)	*0.89*	1.00 (0.58-1.74)
Every time entering in a new workspace	102 (10.4)	5 (5.8)	4.90 (2.05-11.25)	*0.14*	0.55 (0.22-1.37)
Before working	21 (2.1)	3 (3.5)	14.29 (4.67-36.17)	*0.37*	1.72 (0.54-5.47)
After finishing the workday	17 (1.7)	1 (1.2)	5.88 (0.82-32.09)	*0.67*	0.65 (0.09-4.72)
Before eating	9 (0.9)	2 (2.3)	22.22 (5.59- 57.95)	*0.16*	2.67 (0.65-10.94)

Numbers do not always sum up the total due to some missing value (none of the categories present more than 5% of missing values).
PR: Prevalence Ratio, CI: Confidence Interval, HCW: Health Care Workers.
[1] Assisting HCW: nurses, nursing assistants, resident physicians and specialists; otherwise classified and non-assisting HCW.
[2] Chi-squared test.
[3] Adjusted for sex, age (continuous), ICO centre, care staff, telework and cohabitants.

For peer review only - http://bmjopen.bmj.com/site/about/guidelines.xhtml

Concerning the correlates of seropositivity according to household factors for all participants (**Table 3**), seropositivity was associated with living with a COVID-19 positive person (aPR: 3.86, 95%CI: 2.49-5.98). Up to 17.3% of the participants did not take a shower nor changed clothes upon arrival, but the majority (99.0%) did hand hygiene upon arrival. The least followed hand hygiene home practices were after money, phone and other personal tools manipulation as well as after nose blowing (23.5% and 22.7%). However, not following protection measures or hand hygiene at home were associated to a higher SARS-CoV-2 seroprevalence.

For peer review only

Table 3. Household factors associated with SARS-CoV-2 positive serology among study participants (n=1,235).

	Total participants *n* (%)	SARS-CoV-2 seroprevalence *n* (%)	Prevalence (95%CI)	*p-value* [2]	Adjusted PR (95% CI) [3]
**Study participants**	1,235	110	8.91 (7.44-10.63)		
**Cohabitants with COVID-19**[1]					
No	894 (79.9)	52 (54.7)	5.82 (4.46-7.56)		REF
Yes	141 (12.60)	34 (35.8)	24.11 (17.76-31.86)	*<0.0001*	3.86 (2.49-5.97)
**Cohabitants cover themselves with their elbow when sneezing**					
No	158 (14.1)	18 (18.9)	11.39 (7.29-17.37)		REF
Yes	919 (82.1)	73 (76.8)	7.94 (6.36-9.88)	*0.15*	0.73 (0.43-1.22)
**Not following protection measures at home**[4]					
Use of face mask when shopping	17 (1.4)	2 (1.8)	11.76 (2.95-36.86)	*0.67*	0.98 (0.24-4.05)
Shower and clothes changing afterwork or upon home arrival	214 (17.3)	20 (18.2)	9.35 (6.11-14.05)	*0.82*	1.02 (0.62-1.69)
**Not following hand hygiene at home**[4]					
Upon arrival	12 (1)	2 (1.8)	16.67 (4.19-47.76)	*0.35*	1.59 (0.39-6.60)
Before eating	60 (4.9)	9 (8.2)	15.00 (7.99-26.4)	*0.09*	1.55 (0.77-3.12)
After money, phone and other personal tools manipulation	290 (23.5)	27 (24.6)	9.31 (6.46-13.24)	*0.71*	1.01 (0.65-1.58)
After cleaning	110 (8.9)	8 (7.3)	7.27 (3.68-13.88)	*0.53*	0.78 (0.38-1.61)
After nose blowing	280 (22.7)	25 (22.7)	8.93 (6.1-12.88)	*0.99*	0.93 (0.58-1.48)

Numbers do not always sum up the total due to some missing values (none of the categories present more than 5% of missing values).
PR: Prevalence Ratio, CI: Confidence Interval.
[1] Analyses performed among those participants who reported having cohabitants (n=1,119).
[2] Chi-squared test.
[3] Adjusted for sex, age (continuous), ICO center, care staff, telework and cohabitants.
[4] Unfollowing the measures of protection and hand hygiene recommendations.

For peer review only - http://bmjopen.bmj.com/site/about/guidelines.xhtml

Clinical characteristics were collected for those participants (N=469) who reported a rRT-PCR performed previous to serology (**Suppl. table 1**). The majority of the patients with a positive serology and reporting a positive rRT-PCR presented compatible COVID-19 symptoms (74.4%). Among seropositive patients, the most common symptoms were arthromyalgia, cough, headache, asthenia and anosmia. Reporting a positive rRT-PCR when presenting compatible symptoms, was associated with a threefold higher prevalence of seropositivity (aPR: 3.10, 95%CI: 1.78-5.31). An increased number of compatible symptoms was also associated with a higher seroprevalence (aPR: 7.4, 95%CI: 1.78-5.31, for presenting 4 or more symptoms as compared to no symptoms).

DISCUSSION

Despite the impact of COVID-19 in oncological patients (9), there are no SARS-CoV-2 seroprevalence studies in comprehensive cancer centres. The global SARS-CoV-2 seroprevalence was 8.9%, lower than expected, owing to the presumed higher risk among HCW. Also, it was lower than the reported estimates in two studies performed among HCW in Catalonia between March-April and May 2020, showing a seroprevalence of 11.2% (10) and 10.3% (11) respectively. In both cases, the seroprevalence was higher than in the general population, estimated to be of a maximum of 7.4% in the Barcelona metropolitan area when the study was conducted (12), but lower than expected among these highly exposed populations. Seroprevalence studies interpretation must be related to the average COVID-19 prevalence at the time of blood collection, and both of the mentioned studies were carried out earlier in time than ours, which was performed approximately one month later (21st May-26th June 2020), and specifically two months after the first-wave peak in Catalonia (23th March) (13). An explanation for this lower seroprevalence in our Centre concerns the participation: all active HCW, regardless their teleworking status during the previous months or work absenteeism, were invited to participate, and most did (64%). In contrast, García-Basteiro's (10) and Barallat's (11) studies comprised general hospitals (10,11) and primary health care centers (11) in which the incidence could be higher than in a cancer monographic centre.

In comparison with other Spanish seroprevalence studies, our estimate was even lower. A study performed among 2,509 HCW in the Alcorcón Hospital (Madrid), in April 2020, found a seroprevalence of 31.6% (14). A partial explanation for this large prevalence was the higher

For peer review only - http://bmjopen.bmj.com/site/about/guidelines.xhtml

exposure to the virus in this particular geographical area during the first wave of the epidemic. The largest population-based cross-sectional study in Spain, conducted from April 27th to May 11th to estimate the seroprevalence of SARS-CoV-2 infection showed a nationwide prevalence of 5.0% in adults, being of 6.8% in Barcelona and 11.5% in the Madrid region (12).

Other countries also reported the SARS-CoV-2 seroprevalence status of HCW, but with a quite broad range of outcomes and no specificity of HCW in oncological premises. Seroprevalence rates among HCW in Germany, Denmark and Belgium were low (1.6%, 4.0% and 6.4%, respectively) (15–17). These studies were conducted during early stages of the epidemic, and therefore, they supposed that infection was community-acquired. Also, the Belgian study, with a sample size of almost 30,000 HCW, notes that the high availability of PPE, high standards of infection prevention, and PCR screening in symptomatic staff, coupled with contact tracing and quarantine, might explain the relatively low seroprevalence (17). An study performed in Lombardy region, Italy (18), one of the regions most hit by the first epidemic wave, showed a seroprevalence of 7.4% (3.8-11.0%), similar to the observed in the Catalan studies (10,11). Sweden and the UK were the two European countries reporting the highest seropositivity rates among HCW: 19.1% and between 18.0% and 45.3%, respectively (19–21). In the UK, this high seroprevalence was settled in London during the week with the highest number of new cases in the city in the first wave, with around 15% seropositivity among the general population. In the USA, the prevalence of infection among HCW was 10.7%, despite high variation, as low as 1.1% in California (22) to 13.7% in New York State (23).

The differences observed among different countries and healthcare settings may be explained not only because of the period when the study was performed, but also by the seroprevalence (and the transmission rates) in the community, and the COVID policies stablished (social distancing, hand hygiene, and use of PPEs). Nonetheless, all the seroprevalence estimates among HCW were substantially higher than those reported in the general population of each geographical area during the same study period, firmly suggesting an occupational health hazard among HCW.

Despite SARS-CoV-2 seropositivity rate in oncological HCW has significant implications for oncological patients, scant research has been done. Some of the few studies performed, showed seroprevalence rates at huge variation. The lowest SARS-CoV-2 antibodies rates in

For peer review only - http://bmjopen.bmj.com/site/about/guidelines.xhtml

oncological HCW (1% and 3.6%) were found in Thuringia (Germany) (24) and Vienna (Austria) (25), both areas with a low COVID-19 incidence during the first wave. The highest prevalence (21.2%) was reported in England between March and June 2020, among 70 workers, all patient-facing oncology staff, which may explain the high prevalence (26). All of those studies were based on small sample sizes (<70 participants) in oncological wards, but none, to the best of our knowledge, was conducted in monographic oncological hospitals or comprehensive cancer centres.

In our study, no differences in seroprevalence according to sex, age and presence of comorbidities were found. Current or past smoking was however inversely associated to SARS-CoV-2 seroprevalence. Early studies in selected cohorts of COVID-19 patients showed a paradoxical higher risk of SARS-CoV-2 infection among non-smokers (27) whilst ever smokers showed higher risk of COVID-19 progression, including severity of the disease, Intensive Care Unit admission and death (27,28). The reported prevalence of current smokers in this survey (16.5%) is lower than that reported in the periodical smoking surveys in our centres (ranging 21-26% in 2017-2019, unpublished data) probably due to underreporting of smoking or lack of reporting.

It is worth mentioning that, unlike most of the other published seroepidemiological studies among HCW, the present study was performed among all the HCW of the institution, regardless they did full-time telework during the study period (21.6%). No differences by telework were found, and among all study participants the main factor associated with SARS-CoV-2 seropositivity was living with a COVID-19 case, with a three times higher probability. This finding supports the importance of community dissemination of the infection also for HCWs. Similarly, the Belgian study suggested that neither being directly involved in clinical care nor working in a COVID-19 unit increased the likelihood of being seropositive, while having a suspected COVID-19 household contact did(17).

To avoid the spread of the disease, the only available and effective measures among health care workers during the first and second wave of the epidemic, have been hand hygiene, the use of mask and, when indicated, the use of complete PPE, physical distancing, patients' isolation, contact tracing of cases to quarantine their close contacts and screening them, as well as community based interventions such as screening of high-risk populations, mass quarantine and social or mobility restrictions (29). After December 2020 with the

For peer review only - http://bmjopen.bmj.com/site/about/guidelines.xhtml

authorization of first COVID-19 vaccinations, vaccines to prevent disease have become another useful tool, currently under implementation (30).

An increased risk of infection among HCW has been attributed to direct, close and long-time exposure to large numbers of infected patients, especially those involving certain practices such as intubations or contact with aerosols and body secretions (29,31). Other relevant factors that could contribute to increase the probability of infection among HCW are shortage of PPE (32) and work intensity and lack of rest (due to staff shortages) together with inadequate infection control training (33). Also, it has been observed that most of the HCW infected were working in general wards or first level emergency response departments (32).

Our study shows that among on-site HCW in an oncological centre, working as medical care staff (nursing, nursing assistant, resident physicians and specialists) in COVID-19 areas stood out as one of the main factors associated with developing SARS-CoV-2 antibodies. Published results regarding the possibility of in-hospital infection among HCW and transmission at work are controversial. Some studies did not find any relation between working in COVID unit or professional category with seropositivity (10,18). Korth et al. showed that seroprevalence was higher in the intermediate-risk group (daily non−COVID-19 patient contact) compared to the high-risk group (daily contact to COVID-19 patients on the designated wards and on the intensive care units) (OR: 0.2; p=0.13) (15). A study from Denmark showed that HCW working in COVID-19 wards had a significantly higher seroprevalence than other frontline health-care workers working in hospitals (RR: 1.7; p<0.001). Also, a Swedish study found that seroprevalence was strongly associated with patient-related work (16), COVID-19 patient contact (OR: 1.43, p<0.005), and occupation (as being an assisting nurse, OR: 3.7; p<0.005) (19).

In our study, the HCW who reported being exposed to COVID-19 by other colleagues presented an almost four-fold probability of being seropositive. Most of the HCW declared to follow the protective measures at the workplace. The moments with less accomplishment were at the end of the workday and after tools' manipulation, with no differences according to protective measures and hand hygiene. No differences in seroprevalence were found according to protective measures and hand hygiene.

Contact with colleagues at work is a potentially dangerous situation for transmission among HCW as well as the relaxation of protective measures at the end of the day. Therefore,

For peer review only - http://bmjopen.bmj.com/site/about/guidelines.xhtml

patients with COVID-19 might not be the main source of SARS-CoV-2 infection for HCW, and HCW could be exposed to non-suspected COVID-19 patients, infected family members, social contacts, and colleagues, as a result of the pandemic community transmission (34).

Protecting HWC health is of paramount importance for reducing morbidity and mortality, reducing transmission, and maintaining the health system capacity (35). Thus international health authorities recommend screening strategies for SARS-CoV-2 infection in exposed or high-risk HCW (36) as well as massive COVID-19 vaccination (37).

Significant differences exist in SARS-CoV-2 testing between countries, and existing programmes focus on screening symptomatic rather than asymptomatic staff. Published studies point out the fact that screening should be performed regardless of the absence of typical symptoms for COVID-19 disease. It has been demonstrated that seroconversion can occur in HCW who have suffered no previous symptoms of SARS-CoV-2 infection (38,39) as asymptomatic transmission is very relevant in SARS-CoV-2 spread (40,41). Thus, the approach for mass testing of both symptomatic and asymptomatic HCW could mitigate workforce depletion by unnecessary quarantine, reduce spread in atypical, mild, or asymptomatic cases; and protect patients and health-care workforce.

Among the potential limitations of the study, some recall bias is possible as the data for the correlates of SARS-CoV-2 infection rely on a self-administered questionnaire. Also, response and perception biases have to be considered, as well as complacency bias. Results, especially those regarding the accomplishment of preventive measures, might be overestimated. Answers reported in the questionnaire could be influenced by the participants' knowledge regarding their COVID status. However, this study is the first seroepidemiological study with such a large sample size settled in an oncological health centre. The sufficient sample size and high response rate (64.3%) are strengths of the study, although information regarding non-participants was not collected, and we cannot disregard a potential participation bias. Questionnaire completeness was very high, with no variables presenting more than 5% of missing values.

In conclusion, SARS-CoV-2 seroprevalence among ICO HCW was lower than the reported in other Catalan hospitals, but higher than among the general population living in the area. Whereas the main risk factor was living with infected people, contact with COVID-19 patients and other colleagues were associated with SARS-CoV-2 infection. Knowing the

For peer review only - http://bmjopen.bmj.com/site/about/guidelines.xhtml

seroprevalence rate and follow-up evaluation of persistence may help hospitals to characterize the staff at risk, rationalize their placement, prioritize the use of PPE, thereby potentially reducing the risk of infection. Follow-up studies to evaluate long term durability of antibodies among HCW will be of interest, after the introduction of COVID-19 vaccination among HCW, to better promote infection control in this group. Strengthening preventive measures and health education among HCW is fundamental, especially in oncological departments and centres.

For peer review only

For peer review only - http://bmjopen.bmj.com/site/about/guidelines.xhtml

Contributors EF, DCP, AP, CC, AC and AS contributed to study design. SC, AD, LG, IB, JT, MG, FS, JJT, DC, AS, BC, DR and AP accrued participants and care for blood collection at ICO centres. Laboratory analyses were coordinated by MADL. The questionnaire was designed by DCP and EF, and revised by PPT, ASL, YB, DC, AP, and LA. Questionnaire's implementation was done by EL, JM, JPR, CMM. Data were analysed by YB and DC. PPT, ASL, YB, DC, LA, and EF interpreted the initial results and designed the tables. All authors contributed to interpretation of results. The first draft of the manuscript was prepared by PPT and ASL. PPT, ASL, YB, DC, LA, DC and EF were the main contributors to the writing of the manuscript. All authors assisted in manuscript review. The co-senior authors had full access to all the data in the study for interpretation and had final responsibility for manuscript generation and review, and the decision to submit for publication. EF is the guarantor.

Funding This work was supported by the Catalan Institute of Oncology. PPT is partially supported by the Instituto de Salud Carlos III (ISCIII), Government of the Kingdom of Spain, co-funded by FEDER funds/European Regional Development Fund (ERDF) - a way to build Europe (CM19/00216). The Ministry of Universities and Research, Government of Catalonia partly supports EF and DCP (2017SGR319) and PPT, AS, YBM, DC and LA (2017SGR1085). We thank CERCA Programme / Generalitat de Catalunya for institutional support.

Competing interests JJ received research funding from Kyowa Kirin and from Angelini Pharma for congress attendance. The Cancer Epidemiology Research Programme has received grants from Merck & Co., Roche, GSK, IDT, Hologic, and Seegene.

Acknowledgments The authors acknowledge all the health care workers who participated in the study as well as all the ICO staff involved in the logistics of the different aspects of the study.

Data availability statement No additional data available

For peer review only - http://bmjopen.bmj.com/site/about/guidelines.xhtml

REFERENCES

1. Ng K, Poon BH, Kiat Puar TH, Shan Quah JL, Loh WJ, Wong YJ, et al. COVID-19 and the Risk to Health Care Workers: A Case Report. Annals of internal medicine. 2020 Jun 2;172(11):766–7.
2. Lahner E, Dilaghi E, Prestigiacomo C, Alessio G, Marcellini L, Simmaco M, et al. Prevalence of Sars-Cov-2 infection in health workers (HWs) and diagnostic test performance: the experience of a teaching hospital in central Italy. International Journal of Environmental Research and Public Health. 2020 Jun 2;17(12):1–12.
3. Risk factors and risk groups [Internet]. [cited 2021 Mar 19]. Available from: https://www.ecdc.europa.eu/en/covid-19/latest-evidence/epidemiology
4. Informe sobre la situación de COVID-19 en personal sanitario en España. España; 2020 May.
5. Dai M, Liu D, Liu M, Zhou F, Li G, Chen Z, et al. Patients with cancer appear more vulnerable to SARS-CoV-2: A multicenter study during the COVID-19 outbreak. Cancer Discovery. 2020 Jun 1;10(6):783.
6. Leung MST, Lin SG, Chow J, Harky A. COVID-19 and Oncology: Service transformation during pandemic. Cancer Medicine. 2020 Oct 18;9(19):7161–71.
7. Institut Català d'Oncologia [Internet]. [cited 2021 Mar 19]. Available from: http://ico.gencat.cat/ca/l_institut/
8. Barros AJD, Hirakata VN. Alternatives for logistic regression in cross-sectional studies: An empirical comparison of models that directly estimate the prevalence ratio. BMC Medical Research Methodology. 2003 Oct 20;3:1–13.
9. Richards M, Anderson M, Carter P, Ebert BL, Mossialos E. The impact of the COVID-19 pandemic on cancer care. Nature Cancer. 2020 Jun;1(6):565–7.
10. Garcia-Basteiro AL, Moncunill G, Tortajada M, Vidal M, Guinovart C, Jiménez A, et al. Seroprevalence of antibodies against SARS-CoV-2 among health care workers in a large Spanish reference hospital. Nature Communications. 2020 Dec 1;11(1):1–9.
11. Barallat J, Fernández-Rivas G, -Sánchez B Q, Martinez-Caceres. Seroprevalence of SARS-CoV-2 IgG Specific Antibodies among Healthcare Workers in the Northern Metropolitan Area of Barcelona, Spain, after the first pandemic wave.
12. Pollán M, Pérez-Gómez B, Pastor-Barriuso R, Oteo J, Hernán MA, Pérez-Olmeda M, et al. Prevalence of SARS-CoV-2 in Spain (ENE-COVID): a nationwide, population-based seroepidemiological study. The Lancet. 2020 Aug 22;396(10250):535–44.
13. COVID-19 data and indicators. Sistemes d'Informació dels Serveis d'Atenció Primària-SISAP, Institut Català de la Salut, Departament de Salut, Generalitat de Catalunya. [Internet]. 2020 [cited 2021 Mar 19]. Available from: http://eines.portalics/covid/
14. Galán MAI, Velasco M, Casas MAL, Goyanes MAJ, Rodríguez-Caravaca G, Losa JE, et al. SARS-CoV-2 Seroprevalence among all workers in a teaching hospital in Spain: Unmasking the risk. medRxiv. medRxiv; 2020. p. 2020.05.29.20116731.
15. Korth J, Wilde B, Dolff S, Anastasiou OE, Krawczyk A, Jahn M, et al. SARS-CoV-2-specific antibody detection in healthcare workers in Germany with direct contact to COVID-19 patients. Journal of Clinical Virology. 2020 Jul 1;128:104437.
16. Iversen K, Bundgaard H, Hasselbalch RB, Kristensen JH, Nielsen PB, Pries-Heje M, et al. Risk of COVID-19 in health-care workers in Denmark: an observational cohort study. The Lancet Infectious Diseases. 2020;20(12):1401–8.
17. Steensels D, Oris E, Coninx L, Nuyens D, Delforge ML, Vermeersch P, et al. Hospital-Wide SARS-CoV-2 Antibody Screening in 3056 Staff in a Tertiary Center in Belgium. JAMA - Journal of the American Medical Association. 2020;324(2):195–7.

For peer review only - http://bmjopen.bmj.com/site/about/guidelines.xhtml

18. Sotgiu G, Barassi A, Miozzo M, Saderi L, Piana A, Orfeo N, et al. SARS-CoV-2 specific serological pattern in healthcare workers of an Italian COVID-19 forefront hospital. BMC Pulmonary Medicine. 2020;20(1):1–6.
19. Rudberg AS, Havervall S, Månberg A, Jernbom Falk A, Aguilera K, Ng H, et al. SARS-CoV-2 exposure, symptoms and seroprevalence in healthcare workers in Sweden. Nature Communications. 2020 Dec 1;11(1):1–8.
20. Pallett SJC, Rayment M, Patel A, Fitzgerald-Smith SAM, Denny SJ, Charani E, et al. Point-of-care serological assays for delayed SARS-CoV-2 case identification among health-care workers in the UK: a prospective multicentre cohort study. The Lancet Respiratory Medicine. 2020;8(9):885–94.
21. Houlihan CF, Vora N, Byrne T, Lewer D, Heaney J, Moore DA, et al. SARS-CoV-2 virus and antibodies in front-line Health Care Workers in an acute hospital in London: Preliminary results from a longitudinal study. medRxiv. medRxiv; 2020. p. 2020.06.08.20120584.
22. Brant-Zawadzki M, Fridman D, Robinson PA, Zahn M, Chau C, German R, et al. SARS-CoV-2 antibody prevalence in health care workers: Preliminary report of a single center study. PLoS ONE. 2020 Nov 1;15(11 November).
23. Moscola J, Sembajwe G, Jarrett M, Farber B, Chang T, McGinn T, et al. Prevalence of SARS-CoV-2 Antibodies in Health Care Personnel in the New York City Area. JAMA - Journal of the American Medical Association. 2020;324(9):893–5.
24. Epstude J, Harsch IA. Seroprevalence of COVID-19 antibodies in the cleaning and oncological staff of a municipal clinic Seroprävalenz von COVID-19-Antikörpern beim Raumpflegepersonal und beim Personal einer onkologischen Station in einer kommunalen Klinik. 2020;15:5–9.
25. Fuereder T, Berghoff AS, Heller G, Haslacher H, Perkmann T, Strassl R, et al. SARS-CoV-2 seroprevalence in oncology healthcare professionals and patients with cancer at a tertiary care centre during the COVID-19 pandemic. ESMO Open. 2020 Sep 2;5(5):e000889.
26. Favara DM, Cooke A, Doffinger R, McAdam K, Corrie P, Ainsworth NL. COVID-19 Serology in Oncology Staff Study: Understanding SARS-CoV-2 in the Oncology Workforce. Vol. 33, Clinical Oncology. Elsevier Ltd; 2021. p. e61–3.
27. Simons D, Shahab L, Brown J, Perski O. The association of smoking status with SARS-CoV-2 infection, hospitalisation and mortality from COVID-19: A living rapid evidence review with Bayesian meta-analyses (version 11). Qeios. 2021 Mar 2;
28. Patanavanich R, Glantz SA. Smoking is associated with COVID-19 progression: A meta-analysis. Vol. 22, Nicotine and Tobacco Research. Oxford University Press; 2020. p. 1653–6.
29. Chou R, Dana T, Buckley DI, Selph S, Fu R, Totten AM. Epidemiology of and Risk Factors for Coronavirus Infection in Health Care Workers: A Living Rapid Review. Vol. 173, Annals of internal medicine. NLM (Medline); 2020. p. 120–36.
30. Coronavirus disease (COVID-19): Vaccines [Internet]. [cited 2021 Mar 19]. Available from: https://www.who.int/news-room/q-a-detail/coronavirus-disease-(covid-19)-vaccines
31. Nguyen LH, Drew DA, Joshi AD, Guo CG, Ma W, Mehta RS, et al. Risk of COVID-19 among frontline healthcare workers and the general community: A prospective cohort study. medRxiv. medRxiv; 2020. p. 2020.04.29.20084111.
32. Wang D, Hu B, Hu C, Zhu F, Liu X, Zhang J, et al. Clinical Characteristics of 138 Hospitalized Patients with 2019 Novel Coronavirus-Infected Pneumonia in Wuhan, China. JAMA - Journal of the American Medical Association. 2020 Mar 17;323(11):1061–9.

For peer review only - http://bmjopen.bmj.com/site/about/guidelines.xhtml

33. Wang J, Zhou M, Liu F. Reasons for healthcare workers becoming infected with novel coronavirus disease 2019 (COVID-19) in China. Vol. 105, Journal of Hospital Infection. W.B. Saunders Ltd; 2020. p. 100–1.
34. Bielicki JA, Duval X, Gobat N, Goossens H, Koopmans M, Tacconelli E, et al. Monitoring approaches for health-care workers during the COVID-19 pandemic. Vol. 20, The Lancet Infectious Diseases. Lancet Publishing Group; 2020. p. e261–7.
35. The Lancet. COVID-19: protecting health-care workers. Vol. 395, The Lancet. Lancet Publishing Group; 2020. p. 922.
36. COVID-19 Prioritization of Diagnostic Testing. https://www.idsociety.org/globalassets/idsa/public-health/covid-19-prioritization-of-dx-testing.pdf. Accessed March 19, 2021.
37. The Importance of COVID-19 Vaccination for Healthcare Personnel | CDC [Internet]. [cited 2021 Mar 19]. Available from: https://www.cdc.gov/coronavirus/2019-ncov/vaccines/recommendations/hcp.html
38. Black JRM, Bailey C, Przewrocka J, Dijkstra KK, Swanton C. COVID-19: the case for health-care worker screening to prevent hospital transmission. The Lancet. 2020;395(10234):1418–20.
39. Rivett L, Sridhar S, Sparkes D, Routledge M, Jones NK, Forrest S, et al. Screening of healthcare workers for SARS-CoV-2 highlights the role of asymptomatic carriage in COVID-19 transmission. eLife. 2020;9:1–20.
40. Shields AM, Faustini SE, Perez-Toledo M, Jossi S, Aldera E, Allen JD, et al. SARS-CoV-2 seroconversion in health care workers. medRxiv. medRxiv; 2020. p. 2020.05.18.20105197.
41. Bai Y, Yao L, Wei T, Tian F, Jin DY, Chen L, et al. Presumed Asymptomatic Carrier Transmission of COVID-19. Vol. 323, JAMA - Journal of the American Medical Association. American Medical Association; 2020. p. 1406–7.

For peer review only

For peer review only - http://bmjopen.bmj.com/site/about/guidelines.xhtml

Figure 1

Participants' flowchart in the seroprevalence survey, Catalan Institute of Oncology. 21st May-26th June 2020; Spain.

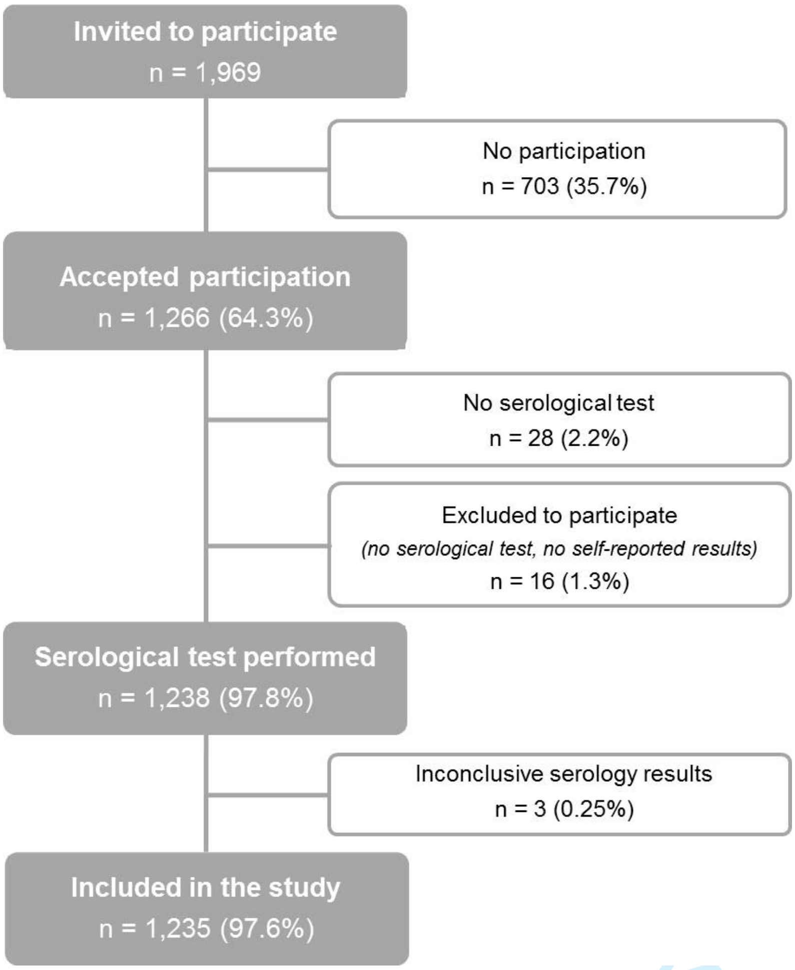

Supplementary Table 1. Clinical characteristics associated with SARS-CoV-2 positive serology among those who report rRT-PCR previous to study serology (n=469).

	Total participants n (%)	SARS-CoV-2 seroprevalence n (%)	Prevalence (95%CI)	p-value[1]	Adjusted PR (95% CI)[2]
**Reported rRT-PCR previous to serology**	469 (38.0)	86 (78.2)	18.34 (15.08-22.11)		
**Result of previous rRT-PCR**					
Negative	397 (84.6)	27 (31.0)	6.80 (4.70-9.74)		REF
Positive	72 (15.4)	59 (68.6)	81.94 (71.31-89.23)	<0.001	12.15 (7.54-19.57)
**Number of symptoms***(mean, standard deviation)*	1.65 (2.10)	3.08 (2.61)		<0.001	
None	217 (46.3)	21 (24.0)	9.68 (6.39-14.4)		REF
One	61 (13)	7 (8.1)	11.48 (5.56-22.21)		1.13 (0.48-2.67)
2-3	109 (23.2)	22 (25.6)	20.18 (13.66-28.78)		2.03 (1.10-3.73)
≥4	81 (17.3)	35 (40.7)	43.21 (32.87-54.18)	<0.001	4.33 (2.48-7.59)
p-trend (among exposed)					*<0.001*
**Reporting COVID-19 compatible symptoms when rRT-PCR was performed**					
No	217 (46.3)	21 (24.0)	9.68 (6.39-14.4)		REF
Yes	251 (53.5)	64 (74.4)	25.5 (20.48-31.27)	<0.001	2.49 (1.51-4.10)
**COVID-19 symptoms**					
Headache	126 (26.9)	36 (41.9)	28.57 (21.35-37.08)	<0.001	1.87 (1.20-2.93)
Cough	119 (25.4)	37 (43.0)	31.09 (23.42-39.97)	<0.001	2.25 (1.44-3.52)
Asthenia	110 (23.5)	36 (41.9)	32.73 (24.6-42.04)	<0.001	2.38 (1.53-3.72)
Arthromyalgia	80 (17.1)	57 (66.0)	36.25 (26.47-47.31)	<0.001	2.32 (1.47-3.67)
Low-grade fever (37.3ºC-38ºC)	73 (15.6)	26 (30.2)	35.62 (25.5-47.21)	<0.001	2.71 (1.67-4.39)
Odynophagia	64 (13.6)	14 (16.3)	21.88 (13.39-33.65)	0.40	1.18 (0.65-2.13)
Diarrhoea	58 (12.4)	16 (18.6)	27.59 (17.62-40.43)	0.05	1.47 (0.83-2.60)
Anosmia	42 (9)	33 (38.4)	78.57 (63.65-88.48)	<0.001	6.09 (3.86-9.60)
Dyspnoea	40 (8.5)	11 (12.8)	27.50 (15.91-43.2)	0.12	1.56 (0.81-3.00)
Fever (>38ºC)	28 (6)	15 (17.4)	53.57 (35.4-70.84)	<0.001	3.06 (1.71-5.46)
Nausea / vomiting	17 (3.6)	6 (7)	35.29 (16.75-59.66)	0.07	1.86 (0.80-4.36)
Skin lesions	8 (1.7)	1 (1.2)	12.50 (1.72-53.86)	0.66	0.74 (0.10-5.38)
Pneumonia	3 (0.6)	2 (2.3)	66.67 (15.27-95.69)	0.03	2.99 (0.71-12.63)
Myoclonus	2 (0.4)	0		0.50	

Numbers do not always sum up the total due to some missing values (none of the categories present more than 5% of missing values).
PR: Prevalence Ratio, CI: Confidence Interval. [1] Chi-squared test for categorical variables (Fisher's exact test corrected for continuity) and median test for continuous variables.
[2] Adjusted for sex, age (continuous), ICO center, care staff, telework and cohabitants.

STROBE 2007 (v4) Statement—Checklist of items that should be included in reports of *cross-sectional studies*

Section/Topic	Item #	Recommendation	Reported on page #
**Title and abstract**	1	(*a*) Indicate the study's design with a commonly used term in the title or the abstract	3
		(*b*) Provide in the abstract an informative and balanced summary of what was done and what was found	3
**Introduction**			
Background/rationale	2	Explain the scientific background and rationale for the investigation being reported	5
Objectives	3	State specific objectives, including any prespecified hypotheses	5
**Methods**			
Study design	4	Present key elements of study design early in the paper	5
Setting	5	Describe the setting, locations, and relevant dates, including periods of recruitment, exposure, follow-up, and data collection	5
Participants	6	(*a*) Give the eligibility criteria, and the sources and methods of selection of participants	5
Variables	7	Clearly define all outcomes, exposures, predictors, potential confounders, and effect modifiers. Give diagnostic criteria, if applicable	7
Data sources/ measurement	8*	For each variable of interest, give sources of data and details of methods of assessment (measurement). Describe comparability of assessment methods if there is more than one group	7
Bias	9	Describe any efforts to address potential sources of bias	
Study size	10	Explain how the study size was arrived at	5
Quantitative variables	11	Explain how quantitative variables were handled in the analyses. If applicable, describe which groupings were chosen and why	7
Statistical methods	12	(*a*) Describe all statistical methods, including those used to control for confounding	8
		(*b*) Describe any methods used to examine subgroups and interactions	8
		(*c*) Explain how missing data were addressed	8
		(*d*) If applicable, describe analytical methods taking account of sampling strategy	NA
		(*e*) Describe any sensitivity analyses	NA
**Results**			

For peer review only

Participants	13*	(a) Report numbers of individuals at each stage of study—eg numbers potentially eligible, examined for eligibility, confirmed eligible, included in the study, completing follow-up, and analysed	9
		(b) Give reasons for non-participation at each stage	9
		(c) Consider use of a flow diagram	6
Descriptive data	14*	(a) Give characteristics of study participants (eg demographic, clinical, social) and information on exposures and potential confounders	9
		(b) Indicate number of participants with missing data for each variable of interest	9
Outcome data	15*	Report numbers of outcome events or summary measures	12
Main results	16	(*a*) Give unadjusted estimates and, if applicable, confounder-adjusted estimates and their precision (eg, 95% confidence interval). Make clear which confounders were adjusted for and why they were included	12
		(*b*) Report category boundaries when continuous variables were categorized	9
		(*c*) If relevant, consider translating estimates of relative risk into absolute risk for a meaningful time period	
Other analyses	17	Report other analyses done—eg analyses of subgroups and interactions, and sensitivity analyses	15
**Discussion**			
Key results	18	Summarise key results with reference to study objectives	17
Limitations	19	Discuss limitations of the study, taking into account sources of potential bias or imprecision. Discuss both direction and magnitude of any potential bias	21
Interpretation	20	Give a cautious overall interpretation of results considering objectives, limitations, multiplicity of analyses, results from similar studies, and other relevant evidence	17
Generalisability	21	Discuss the generalisability (external validity) of the study results	21
**Other information**			
Funding	22	Give the source of funding and the role of the funders for the present study and, if applicable, for the original study on which the present article is based	22

*Give information separately for cases and controls in case-control studies and, if applicable, for exposed and unexposed groups in cohort and cross-sectional studies.

Note: An Explanation and Elaboration article discusses each checklist item and gives methodological background and published examples of transparent reporting. The STROBE checklist is best used in conjunction with this article (freely available on the Web sites of PLoS Medicine at http://www.plosmedicine.org/, Annals of Internal Medicine at http://www.annals.org/, and Epidemiology at http://www.epidem.com/). Information on the STROBE Initiative is available at www.strobe-statement.org.

For peer review only

COVID-19 seroprevalence among workers of a Comprehensive Cancer Center in Catalonia, Spain.

Journal:	*BMJ Open*
Manuscript ID	bmjopen-2021-056637.R1
Article Type:	Original research
Date Submitted by the Author:	15-Feb-2022
Complete List of Authors:	Peremiquel-Trillas , Paul; Catalan Institute of Oncology, Tobacco Control Unit<br>Saura-Lazaro, Anna; Catalan Institute of Oncology, Tobacco Control Unit<br>Benavente Moreno, Yolanda; Institut Català d'Oncologia<br>Casabonne, Delphine; Catalan Institute of Oncology<br>Loureiro, Eva; Catalan Institute of Oncology<br>Cabrera, Sandra; Catalan Institute of Oncology<br>Duran, Angela; Catalan Institute of Oncology<br>Garrote, Lidia; Catalan Institute of Oncology<br>Brao, Immaculada; Catalan Institute of Oncology<br>Trelis, Jordi; Catalan Institute of Oncology<br>Galán, Maica; Catalan Institute of Oncology<br>Soler, Francesc; Catalan Institute of Oncology<br>Julia, Joaquim; Catalan Institute of Oncology<br>Cortasa, Dolça; Catalan Institute of Oncology<br>Domínguez, Maria Ángeles; Bellvitge University Hospital<br>Albasanz-Puig, Adaia; Catalan Institute of Oncology<br>Gudiol, Carlota; Catalan Institute of Oncology<br>Ramírez-Tarruella, Dolors; Catalan Institute of Oncology<br>Muniesa, Joan; Catalan Institute of Oncology<br>Rivas, Juan; Catalan Institute of Oncology<br>Muñoz-Montplet, Carles; Catalan Institute of Oncology<br>Sedano, Ana; Catalan Institute of Oncology<br>Plans, Àngel; Catalan Institute of Oncology<br>Calvo-Cerrada, Beatriz; Catalan Institute of Oncology<br>Calle, Candela; Catalan Institute of Oncology<br>Clopés, Ana; Catalan Institute of Oncology<br>Carnicer-Pont, Dolors; Hospital Duran i Reynals,<br>Alemany, L.; Institut Catala d' Oncologia,<br>Fernandez, Esteve; Catalan Institute of Oncology, Tobacco Control Unit
**Primary Subject Heading**:	Epidemiology
Secondary Subject Heading:	Infectious diseases, Oncology
Keywords:	Adult oncology < ONCOLOGY, Epidemiology < INFECTIOUS DISEASES, COVID-19, Epidemiology < ONCOLOGY

SCHOLARONE™
Manuscripts

I, the Submitting Author has the right to grant and does grant on behalf of all authors of the Work (as defined in the below author licence), an exclusive licence and/or a non-exclusive licence for contributions from authors who are: i) UK Crown employees; ii) where BMJ has agreed a CC-BY licence shall apply, and/or iii) in accordance with the terms applicable for US Federal Government officers or employees acting as part of their official duties; on a worldwide, perpetual, irrevocable, royalty-free basis to BMJ Publishing Group Ltd ("BMJ") its licensees and where the relevant Journal is co-owned by BMJ to the co-owners of the Journal, to publish the Work in this journal and any other BMJ products and to exploit all rights, as set out in our licence.

The Submitting Author accepts and understands that any supply made under these terms is made by BMJ to the Submitting Author unless you are acting as an employee on behalf of your employer or a postgraduate student of an affiliated institution which is paying any applicable article publishing charge ("APC") for Open Access articles. Where the Submitting Author wishes to make the Work available on an Open Access basis (and intends to pay the relevant APC), the terms of reuse of such Open Access shall be governed by a Creative Commons licence – details of these licences and which Creative Commons licence will apply to this Work are set out in our licence referred to above.

Other than as permitted in any relevant BMJ Author's Self Archiving Policies, I confirm this Work has not been accepted for publication elsewhere, is not being considered for publication elsewhere and does not duplicate material already published. I confirm all authors consent to publication of this Work and authorise the granting of this licence.

For peer review only

COVID-19 among workers of a Comprehensive Cancer Center between first and second epidemic waves (2020): a seroprevalence study in Catalonia, Spain.

Paula Peremiquel-Trillas, Anna Saura-Lázaro*, Yolanda Benavente-Moreno, Delphine Casabonne, Eva Loureiro, Sandra Cabrera, Angela Durán, Lidia Garrote, Inmaculada Brao, Jordi Trelis, Maica Galán, Francesc Soler, Joaquim Julià, Dolça Cortasa, M. Angeles Domínguez, Adaia Albasanz-Puig, Carlota Gudiol, Dolors Ramírez, Joan Muniesa, Juan Pedro Rivas, Carles Muñoz-Montplet, Ana Sedano, Àngel Plans, Beatriz Calvo-Cerrada, Candela Calle, Ana Clopés, Dolors Carnicer-Pont†, Laia Alemany†, Esteve Fernández†*

*Contributed equally

†Joint senior authors

Authors' affiliations

PPT Cancer Epidemiology Research Programme, Cancer Epidemiology and Prevention Department, Institut Català d'Oncologia (ICO), L'Hospitalet de Llobregat, Spain; Epidemiology and Public Health Programme, Institut d'Investigació Biomèdica de Bellvitge (IDIBELL) , L'Hospitalet de Llobregat, Spain; CIBER of Epidemiology and Public Health (CIBERESP), Madrid, Spain; School of Medicine and Clinical Sciences, Universitat de Barcelona, Barcelona, Spain. @PeremiquelPaula. ORCID: 0000-0002-8636-1725

ASL Cancer Epidemiology Research Programme, Cancer Epidemiology and Prevention Department, Institut Català d'Oncologia (ICO), L'Hospitalet de Llobregat, Spain; Epidemiology and Public Health Programme, Institut d'Investigació Biomèdica de Bellvitge (IDIBELL) , L'Hospitalet de Llobregat, Spain. 0000-0001-9742-2725

YBM Cancer Epidemiology Research Programme, Cancer Epidemiology and Prevention Department, Institut Català d'Oncologia (ICO) , L'Hospitalet de Llobregat, Spain; Epidemiology and Public Health Programme, Institut d'Investigació Biomèdica de Bellvitge (IDIBELL) , L'Hospitalet de Llobregat, Spain; CIBER of Epidemiology and Public Health (CIBERESP), Madrid, Spain. ORCID: 0000-0003-1422-4614

DC Cancer Epidemiology Research Programme, Cancer Epidemiology and Prevention Department, Institut Català d'Oncologia (ICO) , L'Hospitalet de Llobregat, Spain; Epidemiology and Public Health Programme, Institut d'Investigació Biomèdica de Bellvitge (IDIBELL) , L'Hospitalet de Llobregat, Spain; CIBER of Epidemiology and Public Health (CIBERESP), Madrid, Spain. ORCID: 0000-0002-7874-3707

EL Computer Science Services, Technology & Physics, Institut Català d'Oncologia (ICO) , L'Hospitalet de Llobregat, Spain; Computational Science and Artificial Intelligence, Faculty of Computer Science of Coruña, University of Coruña (UDC), Coruña, Spain.

SC Research Nursing Department, Institut Català d'Oncologia (ICO), L'Hospitalet de Llobregat, Spain. @scabrerajaime. ORCID: 0000-0003-3013-8812

AD Nursing Department, Institut Català d'Oncologia (ICO), L'Hospitalet de Llobregat, Spain.

LG Nursing Department, Institut Català d'Oncologia – Badalona (ICO), Badalona, Spain.

IB Nursing Department, Institut Català d'Oncologia – Girona (ICO), Girona, Spain.

JT Palliative Care Department and Medical Director, Institut Català d'Oncologia (ICO), L'Hospitalet de Llobregat, Spain. @JordiTrelis.

MG Esofagogastric Tumours Functional Unit and Medical Director, Institut Català d'Oncologia (ICO), L'Hospitalet de Llobregat, Spain.

FS Pharmacy Service and Medical Director, Institut Català d'Oncologia – Girona (ICO), Girona, Spain.

JJT Palliative Care Department and Medical Director. Institut Català d'Oncologia (ICO) Badalona, Badalona, Spain. @drjjulia. ORCID: 0000-0002-1462-3167

DC Medical Director, Institut Català d'Oncologia -Tarragona/Terres de l'Ebre, Tarragona (ICO).

MADL Microbiology Department. Hospital Universitari de Bellvitge; Infectious Diseases Programme, Institut d'Investigació Biomèdica de Bellvitge (IDIBELL); Department of Pathology Experimental Therapeutics, Universitat de Barcelona, Barcelona, Spain.

For peer review only - http://bmjopen.bmj.com/site/about/guidelines.xhtml

AAP	Infectious Disease Department, Hospital Universitari de Bellvitge, Institut Català d'Oncologia (ICO), L'Hospitalet de Llobregat, Spain. CIBER of Infectious Diseases (CIBERINFEC), Madrid, Spain. ORCID: 0000-0001-9852-5574.
CG	Infectious Disease Department, Hospital Universitari de Bellvitge, Institut Català d'Oncologia (ICO), L'Hospitalet de Llobregat, Spain. CIBER of Infectious Diseases (CIBERINFEC), Madrid, Spain. @Carlotasway3. ORCID: 0000-0003-3095-4422
DR	Preventive Medicine Unit, Institut Català d'Oncologia (ICO), L'Hospitalet de Llobregat, Spain.
JM	Computer Science Services, Technology & Physics Department, Institut Català d'Oncologia (ICO), L'Hospitalet de Llobregat, Spain.
JPR	Computer Science Services, Technology & Physics Department, Institut Català d'Oncologia (ICO), L'Hospitalet de Llobregat, Spain.
CMM	Technology and Physics Department, Institut Català d'Oncologia (ICO), L'Hospitalet de Llobregat, Spain; Medical Physics and Radiation Protection Department, Institut Català d'Oncologia (ICO) – Girona, Girona, Spain; Department of Medical Sciences, Universitat de Girona, Girona, Spain. @CMunozMontplet. ORCID: 0000-0002-7324-8889.
AS	Human Resources Department, Institut Català d'Oncologia (ICO), L'Hospitalet de Llobregat, Spain.
AC	Scientific Direction, Institut Català d'Oncologia (ICO), L'Hospitalet de Llobregat, Spain. ORCID: 0000-0002-1857-362X.
BCC	Occupational Health Unit, Institut Català d'Oncologia (ICO), L'Hospitalet de Llobregat, Spain.
CC	General Direction, Institut Català d'Oncologia (ICO), L'Hospitalet de Llobregat, Spain. @CandelaCalle.
AP	Occupational Health Unit, Institut Català d'Oncologia (ICO), L'Hospitalet de Llobregat, Spain. ORCID: 0000-0003-3199-0361.
DCP	Cancer Prevention and Control Programme, Cancer Epidemiology and Prevention Department, Catalan Institute of Oncology (ICO) , L'Hospitalet de Llobregat, Spain; Epidemiology and Public Health Programme, Institut d'Investigació Biomèdica de Bellvitge (IDIBELL), L'Hospitalet de Llobregat, Spain. ; CIBER of Respiratory Diseases (CIBERES). ORCID: 0000-0002-3475-8704.
LA	Cancer Epidemiology Research Programme, Cancer Epidemiology and Prevention Department, Institut Català d'Oncologia (ICO) , L'Hospitalet de Llobregat, Spain; Epidemiology and Public Health Programme, Institut d'Investigació Biomèdica de Bellvitge (IDIBELL), L'Hospitalet de Llobregat, Spain; CIBER of Epidemiology and Public Health (CIBERESP), Madrid, Spain. ORCID: 0000-0003-0945-6015.
EF	Cancer Prevention and Control Programme, Cancer Epidemiology and Prevention Department, Institut Català d'Oncologia (ICO) , L'Hospitalet de Llobregat, Spain; Epidemiology and Public Health Programme, Institut d'Investigació Biomèdica de Bellvitge (IDIBELL), L'Hospitalet de Llobregat, Spain; School of Medicine and Clinical Sciences, Universitat de Barcelona, Barcelona, Spain; CIBER of Respiratory Diseases (CIBERES). @StvFdz. ORCID: 0000-0003-4239-723X.

Word counts.

Abstract: 296. Main text: 3,309. One figure, three tables, supplementary information.

Correspondence to: Prof. Esteve Fernández, MD, MPH, PhD. Tobacco Control Unit, Cancer Prevention and Control Programme, Institut Català d'Oncologia (ICO). Avinguda de la Granvia de l'Hospitalet, 199-203, 08908 L'Hospitalet de Llobregat, Barcelona, Spain. Phone: +34 93 2607345. Twitter: @StvFdz E-mail: efernandez@iconcologia.net (Prof. E. Fernández).

For peer review only

ABSTRACT

Objectives Cancer patients are at higher risk for severe COVID-19 infection. COVID-19 surveillance of workers in oncological centres is crucial to assess infection burden and prevent transmission. We estimate the SARS-CoV-2 seroprevalence among health care workers (HCW) of a comprehensive cancer centre in Catalonia, Spain, and analyse its association with sociodemographic characteristics, exposure factors and behaviours.

Design Cross-sectional study (21st May – 26th June 2020).

Setting A comprehensive cancer centre (Institut Català d'Oncologia) in Catalonia, Spain.

Participants All HCW (*N*=1,969) were invited to complete an online self-administered epidemiological survey and provide a blood sample for SARS-CoV-2 antibodies detection.

Primary outcome measure Prevalence (%) and 95% confidence intervals (CI) of seropositivity together with adjusted prevalence ratios (aPR) and 95%CI were estimated.

Results A total of 1,266 HCW filled the survey (participation rate: 64.0%) and 1,238 underwent serological testing (97.8%). The median age was 43.7 years (p25-p75: 34.8-51.0 years), 76.0% were female, 52.0% were nursing or medical staff, and 79.0% worked on-site during the pandemic period. SARS-CoV-2 seroprevalence was 8.9% (95%CI: 7.44-10.63), with no differences by age and sex. No significant differences in terms of seroprevalence were observed between onsite workers and teleworkers. Seropositivity was associated with living with a person with COVID-19 (aPR: 3.86, 95%CI: 2.49-5.98). Among on-site workers, seropositive participants were twofold more likely to be nursing or medical staff. Nursing and medical staff working in a COVID-19 area showed a higher seroprevalence than other staff (aPR: 2.45, 95% CI: 1.08-5.52).

Conclusions At the end of the first wave of the pandemic in Spain, SARS-CoV-2 seroprevalence among Institut Català d'Oncologia HCW was lower than the reported in other Spanish hospitals. The main risk factors were sharing household with infected people and contact with COVID-19 patients and colleagues. Strengthening preventive measures and health education among HCW is fundamental.

Keywords SARS-CoV-2; COVID-19; seroprevalence; antibody; health care workers; epidemiology.

For peer review only

ARTICLE SUMMARY

Strenghts and limitations

- Seroepidemiological study with a large sample size settled in a monographic oncological health centre.
- Questionnaire completeness was very high, with no variables presenting more than 5% of missing values.
- Recall bias is possible as the data for the correlates of SARS-CoV-2 infection rely on a self-administered questionnaire.
- The accomplishment of preventive measures might be overestimated: response and perception biases must be considered, as well as complacency bias.
- Answers reported in the questionnaire could be influenced by the participants' knowledge regarding their COVID status.

INTRODUCTION

Frontline health care workers (HCW) dealing with COVID-19 have higher exposure to SARS-CoV-2 than the general population (1), and they can contribute to the spread of COVID-19 as per their exposure to vulnerable patients. Since the beginning of the pandemic, several studies have been published on SARS-CoV-2 infections prevalence in HCW, although with diverse results. A meta-analysis of 49 studies, including 127,480 health care workers, showed that the overall seroprevalence of SARS-CoV-2 antibodies in the European region was 8.5% (2). HCW in Spain have been highly affected: a total amount of 154,636 cases among HCW were already officially notified by December 2, 2021 at the onset of the sixth pandemic wave (4).

Cancer patients are vulnerable, presenting a high risk for COVID-19 infection and more severe outcomes due to their immunosuppression status (5). The pandemic has presented unprecedented professional and personal challenges for the oncology community (6). Data are lacking on the seroprevalence of SARS-CoV-2 among HCW in oncological centres, and small sample sizes limit the few published studies. The present study aims to estimate the seroprevalence of SARS-CoV-2 and associated sociodemographic and behavioural risk factors among workers of the Catalan Institute of Oncology (ICO), a Comprehensive Cancer Centre comprised of four hospitals in Catalonia (Spain), covering around 40% of the adult population in Catalonia (7).

For peer review only - http://bmjopen.bmj.com/site/about/guidelines.xhtml

PARTICIPANTS AND METHODS

Study design and setting

A cross-sectional study including blood sample collection and a self-administered questionnaire was conducted between 21st May and 26th June 2020 in the four ICO centres (L'Hospitalet de Llobregat, Badalona, Tarragona/Terres de l'Ebre and Girona).

The study population were HCW delivering care and services to patients (directly or indirectly) and support staff, including those who do not deliver care but work in other tasks within the hospital. A total of 1,969 employees of ICO were invited to participate in the study through an email that allowed access to the study information. The inclusion criteria were: a) to be an active worker during the epidemic period, (1st February - 26th June 2020) and b) to be aged ≥18years. The participants filled in an online epidemiological questionnaire and were scheduled for serology testing by the Occupational Health Department. 1,266 HCW filled in the online epidemiological questionnaire (participation rate: 64.3%) and 1,238 of them (97.8%) underwent a serology test. Three participants with inconclusive serological results were excluded. The final analysis included 1,235 participants (**Figure 1**).

Figure 1 about here

Epidemiological questionnaire and study variables

An epidemiological questionnaire was programmed online to collect information regarding sociodemographic characteristics, working information, compliance of personal protective equipment (PPE) measures at work, at home and history of previous COVID-19 infection (**Supplemental Material**). The questionnaire was developed based on previous epidemiological studies conducted within the ICO centres, and a modified version was used in another seroprevalence study performed among university personnel of the University of Barclona (8).

Sociodemographic characteristics included information on age and sex, ICO centre of recruitment, presence of comorbidities, smoking history, pregnancy and cohabitants.

For peer review only - http://bmjopen.bmj.com/site/about/guidelines.xhtml

Work-related conditions included the professional category, teleworking status, type of shift, working on a COVID-19 area, contact with COVID-19 cases, contact with biological samples and reporting to be exposed to COVID-19.

Concerning PPE measures at work, participants were asked about feeling protected with PPE and compliance with PPE measures. Regarding the application of preventive measures outside the working setting, participants were asked if they got a shower after leaving the workplace or when arriving home, if they changed clothes after work or upon home arrival, as well as about hand washing and use of face mask when shopping. Information about COVID-19 cases and protective measures were also collected among those participants reporting cohabitants. Participants were also asked about the type of transport used to go to work.

Participants were asked about a previous diagnosis of COVID-19 performed by rRT-PCR or serology test and date of diagnosis, as well as reporting COVID-19 compatible symptoms, and the type of symptoms.

SARS-CoV-2 laboratory testing

Serum samples from participants at L'Hospitalet, Girona and Tarragona/Terres de l'Ebre were studied at the Microbiology Department of Hospital de Bellvitge and samples from health-care workers at ICO Badalona were analysed at the MetroNord Regional Clinical Laboratory, using the same procedures and techniques in both laboratories. Detection of SARS-CoV-2 antibodies was carried out using the quantitative SARS-CoV-2 S1/S2 IgG LIAISON® test (DiaSorin, Vercelli, Italy) on the LIAISON XL platform, following the manufacturer's instructions. This test discriminates among negative (<12AU/mL; with 3.8 as IgG detection limit), equivocal (12.0–15.0AU/mL) and positive (>15.0AU/mL) subjects. In those cases in which a) IgG anti S1/S2 quantification was higher than the limit of detection (i.e.>3.8AU/mL) but did not reach the limit of discrimination (i.e.<15AU/mL) and/or b) when the HCW answered the questionnaire saying that he or she had been diagnosed of COVID-19 but IgG anti S1/S2 where lower than 15 AU/ml, an additional serological study was performed using a different antigen (N) as a target. In this case, a SARS-CoV-2 IgG test (Abbott Diagnostics, Sligo, Ireland) was run on an Architect i2000 platform. This test discriminates among negative (<1.4Index (S/C)) and positive (≥1.4Index (S/C)) subjects.

For peer review only

137 **Case definition**

138 A seropositive case of SARS-CoV-2 was defined as seropositivity to IgG independently of previous self-reported results.

140 **Patient and Public Involvement**

141 No patient was involved in the study.

142 **Statistical analysis**

143 Crude global and by subgroups SARS-CoV-2 seroprevalence and 95% confidence intervals (CI) were calculated. Differences in the distribution of study variables between seropositive and seronegative participants were assessed using chi-squared test for categorical variables, and parametric or non-parametric tests were performed for normal and non-normal continuous variables, respectively. Prevalence ratios (PR) and 95% confidence intervals (CI) were estimated using Poisson regression models with robust variance (9). Prevalence ratios were adjusted (aPR) for statistically significant variables in the bivariate analysis and those considered relevant for the study design. Thus, adjusted models included sex, ICO centre of recruitment, age, type of HCW, teleworking and cohabitants. Linear trends for variables with ordinal categories was based in the likelihood ratio test of the model with the ordinal variable as a continuous one. *P*-values were based on 2-sided hypothesis tests and considered significant at $p<0.05$. All analyses were conducted using Stata version 16.0 (StataCorp LP, College Station, Texas).

156 **Ethical considerations**

157 The present study was approved by the Hospital Universitari de Bellvitge Ethics Committee (PR205/20). The study follows the Helsinki Declaration and subsequent amendments, and Spanish data confidentiality laws (General data protection regulation Organic Law 3/2018, EU General data protection Regulation 2016/679 and Law 14/2007 for biomedical research). All participants signed an informed consent form after receiving information of the study and prior to obtaining biological samples. The biological material obtained was kept at ICO and processed under the appropriate measures to preserve the confidentiality of the results and data.

For peer review only

For peer review only - http://bmjopen.bmj.com/site/about/guidelines.xhtml

RESULTS

A total of 1,235 HCW with serological results **(Figure 1)** were included in the analysis: 76.0% were female, the median age was 43.7 years (p25-p75: 34.8-51.0 years), 52.2% were nursing or medical staff, and 18.6% of the participants teleworked full-time during the study period (**Table 1**). Up to 14.7% of the participants reported at least one comorbidity. Regarding smoking habits, 16.0% were current smokers, and 28.2% reported to be former smokers (**Table 1**). Seven women were pregnant, and none of them showed seropositivity.

The overall crude SARS-CoV-2 seroprevalence was 8.9% (95%CI: 7.44-10.63), with no statistically significant differences by neither age group nor sex, and the seroprevalence for nursing and medical staff was 11.6% (95%CI: 9.37-14.34). After fully adjustment, the main determinants of higher seroprevalence included working at ICO Girona compared to workers at ICO L'Hospitalet (aPR: 1.52, 95%CI: 0.97-2.38), and nursing or medical staff compared to other groups (aPR: 2.04, 95%CI: 1.33-3.14) (**Table 1**).

For peer review only

For peer review only - http://bmjopen.bmj.com/site/about/guidelines.xhtml

Table 1. Sociodemographic characteristics associated with SARS-CoV-2 positive serology among study participants (*N*=1,235).

	Total participants *n* (%)	SARS-CoV-2 seroprevalence *n* (%)	Prevalence (95%CI)	*p-value* [6]	aPR (95% CI) [7]
**Study participants**	1,235	110	8.91 (7.44-10.63)		
**Sex**					
Male	291 (23.6)	27 (24.5)	9.28 (6.44-13.20)		REF
Female	939 (76.0)	83 (75.5)	8.84 (7.18-10.83)	*0.82*	0.82 (0.53-1.28)
**Age [median, (p25-p75)]**	43.7 (34.8-51.0)	42.8 (32.0-50.1)		*0.62*	0.99 (0.97-1.01)
<35 years	313 (25.3)	33 (30.0)	10.54 (7.59-14.46)		REF
35-49 years	566 (45.8)	47 (42.7)	8.30 (6.29-10.88)		0.85 (0.55-1.34)
>49 years	356 (28.8)	30 (27.3)	8.43 (5.95-11.80)	*0.5*	0.88 (0.53-1.46)
**ICO Center**					
ICO L'Hospitalet	885 (71.7)	73 (66.4)	8.25 (6.61-10.25)		REF
ICO Girona	204 (16.5)	29 (26.4)	14.22 (10.06-19.72)		1.52 (0.97-2.38)
ICO Badalona	134 (10.9)	7 (6.4)	5.22 (2.51-10.56)		0.54 (0.25-1.19)
ICO Tarragona / Terres de l'Ebre	12 (1.0)	1 (0.9)	8.33 (1.16-41.38)	*0.02*	1.07 (0.15-7.83)
**Professional category**					
Nursing staff[1]	380 (30.8)	43 (39.0)	11.32 (8.50-14.92)		REF
Medical Staff[2]	265 (21.5)	32 (29.1)	12.08 (8.67-16.58)		1.07 (0.65-1.76)
Middle and superior technicians	285 (23.1)	14 (12.7)	4.91 (2.93-8.13)		0.41 (0.22-0.77)
Service staff[3]	114 (9.2)	2 (1.8)	7.02 (3.55-13.42)		0.69 (0.31-1.54)
Porter	21 (1.7)	8 (7.3)	9.52 (2.39-31.16)		0.74 (0.17-3.24)
Administratives	129 (10.4)	8 (7.3)	6.20 (3.13-11.92)		0.54 (0.25-1.16)
Other	20 (1.6)	1 (0.9)	5.00 (0.70-28.26)	*0.03*	0.50 (0.07-3.71)
Nursing or medical staff[4]	645 (52.2)	75 (68.2)	11.63 (9.37-14.34)	*<0.001*	2.04 (1.33-3.14)
Other staff[5]	569 (46.1)	33 (30.0)	5.80 (4.15-8.05)		REF
**Telework**					
Never/Occasionally	981 (79.4)	86 (78.1)	8.77 (7.15-10.71)		REF
Always	230 (18.6)	23 (20.9)	10.00 (6.72-14.63)	*0.56*	1.60 (0.98-2.59)

For peer review only

Table 1 (continued)

	Total participants	SARS-CoV-2 seroprevalence	Prevalence (95%CI)	*p-value*[7]	aPR (95% CI)[8]
**Shift work**					
Morning	545 (44.1)	49 (45.0)	8.99 (6.86-11.7)		REF
Evening	140 (11.3)	10 (9.1)	7.14 (3.88-12.77)		0.56 (0.34-0.93)
Split shift (morning-evening)	417 (33.8)	38 (34.5)	9.11 (6.7-12.28)		0.88 (0.57-1.37)
Night	88 (7.1)	10 (9.1)	11.36 (6.22-19.86)		0.95 (0.46-1.96)
Other	25 (2)	3 (2.7)	12 (3.92-31.32)	*0.83*	1.15 (0.35-3.75)
**Comorbidities**[6]					
None	1,054 (85.3)	99 (90.0)	9.39 (7.77-11.31)		REF
Yes	181 (14.7)	11 (10.0)	6.08 (3.4-10.64)	*0.15*	0.67 (0.36-1.25)
**Smoking history**					
Never	650 (52.6)	80 (72.7)	12.31 (9.99-15.07)		REF
Past	348 (28.2)	22 (20.0)	6.32 (4.20-9.42)		0.57 (0.35-0.93)
Current	198 (16.0)	8 (7.3)	4.04 (2.03-7.87)	*0.0002*	0.38 (0.18-0.79)
**Cohabitants**					
Yes	1,119 (90.6)	95 (86.0)	8.49 (6.99-10.27)		REF
No	104 (8.4)	15 (13.6)	14.42 (8.88-22.57)	*0.04*	1.48 (0.83-2.66)

Numbers do not always sum up the total due to some missing values (none of the categories present more than 5% of missing values).
aPR: adjusted Prevalence Ratio, CI: Confidence Interval, p25: 25% percentile, p75: 75% percentile.
[1] Nursing staff: nurses and nursing assistants.
[2] Medical staff: resident physicians and specialists.
[3] Service staff: security, maintenance, cleaning and kitchen.
[4] Nurses, nursing assistants, resident physicians and specialists.
[5] Middle and superior technicians, security, maintenance, cleaning, kitchen, porter, administrative, and other.
[6] Comorbidities: hypertension, obesity (BMI≥30), heart disease, liver disease, diabetes, chronic respiratory disease, renal disease, cancer, autoimmune disorders and other immunological disorders.
[7] Chi-squared test for categorical variables (Fisher's exact test corrected for continuity) and median test for continuous variables.
[8] Adjusted for sex, age (continuous), ICO centre, type of health care workers, telework and cohabitants.

For peer review only - http://bmjopen.bmj.com/site/about/guidelines.xhtml

Seroprevalence among on-site workers was 8.8% (95%CI: 7.15-10.71) (**Table 2**). Onsite workers were younger, mostly health care workers, and reported more frequently rRT-PCR previous to serology than teleworkers, but no differences were observed in sex, self-reported comorbidities, smoking history, cohabiting with COVID-19 positive case between them and teleworkers (**Supplemental Material**). Among this group (*N*=981) of professionals who never or occasionally teleworked SARS-CoV-2 seropositivity was not associated with not working in a COVID-19 area (aPR: 1.29, 95%CI: 0.81-2.06), nor being in contact with COVID-19 biological samples (aPR: 1.30, 95%CI: 0.77-2.20) nor being in contact with patients with COVID-19 (aPR: 1.09, 95%CI: 0.66-1.79) were associated with SARS-CoV-2 positivity (**Table 2**). On-site nursing or medical staff who worked in a COVID area had twofold SARS-CoV-2 seroprevalence than others who did not work in COVID area (aPR: 2.45, 95%CI: 1.08-5.52). Seropositivity was higher among those whom referred being exposed by interacting with colleagues (aPR: 3.26, 95%CI: 1.49-7.15). On-site workers who self-reported symptoms of COVID-19 were almost 10-fold more likely to be seropositive than those who did not (aPR: 9.5, 95%CI: 5.34-17.03). Most of the on-site workers were highly adherent to the recommendation of hand hygiene at work. Hand washing before eating or working, were followed by more than 97% of on-site workers, whereas around 24% of them reported not hand hygiene after working or a low frequency of handwashing during the workday. In relation to protective measures at work, 17.4% of the on-site workers did not feel protected with PPE, and 12.1% did not use PPE with confirmed or suspicious COVID-19 cases. About colleagues' behaviour, 2m safety distance from colleagues when having lunch was reported to be unfollowed by 14.1% (**Table 2**).

For peer review only - http://bmjopen.bmj.com/site/about/guidelines.xhtml

Table 2. Occupational factors associated with SARS-CoV-2 positive serology among on-site workers (*N*=981).

	Total participants *n* (%)	SARS-CoV-2 seroprevalence *n* (%)	Prevalence (95%CI)	*p-value* [2]	Adjusted PR (95% CI) [3]
**On-site workers**	981 (79.4)	86 (78.1)	8.77 (7.15-10.71)	*0.56*	
**Type of transport to work**					
Private	751 (76.6)	66 (76.7)	8.79 (6.96-11.04)		REF
Public	154 (15.7)	15 (17.4)	9.74 (5.95-15.54)		1.32 (0.74-2.36)
Private and Public	35 (3.6)	2 (2.3)	5.71 (1.43-20.19)		0.63 (0.15-2.58)
Walking	37 (3.8)	3 (3.5)	8.11 (2.63-22.34)	*0.89*	0.57 (0.14-2.35)
**Working in a COVID-19 area**					
No	398 (40.6)	29 (33.7)	7.29 (5.11-10.29)		REF
Yes	545 (55.6)	55 (63.9)	10.09 (7.83-12.92)	*0.14*	1.29 (0.81-2.06)
**Type of and COVID area** [1]					
Non-assisting HCW & never worked in a COVID-19 area	148 (15.1)	7 (8.0)	4.73 (2.27-9.6)		REF
Non-assisting HCW & ever worked in a COVID-19 area	230 (23.4)	13 (15.1)	5.65 (3.31-9.5)		1.12 (0.44-2.82)
Assisting HCW & never worked in a COVID-19 area	244 (24.9)	22 (25.6)	9.02 (6.01-13.32)		1.81 (0.77-4.26)
Assisting HCW & ever worked in a COVID-19 area	311 (31.7)	40 (46.5)	12.86 (9.57-17.07)	*0.006*	2.45 (1.08-5.52)
p-trend					0.26
**Contact with COVID-19 cases**					
No	333 (33.9)	23 (26.7)	6.91 (4.63-10.18)		REF
Yes	536 (54.6)	57 (66.3)	10.63 (8.29-13.54)	*0.07*	1.30 (0.77-2.20)
**Contact with COVID-19 biological samples**					
No	646 (65.9)	51 (59.3)	7.89 (6.05-10.24)		REF
Yes	282 (28.7)	30 (34.9)	10.64 (7.54-14.81)	*0.17*	1.09 (0.66-1.79)
**Reporting to be exposed to COVID-19 by interacting with colleagues at work**					
No	242 (24.7)	66 (76.7)	2.89 (1.38-5.95)		REF
Yes	608 (62.0)	7 (8.1)	10.86 (8.62-13.59)	*<0.0001*	3.26 (1.49-7.15)
**Reporting COVID-19 compatible symptoms**					
No	623 (63.5)	15 (17.4)	2.41 (1.46-3.96)		REF
Yes	306 (31.2)	68 (79.1)	22.22 (17.91-27.23)	*<0.0001*	9.53 (5.34-17.03)

For peer review only - http://bmjopen.bmj.com/site/about/guidelines.xhtml

Table 2 (continued)

	Total participants	SARS-CoV-2 seroprevalence	Prevalence (95%CI)	*p-value* [2]	Adjusted PR (95% CI) [3]
**Not following protection measures at work**					
Felt protected with PPE	132 (17.4)	12 (16.9)	9.09 (5.23-15.34)	*0.83*	0.98 (0.51-1.88)
Colleagues cover themselves with their elbows when sneezing/coughing	155 (15.8)	21 (24.4)	13.55 (9.00-19.90)	*0.01*	1.70 (1.01-2.87)
2m safety distance from colleagues during lunch	127 (14.1)	12 (15.6)	9.45 (5.44-15.91)	*0.71*	1.06 (0.56-1.99)
Use of PPE with confirmed or suspicious COVID-19 patients	79 (12.1)	7 (10.45)	8.86 (4.28-17.46)	*0.63*	1.01 (0.45-2.26)
PPE removal safety	48 (7.3)	3 (4.6)	6.25 (2.03-17.68)	*0.33*	0.54 (0.17-1.74)
Personal use of mask	34 (3.5)	1 (1.2)	2.94 (0.41-18.17)	*0.21*	0.41 (0.06-2.99)
Colleagues use of surgical mask	7 (0.7)	1 (1.2)	14.29 (1.96-58.12)	*0.62*	1.68 (0.23-12.29)
**Not following hand hygiene at work**					
≤7 times during workday	233 (23.8)	15 (17.4)	6.44 (3.92-10.41)	*0.13*	0.71 (0.39-1.28)
After money, phone and other personal tools manipulation	175 (17.8)	16 (18.6)	9.14 (5.67-14.41)	*0.89*	1.00 (0.58-1.74)
Every time entering in a new workspace	102 (10.4)	5 (5.8)	4.90 (2.05-11.25)	*0.14*	0.55 (0.22-1.37)
Before working	21 (2.1)	3 (3.5)	14.29 (4.67-36.17)	*0.37*	1.72 (0.54-5.47)
After finishing the workday	17 (1.7)	1 (1.2)	5.88 (0.82-32.09)	*0.67*	0.65 (0.09-4.72)
Before eating	9 (0.9)	2 (2.3)	22.22 (5.59- 57.95)	*0.16*	2.67 (0.65-10.94)

Numbers do not always sum up the total due to some missing value (none of the categories present more than 5% of missing values).
PR: Prevalence Ratio, CI: Confidence Interval, HCW: Health Care Workers.
[1] Assisting HCW: nurses, nursing assistants, resident physicians and specialists; otherwise, classified and non-assisting HCW.
[2] Chi-squared test.
[3] Adjusted for sex, age (continuous), ICO centre, care staff, telework and cohabitants.

For peer review only

For peer review only - http://bmjopen.bmj.com/site/about/guidelines.xhtml

Concerning the correlates of seropositivity according to household factors for all participants (**Table 3**), seropositivity was associated with living with a COVID-19 positive person (aPR: 3.86, 95%CI: 2.49-5.98). Up to 17.3% of the participants did not take a shower nor change clothes upon home arrival, but the majority (99.0%) did hand hygiene. The least followed hand hygiene home practices were after money, phone and other personal tools manipulation, and after nose blowing, coughing or sneezing (23.5% and 22.7%). However, not following protection measures or hand hygiene at home were associated with a higher SARS-CoV-2 seroprevalence.

Clinical characteristics were collected for those participants (*N*=469) who reported a rRT-PCR performed previous to serology (**Supplemental material**). The majority of the patients with a positive serology and reporting a positive rRT-PCR presented compatible COVID-19 symptoms (74.4%). Among seropositive patients, the most common symptoms were arthromyalgia, cough, headache, asthenia and anosmia. Reporting a positive rRT-PCR when presenting compatible symptoms was associated with a threefold higher prevalence of seropositivity (aPR: 3.10, 95%CI: 1.78-5.31). An increased number of compatible symptoms was also associated with a higher seroprevalence (aPR: 7.4, 95%CI: 1.78-5.31, for presenting four or more symptoms compared to no symptoms).

For peer review only

Table 3. Household factors associated with SARS-CoV-2 positive serology among study participants (n=1,235).

	Total participants *n* (%)	SARS-CoV-2 seroprevalence *n* (%)	Prevalence (95%CI)	*p-value* [2]	Adjusted PR (95% CI) [3]
**Study participants**	1,235	110	8.91 (7.44-10.63)		
**Cohabitants with COVID-19**[1]					
No	894 (79.9)	52 (54.7)	5.82 (4.46-7.56)		REF
Yes	141 (12.60)	34 (35.8)	24.11 (17.76-31.86)	*<0.0001*	3.86 (2.49-5.97)
**Cohabitants cover themselves with their elbow when sneezing**					
No	158 (14.1)	18 (18.9)	11.39 (7.29-17.37)		REF
Yes	919 (82.1)	73 (76.8)	7.94 (6.36-9.88)	*0.15*	0.73 (0.43-1.22)
**Not following protection measures at home**[4]					
Use of face mask when shopping	17 (1.4)	2 (1.8)	11.76 (2.95-36.86)	*0.67*	0.98 (0.24-4.05)
Shower and clothes changing afterwork or upon home arrival	214 (17.3)	20 (18.2)	9.35 (6.11-14.05)	*0.82*	1.02 (0.62-1.69)
**Not following hand hygiene at home**[4]					
Upon arrival	12 (1)	2 (1.8)	16.67 (4.19-47.76)	*0.35*	1.59 (0.39-6.60)
Before eating	60 (4.9)	9 (8.2)	15.00 (7.99-26.4)	*0.09*	1.55 (0.77-3.12)
After money, phone and other personal tools manipulation	290 (23.5)	27 (24.6)	9.31 (6.46-13.24)	*0.71*	1.01 (0.65-1.58)
After cleaning	110 (8.9)	8 (7.3)	7.27 (3.68-13.88)	*0.53*	0.78 (0.38-1.61)
After nose blowing	280 (22.7)	25 (22.7)	8.93 (6.1-12.88)	*0.99*	0.93 (0.58-1.48)

Numbers do not always sum up the total due to some missing values (none of the categories present more than 5% of missing values).
PR: Prevalence Ratio, CI: Confidence Interval.
[1] Analyses performed among those participants who reported having cohabitants (n=1,119).
[2] Chi-squared test.
[3] Adjusted for sex, age (continuous), ICO center, care staff, telework and cohabitants.
[4] Unfollowing the measures of protection and hand hygiene recommendations.

For peer review only - http://bmjopen.bmj.com/site/about/guidelines.xhtml

DISCUSSION

Despite the impact of COVID-19 in oncological patients (10), there are scarce SARS-CoV-2 seroprevalence studies in comprehensive cancer centres with large sample sizes. The global SARS-CoV-2 seroprevalence was 8.9% during the first wave of the COVID-19 pandemic, lower than expected, owing to the presumed higher risk among HCW. Also, it was lower than the reported estimates in two studies performed among HCW in Catalonia between March-April and May 2020, showing a seroprevalence of 11.2% (11) and 10.3% (12), respectively. In all cases, the seroprevalence was higher than in the general population, estimated to be of a maximum of 7.4% in the Barcelona metropolitan area when the study was conducted (13). Seroprevalence studies interpretation must be related to the average COVID-19 prevalence at the time of blood collection. Both of the mentioned studies were carried out earlier than ours, which was performed approximately one month later (21st May-26th June 2020), and two months after the first-wave peak in Catalonia (23th March) (14). Another explanation for this lower seroprevalence in our Centre concerns the participation: all active HCW, regardless of their teleworking status during the previous months or work absenteeism, were invited to participate, and most did (64%). In contrast, García-Basteiro's (11) and Barallat's (12) studies comprised general hospitals (10,11) and primary health care centers (12) in which the incidence could be higher than in a monographic cancer centre.

Several studies regarding COVID-19 infections in HCW in Spain have been published, although showing diverse results. In a tertiary-care hospital in Mallorca, with low regional seroprevalence in the general population (<2%), the prevalence of infected HCW (n=2,210) was 2.8%(15). Varona et al. performed a cross-sectional study evaluating 6,038 employees from the healthcare system of 17 hospitals across four regions in Spain (Madrid, Catalonia, Galicia and Castilla-Leon), showing an 11% seropositivity for SARS-CoV-2 IgG (16). Finally, other studies in Madrid, reported a seroprevalence between 16.6% and 36.5% among HCW in areas with high COVID-19 prevalence (17–19). These studies revealed seroprevalence of SARS-CoV-2 IgG antibodies in HCW tend to be higher than in the general population, at variance according to regional COVID-19 incidence.

The prevalence of SARS-CoV-2 antibodies among HCW has been increasingly investigated in many other countries showing a broad range of outcomes. So far, two systematic reviews estimated an overall seroprevalence of SARS-CoV-2 antibodies of 8.7% and 8.0% among 127,480 HCW and 168,200 HCW, respectively, before vaccination started (2,20).

For peer review only - http://bmjopen.bmj.com/site/about/guidelines.xhtml

Seroprevalence was higher in studies conducted in North America (12.7%) compared with those conducted in Europe (8.5%), Africa (8.2) and Asia (4%) (2).

In Europe, seroprevalence rates among HCW in Germany, Denmark and Belgium were low (1.6%, 4.0% and 6.4%, respectively) (21–23). These studies were conducted during early stages of the epidemic, and therefore, they derived that infection was community-acquired. Also, the Belgian study, with a sample size of almost 30,000 HCW, notes that the high availability of PPE, high standards of infection prevention, and PCR screening in symptomatic staff, coupled with contact tracing and quarantine, might explain the relatively low seroprevalence (23). An study performed in Lombardy, Italy (24), one of the Italian regions most hit by the first epidemic wave, showed a seroprevalence of 7.4% (3.8-11.0%), similar to the observed in the Catalan studies (11,12). Sweden and the UK were the two European countries reporting the highest seropositivity rates among HCW: 19.1% and between 18.0% and 45.3%, respectively (25–27). In the UK, this high seroprevalence was settled in London during the week with the highest number of new cases in the city in the first wave, with around 15% seropositivity among the general population. In the USA, the prevalence of infection among HCW was 10.7%, despite high variation, as low as 1.1% in California (28) to 13.7% in New York State (29).

Despite SARS-CoV-2 seropositivity rate in oncological HCW has significant implications for oncological patients, scant research has been done. The only study published with a large sample size was in Tokyo, Japan, and it showed a very low seroprevalence of 0.67% among 1,190 HCW. It was performed at the end of the first wave in Japan, between the 3rd of August and the 30th of October 2020, so this may explain the lower seroprevalence compared with our estimation. A French study performed among 663 HCW and 1,011 cancer patients, after the end of the first wave, showed also low seroprevalence both for HCW and patients (1.8% and 1.7%, respectively) (30). Other studies that have been published were based on small sample sizes and showed very variable seroprevalence rates (22,31–35).

In our study, we found no differences in HCW seroprevalence according to sex, age and presence of comorbidities. Current or past smoking was however inversely associated to SARS-CoV-2 seroprevalence. Early studies in selected cohorts of COVID-19 patients showed a paradoxical higher risk of SARS-CoV-2 infection among non-smokers (36) whilst ever smokers showed higher risk of COVID-19 progression, including severity of the disease, Intensive Care Unit admission and death (27,28).

For peer review only - http://bmjopen.bmj.com/site/about/guidelines.xhtml

351 It is worth mentioning that, unlike most of the other published seroepidemiological studies among HCW, the present study was performed among all the HCW of the institution, regardless they did full-time telework during the study period (21.6%). No differences by telework were found, and among all study participants the main factor associated with SARS-CoV-2 seropositivity was living with a COVID-19 case, with a times higher probability, similarly to what has been described in other studies (2,20). This finding supports the importance of community dissemination of the infection also for HCWs.

Our study shows that among on-site HCW in an oncological centre, working as medical care staff (nursing, nursing assistant, resident physicians and specialists) in COVID-19 areas stood out as one of the main factors associated with developing SARS-CoV-2 antibodies. Published results regarding the possibility of in-hospital infection among HCW and transmission at work are controversial. Some studies did not find any relation between working in COVID unit or professional category with seropositivity (11,24) whereas other studies reported that seroprevalence was strongly associated with patient related-work (16,22,25,38).

Contact with colleagues at work is potentially a risky situation for transmission among HCW as well as the relaxation of protective measures at the end of the working day. In our study, the on-site HCW who reported being exposed to COVID-19 by other colleagues presented an almost four-fold probability of being seropositive. Most of the HCW declared to follow the protective measures at the workplace, and no differences in seroprevalence were found according to protective measures and hand hygiene.

Protecting HWC health is of paramount importance for reducing morbidity and mortality, reducing transmission, and maintaining the health system capacity (39). Thus international health authorities recommend screening strategies for SARS-CoV-2 infection in exposed or high-risk HCW (40) as well as massive COVID-19 vaccination (41).

Significant differences exist in SARS-CoV-2 testing between countries, and existing programmes focus on screening symptomatic rather than asymptomatic staff. Published studies point out the fact that screening should be performed regardless of the absence of typical symptoms for COVID-19 disease. It has been demonstrated that seroconversion can occur in HCW who have suffered no previous symptoms of SARS-CoV-2 infection (42,43) as asymptomatic transmission is very relevant in SARS-CoV-2 spread (44,45). Thus, the approach for mass testing of both symptomatic and asymptomatic HCW could mitigate

For peer review only - http://bmjopen.bmj.com/site/about/guidelines.xhtml

workforce depletion by unnecessary quarantine, reduce spread in atypical, mild, or asymptomatic cases; and protect patients and health-care workforce.

Among the potential limitations of the study, some recall bias is possible as the data for the correlates of SARS-CoV-2 infection rely on a self-administered questionnaire. Also, response and perception biases must be considered, as well as complacency bias. Results, especially those regarding the accomplishment of preventive measures, might be overestimated. Answers reported in the questionnaire could be influenced by the participants' knowledge regarding their COVID status. However, this study is the first seroepidemiological study with such a large sample size settled in an oncological health centre. The sufficient sample size and high response rate (64.3%) are strengths of the study, although information regarding non-participants was not collected, and we cannot disregard a potential participation bias. However, the distribution by age and sex was similar between participants and non-participants and a possible reason for no participation is that professionals from ICO-Badalona had previously participated in a HCW county seroprevalence survey (12). Also, the fact that the information of the study and the questionnaire was published online and sent by e-mail, as well as the short period of time stablished to respond to it, could have limited the participation. Questionnaire completeness was very high, with no variables presenting more than 5% of missing values.

In conclusion, SARS-CoV-2 seroprevalence among ICO HCW at the end of the first wave of the pandemic was lower than the reported in other Catalan hospitals, but higher than among the general population living in the area. Whereas the main risk factor was living with infected people, among on-site workers, contact with colleagues was associated with SARS-CoV-2 infection. Knowing the seroprevalence rate and follow-up evaluation of persistence may help hospitals to characterize the staff at risk, rationalize their placement, prioritize the use of PPE, thereby potentially reducing the risk of infection. Follow-up studies to evaluate long term durability of antibodies among HCW will be of interest, after the introduction of COVID-19 vaccination among HCW, to better promote infection control in this group. Strengthening preventive measures and health education among HCW is fundamental, especially in oncological departments and centres.

For peer review only - http://bmjopen.bmj.com/site/about/guidelines.xhtml

Contributors EF, DCP, AP, CC, AC and AS contributed to study design. SC, AD, LG, IB, JT, MG, FS, JJT, DC, AS, BC, DR and AP accrued participants and care for blood collection at ICO centres. Laboratory analyses were coordinated by MADL. The questionnaire was designed by DCP and EF, and revised by PPT, ASL, YB, DC, AP, and LA. Questionnaire's implementation was done by EL, JM, JPR, CMM. Data were analysed by YB and DC. PPT, ASL, YB, DC, LA, and EF interpreted the initial results and designed the tables. All authors contributed to interpretation of results. The first draft of the manuscript was prepared by PPT and ASL. PPT, ASL, YB, DC, LA, DC and EF were the main contributors to the writing of the manuscript. All authors assisted in manuscript review. The co-senior authors had full access to all the data in the study for interpretation and had final responsibility for manuscript generation and review, and the decision to submit for publication. EF is the guarantor.

Funding This work was supported by the Catalan Institute of Oncology. PPT is partially supported by the Instituto de Salud Carlos III (ISCIII), Government of the Kingdom of Spain, co-funded by FEDER funds/European Regional Development Fund (ERDF) - a way to build Europe (CM19/00216). The Ministry of Universities and Research, Government of Catalonia partly supports EF and DCP (2017SGR319) and PPT, AS, YBM, DC and LA (2017SGR1085). We thank CERCA Programme / Generalitat de Catalunya for institutional support.

Competing interests JJ received research funding from Kyowa Kirin and from Angelini Pharma for congress attendance. The Cancer Epidemiology Research Programme has received grants from Merck & Co., Roche, GSK, IDT, Hologic, and Seegene.

Acknowledgments The authors acknowledge all the health care workers who participated in the study as well as all the ICO staff involved in the logistics of the different aspects of the study.

Data availability statement No additional data available

For peer review only - http://bmjopen.bmj.com/site/about/guidelines.xhtml

REFERENCES

1. Ng K, Poon BH, Kiat Puar TH, Shan Quah JL, Loh WJ, Wong YJ, et al. COVID-19 and the Risk to Health Care Workers: A Case Report. Annals of internal medicine. 2020 Jun 2;172(11):766–7.
2. Galanis P, Vraka I, Fragkou D, Bilali A, Kaitelidou D. Seroprevalence of SARS-CoV-2 antibodies and associated factors in healthcare workers: a systematic review and meta-analysis. J Hosp Infect. 2021 Feb;108:120–34.
3. Informe sobre la situación de COVID-19 en personal sanitario en España. España; 2020.
4. Centro de Coordinación de Alertas y, Emergencias Sanitarias. Actualización no 517. Enfermedad por el coronavirus (COVID-19). 03.12.2021 [Internet]. [cited 2022 Feb 1]. Available from: https://www.sanidad.gob.es/profesionales/saludPublica/ccayes/alertasActual/nCov/documentos/Actualizacion_517_COVID-19.pdf
5. Dai M, Liu D, Liu M, Zhou F, Li G, Chen Z, et al. Patients with cancer appear more vulnerable to SARS-CoV-2: A multicenter study during the COVID-19 outbreak. Cancer Discovery. 2020 Jun 1;10(6):783.
6. Leung MST, Lin SG, Chow J, Harky A. COVID-19 and Oncology: Service transformation during pandemic. Cancer Medicine. 2020 Oct 18;9(19):7161–71.
7. Institut Català d'Oncologia [Internet]. [cited 2021 Mar 19]. Available from: http://ico.gencat.cat/ca/l_institut/
8. Videla S, Otero A, Martí S, Domínguez MÁ, Fabrellas N, Delgado-Hito MP, et al. Prevalence of SARS-CoV-2 Infection at the University of Barcelona during the Third COVID-19 Pandemic Wave in Spain. Int J Environ Res Public Health. 2021 Jan;18(12):6526.
9. Barros AJD, Hirakata VN. Alternatives for logistic regression in cross-sectional studies: An empirical comparison of models that directly estimate the prevalence ratio. BMC Medical Res Methodol. 2003 Oct 20;3:1–13.
10. Richards M, Anderson M, Carter P, Ebert BL, Mossialos E. The impact of the COVID-19 pandemic on cancer care. Nature Cancer. 2020 Jun;1(6):565–7.
11. Garcia-Basteiro AL, Moncunill G, Tortajada M, Vidal M, Guinovart C, Jiménez A, et al. Seroprevalence of antibodies against SARS-CoV-2 among health care workers in a large Spanish reference hospital. Nature Comm. 2020 Dec 1;11(1):1–9.
12. Barallat J, Fernández-Rivas G, -Sánchez B Q, Martinez-Caceres. Seroprevalence of SARS-CoV-2 IgG Specific Antibodies among Healthcare Workers in the Northern Metropolitan Area of Barcelona, Spain, after the first pandemic wave. PLoS One. 2020 Dec 28;15(12):e0244348.
13. Pollán M, Pérez-Gómez B, Pastor-Barriuso R, Oteo J, Hernán MA, Pérez-Olmeda M, et al. Prevalence of SARS-CoV-2 in Spain (ENE-COVID): a nationwide, population-based seroepidemiological study. The Lancet. 2020 Aug 22;396(10250):535–44.
14. COVID-19 data and indicators. Sistemes d'Informació dels Serveis d'Atenció Primària-SISAP, Institut Català de la Salut, Departament de Salut, Generalitat de Catalunya. [Internet]. 2020 [cited 2021 Mar 19]. Available from: http://eines.portalics/covid/
15. Rodriguez A, Arrizabalaga-Asenjo M, Fernandez-Baca V, Lainez MP, Al Nakeeb Z, Garcia JD, et al. Seroprevalence of SARS-CoV-2 antibody among healthcare workers in a university hospital in Mallorca, Spain, during the first wave of the COVID-19 pandemic. Int J Infect Dis. 2021 Apr;105:482–6.

For peer review only - http://bmjopen.bmj.com/site/about/guidelines.xhtml

16. Varona JF, Madurga R, Peñalver F, Abarca E, Almirall C, Cruz M, et al. Seroprevalence of SARS-CoV-2 antibodies in over 6000 healthcare workers in Spain. Int J Epidemiol. 2021 May 17;50(2):400–9.
17. Galán MI, Velasco M, Casas ML, Goyanes MJ, Rodríguez-Caravaca G, Losa JE, et al. SARS-CoV-2 Seroprevalence Among All Workers in a Teaching Hospital in Spain: Unmasking The Risk [Internet]. medRxiv; 2020 [cited 2022 Feb 2]. p. 2020.05.29.20116731. Available from: https://www.medrxiv.org/content/10.1101/2020.05.29.20116731v1
18. Pérez-García F, Pérez-Zapata A, Arcos N, De la Mata M, Ortiz M, Simón E, et al. Severe acute respiratory coronavirus virus 2 (SARS-CoV-2) infection among hospital workers in a severely affected institution in Madrid, Spain: A surveillance cross-sectional study. Infect Control Hosp Epidemiol. 2021 Jul;42(7):803–9.
19. Cantero M, Millán-Pérez R, Muñez E, Gómez-Lozano N, Anel-Pedroche J, Parra LM, et al. SARS-COV-2 seroprevalence among all healthcare workers in a tertiary hospital in Spain. Infectious Diseases. 2022 Jan 2;54(1):8–15.
20. Kayı İ, Madran B, Keske Ş, Karanfil Ö, Arribas JR, Pshenichnaya N, et al. The seroprevalence of SARS-CoV-2 antibodies among health care workers before the era of vaccination: a systematic review and meta-analysis. Clin Microbiol Infect. 2021 Sep;27(9):1242–9.
21. Korth J, Wilde B, Dolff S, Anastasiou OE, Krawczyk A, Jahn M, et al. SARS-CoV-2-specific antibody detection in healthcare workers in Germany with direct contact to COVID-19 patients. J Clin Virol. 2020 Jul 1;128:104437.
22. Iversen K, Bundgaard H, Hasselbalch RB, Kristensen JH, Nielsen PB, Pries-Heje M, et al. Risk of COVID-19 in health-care workers in Denmark: an observational cohort study. Lancet Infect Dis. 2020;20(12):1401–8.
23. Steensels D, Oris E, Coninx L, Nuyens D, Delforge ML, Vermeersch P, et al. Hospital-Wide SARS-CoV-2 Antibody Screening in 3056 Staff in a Tertiary Center in Belgium. JAMA. 2020;324(2):195–7.
24. Sotgiu G, Barassi A, Miozzo M, Saderi L, Piana A, Orfeo N, et al. SARS-CoV-2 specific serological pattern in healthcare workers of an Italian COVID-19 forefront hospital. BMC Pulmonary Med. 2020;20(1):1–6.
25. Rudberg AS, Havervall S, Månberg A, Jernbom Falk A, Aguilera K, Ng H, et al. SARS-CoV-2 exposure, symptoms and seroprevalence in healthcare workers in Sweden. Nature Comm. 2020 Dec 1;11(1):1–8.
26. Pallett SJC, Rayment M, Patel A, Fitzgerald-Smith SAM, Denny SJ, Charani E, et al. Point-of-care serological assays for delayed SARS-CoV-2 case identification among health-care workers in the UK: a prospective multicentre cohort study. Lancet Resp Med. 2020;8(9):885–94.
27. Houlihan CF, Vora N, Byrne T, Lewer D, Heaney J, Moore DA, et al. SARS-CoV-2 virus and antibodies in front-line Health Care Workers in an acute hospital in London: Preliminary results from a longitudinal study [pre-print]. Available: medhttps://www.medrxiv.org/content/10.1101/2020.06.08.20120584v1
28. Brant-Zawadzki M, Fridman D, Robinson PA, Zahn M, Chau C, German R, et al. SARS-CoV-2 antibody prevalence in health care workers: Preliminary report of a single center study. PLoS ONE. 2020 Nov 1;15.
29. Moscola J, Sembajwe G, Jarrett M, Farber B, Chang T, McGinn T, et al. Prevalence of SARS-CoV-2 Antibodies in Health Care Personnel in the New York City Area. JAMA 2020;324(9):893–5.

For peer review only

30. Ladoire S, Goussot V, Redersdorff E, Cueff A, Ballot E, Truntzer C, et al. Seroprevalence of SARS-CoV-2 among the staff and patients of a French cancer centre after first lockdown: The canSEROcov study. Eur J Cancer. 2021 May 1;148:359–70.
31. Favara DM, McAdam K, Cooke A, Bordessa-Kelly A, Budriunaite I, Bossingham S, et al. SARS-CoV-2 Infection and Antibody Seroprevalence among UK Healthcare Professionals Working with Cancer Patients during the First Wave of the COVID-19 Pandemic. Clin Oncol. 2021 Oct;33(10):667–75.
32. Epstude J, Harsch IA. Seroprevalence of COVID-19 antibodies in the cleaning and oncological staff of a municipal clinic. GMS Hyg Infect Control. 2020;15:5–9.
33. Fuereder T, Berghoff AS, Heller G, Haslacher H, Perkmann T, Strassl R, et al. SARS-CoV-2 seroprevalence in oncology healthcare professionals and patients with cancer at a tertiary care centre during the COVID-19 pandemic. ESMO Open. 2020 Sep 2;5(5):e000889.
34. Fong D, San Nicolò KO, Alber M, Mitterer M. Evaluating the longitudinal effectiveness of preventive measures against COVID-19 and seroprevalence of IgG antibodies to SARS-CoV-2 in cancer outpatients and healthcare workers. Wien Klin Wochenschr. 2021 Apr;133(7–8):359–63.
35. Marra A, Generali D, Zagami P, Cervoni V, Gandini S, Venturini S, et al. Seroconversion in patients with cancer and oncology health care workers infected by SARS-CoV-2. Ann Oncol. 2021 Jan;32(1):113–9.
36. Simons D, Shahab L, Brown J, Perski O. The association of smoking status with SARS-CoV-2 infection, hospitalisation and mortality from COVID-19: A living rapid evidence review with Bayesian meta-analyses (version 11). Qeios. 2021 Mar 2;
37. Patanavanich R, Glantz SA. Smoking is associated with COVID-19 progression: A meta-analysis. Vol. 22, Nicotine and Tobacco Research. Oxford University Press; 2020. p. 1653–6.
38. Korth J, Wilde B, Dolff S, Anastasiou OE, Krawczyk A, Jahn M, et al. SARS-CoV-2-specific antibody detection in healthcare workers in Germany with direct contact to COVID-19 patients. J Clin Virol. 2020;128:104437.
39. The Lancet. COVID-19: protecting health-care workers. COVID-19 Prioritization of Diagnostic Testing. Lancet. 2020;395: 922.
41. The Importance of COVID-19 Vaccination for Healthcare Personnel. CDC [Internet]. [cited 2021 Mar 19]. Available from: https://www.cdc.gov/coronavirus/2019-ncov/vaccines/recommendations/hcp.html
42. Black JRM, Bailey C, Przewrocka J, Dijkstra KK, Swanton C. COVID-19: the case for health-care worker screening to prevent hospital transmission. Lancet. 2020;395(10234):1418–20.
43. Rivett L, Sridhar S, Sparkes D, Routledge M, Jones NK, Forrest S, et al. Screening of healthcare workers for SARS-CoV-2 highlights the role of asymptomatic carriage in COVID-19 transmission. eLife. 2020;9:1–20.
44. Shields AM, Faustini SE, Perez-Toledo M, Jossi S, Aldera E, Allen JD, et al. SARS-CoV-2 seroprevalence and asymptomatic viral carriage in healthcare workers: a cross-sectional study. Thorax. 2020 Dec;75(12):1089-1094.
45. Bai Y, Yao L, Wei T, Tian F, Jin DY, Chen L, et al. Presumed Asymptomatic Carrier Transmission of COVID-19. JAMA. 2020;323:1406–7.

For peer review only

Figure 1

Participants' flowchart in the seroprevalence survey, Catalan Institute of Oncology. 21st May-26th June 2020; Spain.

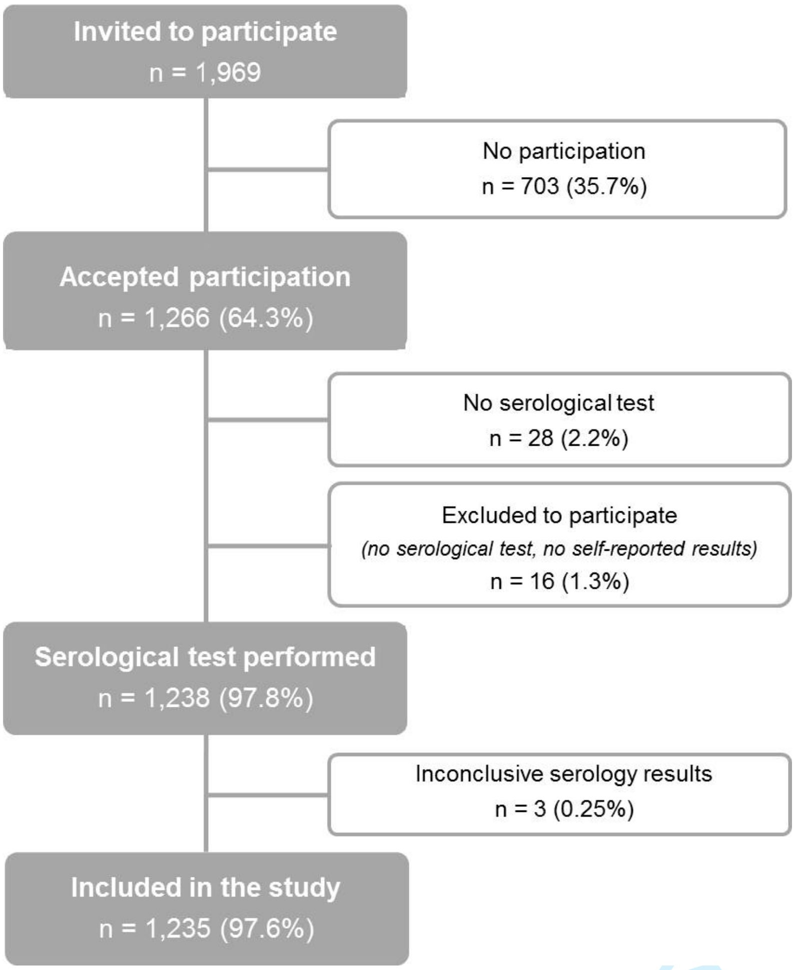

SUPPLEMENTARY MATERIAL

Accompanying the manuscript:

COVID-19 among workers of a Comprehensive Cancer Center between first and second epidemic waves (2020): a seroprevalence study in Catalonia, Spain.

Contents:

- Epidemiologic and behaviour questionnaire – ICO-Sero-COVID Study
- Supplementary Table 1. Demographic characteristics of on-site workers (always/ocassionally) and teleworkers
- Supplementary Table 2. Clinical characteristics associated with SARS-CoV-2 positive serology among those who report rRT-PCR previous to study serology (n=469).

For peer review only

Epidemiologic and behaviour questionnaire – ICO-Sero-COVID Study

I give my consent to participate in the study of seroprevalence of SARS-Cov-2 infection among ICO workers and related companies, which includes responding to an epidemiological survey with information on working conditions and obtaining a nasopharyngeal smear (to perform PCR test for virus detection) and/or to obtain blood sample by venipuncture (to perform serological tests for antibody determination and plasma cryopreservation at ICO biobank)

1 = Yes; 2 = No.

Thank you for participating in the COVID-19 seroprevalence survey among ICO workers. All information provided below will be treated confidentially, and all resulting results will be anonymized, with no individual data identifying participants.

A. Socio-demographic data.

1. Name string variable.
2. Last name1 string variable.
3. Last name2 string variable.
4. CIP string variable.
5. DNI numeric variable.
6. Sex numeric variable (1 = Woman; 2 = Man).
7. E-mail string variable.
8. ICO center or external company cathegoric variable (ICO-Gi, ICO-L'H, ICO-BDN, ICO-Tarragona-Terres Ebre, Arcasa, IDIBELL, ISS, Security, IDI, Pregecsa, Veolia).
9. Professional category numeric variable (1 = Nurse; 2 = Fac. Specialist (medicine, pharmacy, physics, psychologist); 3 = Higher Technician (Research, Predoc, Postdoc ...); 4 = MIR, FIR, PIR; 5 = Higher Technician; 6 = Porter; 7 = Administrative; 8 = Maintenance/Security; 9 = Cleaning; 10 = Restoration; 11 = Other (specify: string variable ___________________).
10. Work shift numeric variable (1 = Morning; 2 = Afternoon; 3 = Night; 4 = Other).
11. Did you telework for at least more than one day during the March to May 2020 period? numeric variable (1 = Yes; 2 = No).
12. How many days on average per week do you telework? |_ _| numeric variable (1 to 7).

B. Exposure and occupational safety measures data.

13. **Have you worked in the "COVID area" during the period comprised between March and May 2020?** numeric variable (1 = No; 2 = Yes).
14. **Since the beginning of March 2020, have you had a suspected or confirmed clinical condition as COVID-19?** numeric variable (1 = Yes; 2 = No).
15. **Since the beginning of March 2020, have you had a nasopharyngeal smears sample?** numeric variable (1 = Yes; 2 = No).
16. **Do you belong to any of the groups considered to have an increased vulnerability to COVID -19?**
    a. **Cardiopathy / Hypertension** numeric variable (1 = Yes; 2 = No).
    b. **Respiratory disease** numeric variable (1 = Yes; 2 = No).
    c. **Hepatopathy** numeric variable (1 = Yes; 2 = No).
    d. **Nephropathy** numeric variable (1 = Yes; 2 = No).
    e. **Active Cancer** numeric variable (1 = Yes; 2 = No).
    f. **Immunosuppression** numeric variable (1 = Yes; 2 = No).
    g. **Diabetes mellitus** numeric variable (1 = Yes; 2 = No).
    h. **Pregnancy** numeric variable (1 = Yes; 2 = No).
17. **Have you had contact with patients with COVID-19 infection at ICO?** numeric variable (1 = Yes; 2 = No).
18. **Have you had contact with samples of COVID-19 patients at ICO?** numeric variable (1 = Yes; 2 = No).

For peer review only - http://bmjopen.bmj.com/site/about/guidelines.xhtml

19. **When you are in your workplace, do you wear a surgical mask?** numeric variable (1 = Yes; 2 = No).
20. **If you are in the COVID-19area, do you wear Personal Protective Equipment (PPE)?** numeric variable (1 = Yes; 2 = No, 3 = Not Applicable).
21. **Do you think that the Personal Protective Equipment (PPE) removal procedure is safe?** numeric variable (1 = Yes; 2 = No, 3 = I don't know, 4 = Not Applicable).
22. **Do you feel protected by the Personal Protective Equipment (PPE) used?** numeric variable (1 = Yes; 2 = No, 3 = I don't know, 4 = Not Applicable).
23. **Do you think that you may have been exposed to COVID-19 during personal relationships with your co-workers?** numeric variable (1 = Yes; 2 = No, 3 = I don't know).
24. **Do you think that the protection procedures implemented during this pandemic period will benefit you in your future professional development?** numeric variable (1 = Yes; 2 = No, 3 = I don't know).
25. **Do you think that the work activity carried out during this pandemic period has affected you or will affect you emotionally in the future?** numeric variable (1 = Yes; 2 = No, 3 = I don't know).

At work, do you wash your hands with soap or water or with a hydro-alcoholic solution...

26. **... before you start working?** numeric variable (1 = Yes; 2 = No).
27. **... every time you enter a new workspace?** numeric variable (1 = Yes; 2 = No).
28. **... before eating?** numeric variable (1 = Yes; 2 = No).
29. **... after handling money, mobile phone, other utensils ...?** numeric variable (1 = Yes; 2 = No).
30. **... less than 7 times during the working day?** numeric variable (1 = Yes; 2 = No).
31. **... at the end of the working day?** numeric variable (1 = Yes; 2 = No).

32. **When you eat, do you maintain a distance ≥ 2m from your colleagues?** numeric variable (1 = Yes; 2 = No, 3 = Not Applicable).
33. **Did your collegues cover thir face with their elbows when they sneeze / cough?** numeric variable (1 = Yes; 2 = No).

C. COVID-19 exposure outside working environament (home and social activities).

Outside working environment, do you wash your hands (with soap and water or hydro-alcoholic solution)...

34. **... when you get home?** numeric variable (1 = Yes; 2 = No).
35. **... before eating?** numeric variable (1 = Yes; 2 = No).
36. **... after handling money, mobile phone, other utensils** numeric variable (1 = Yes; 2 = No).
37. **... after cleaning?** numeric variable (1 = Yes; 2 = No).
38. **... after blowing your nose, sneezing or coughing?** numeric variable (1 = Yes; 2 = No).
39. **Do you shower and change clothes when you get home (or did you go to work)?:** numeric variable (1 = Yes; 2 = No).
40. **Do you wear a mask when you go shopping?** numeric variable (1 = Yes; 2 = No)
41. **Do the people you live with cover their elbows if they sneeze / cough?** numeric variable (1 = Yes; 2 = No)
42. **Do you have a cohabitant who has passed COVID-19 (with symptoms, with or without confirmation by PCR, or PCR + without symptoms)?** numeric variable (1 = Yes; 2 = No).
43. **Do you use public transport to go to work?** numeric variable (1 = Yes; 2 = No)
    If "yes", continue with question 44; if "no , jump to question 46.
44. **Which type of public transport?** numeric variable (1 = bus, 2 = metro, 3 = train, 4 = taxi, 5 = bicycle (multiple answer allowed)).
45. **How many days a week do you use public transport?** | _ _ | numeric variable (1 to 7).
46. **Do you use private transportation to get to work?** numeric variable (1 = Yes; 2 = No).
    If "yes", continue with question 47; if "no", jump to question 49.
47. **Which private transport?** numeric variable (1 = single use car, 2 = shared car, 3 = single use bike, 4 = shared bike, 5 = bike (multiple answer allowed)).
48. **How many days per week do you use private transport?** | _ _ | numeric variable (1 to 7).

For peer review only - http://bmjopen.bmj.com/site/about/guidelines.xhtml

49. **Do you walk the street for more than 15 minutes?** numeric variable (1 = Yes; 2 = No).
    If "yes", continue with question 50; if "no", jump to question 52.
50. **How many days a week do you go for a walk?** | _ _ | numeric variable (1 to 7).
51. **For how many minutes a day do you go for a walk as an average:** | _ _ | minutes / day numeric variable.

D. COVID-19 tests performed

52. **Have you had a COVID-19 PCR test?** numeric variable (1 = Yes, only one; 2 = Yes, several; 3 = No).
    If "1", continue with question 53; if "2", jump to question 55; if "3", jump to question 59.
53. **COVID-19 PCR test performed on day:** | *dd* | *mm* | *yy*|.
54. **COVID-19 PCR test result** numeric variable (1 = Negative; 2 = Positive).
55. **How many COVID-19 PCR test have you had in total?** | _ _ | numeric variable (1 to 10).
    Depending on the answer, open as many questions with the number of PCR made to ask the date and result in the same format (A1 and A2; B1 and B2; etc).
    A1. **COVID-19 PCR test performed on day:** | *dd* | *mm* | *yy*|.
    A2. **COVID-19 PCR test result** numeric variable (1 = Negative; 2 = Positive).
56. **When you had your first COVID-19 PCR test, did you present any of these signs or symptoms?** numeric variable (multiple answer allowed) (1 = Febricula (>37.3ºC); 2 = Fever (>38°C); 3 = Cough; 4 = Odynophagia (sorethroat); 5 = Headache; 6 = Arthromyalgia (generalized pain); 7 = Asthenia (intense fatigue); 8 = Dyspnoea (shortness of breath); 9 = Anosmia (loss of smell); 10= Nausea, vomiting; 11 = Diarrhea; 12 = Skin lesions; 13 = Myoclonus (involuntary movements); 14 = Pneumonia; 15 = Other (specify: string variable __________________).
57. **Have you had a COVID-19 rapid antibody test?** numeric variable (1 = Yes; 2 = No).
58. **COVID-19 rapid antibody test result** numeric variable (1 = Negative; 2 = Positive).

Finally, we would like to complete the information provided with information about your lifestyle.

E. Lifestyle

59. **Do you drink any alcoholic beverage at least once a week?** numeric variable (1 = Yes; 2 = No).
    If "yes", continue with question 60; if "no", jump to question 64.
60. **How many glasses of wine do you drink every week?** | _ _ | numeric variable
61. **How many beers do you drink every week?** | _ _ | numeric variable
62. **How many glasses of cognac, gin or other spirits do you drink every week?** | _ _ | numeric variable
63. **Has your alcohol consumption changed during the pandemic compared to your consumption previously?** numeric variable (1 = No, it is similar; 2 = Yes, it has increased; 3 = Yes, it has decreased).
64. **Regarding tobacco use:** numeric variable (1 = I have never smoked; 2 = I am a former smoker; 3 = I am a corrent smoker).
    If "1" or "2", jump to question 67; If "3", continue to question 65.
65. **How many roll-ypur-own cigarrettes do you smoke every day?** | _ _ | numeric variable
66. **Has your tobacco consumption changed during the pandemic compared to your consumption previously?** numeric variable (1 = No, it is similar; 2 = Yes, it has increased; 3 = Yes, it has decreased).

F. End of the survey

Thank you very much for your participation. As mentioned before, all information from this survey and the tests performed is confidential and will be anonymized.

If you would like to leave us any further comments regarding the pandemic at the ICO Centers, please do so below:

67. **Commentaries**. *Open answer, leave space for about 5 lines of text.*

Supplementary Table 1. Demographic characteristics of on-site workers (always/ocassionally) and teleworkers

	Teleworking		
	Never/ocassionally (n=981) n (%)	Always (n=230) n (%)	*p-value*
**Sex**			
Male	240 (25)	47 (20)	
Female	736 (75)	183 (80)	*0,183*
**Age [median, (min-max)]**	43 (19-68.5)	44.9 (19-71.6)	*0,015*
<35y	271 (28)	38 (17)	
35-49y	429 (44)	122 (53)	
>49y	281 (29)	70 (30)	*0,002*
**ICO Center**			
ICO L'Hospitalet	684 (70)	184 (80)	
ICO Girona	182 (19)	17 (7)	
ICO Badalona	103 (11)	29 (13)	
ICO Tarragona / Terres de l'Ebre	12 (1)	0 (0)	*<0.0001*
**Health care workers**			
Yes	567 (59)	72 (32)	
No	402 (41)	152 (68)	*<0.0001*
Middle and superior technicians	*187 (19)*	*92 (41)*	
Porters	*17 (2)*	*3 (1)*	
Administrative staff	*90 (9)*	*35 (16)*	
Maintenance or security stafft	*29 (3)*	*2 (1)*	
Cleaning staff	*46 (5)*	*15 (7)*	
Restoration staff	*16 (2)*	*2 (1)*	
Others	*17 (2)*	*3 (1)*	*<0.0001*
**Any Comorbidity**	142 (15)	38 (17)	*0,4*
**Smoking history**			
Never	511 (54)	126 (56)	
Ever	438 (46)	98 (44)	*0,5*
Past	277 (29)	66 (29)	
Current	161 (17)	32 (14)	*0,6*
**Cohabiting**	889 (91)	209 (92)	*0,8*
**Cohabiting with covid-19**	115 (14)	27 (14)	*0,9*
**Reported rRT-PCR previous to serology**	422 (84)	42 (75)	*0,1*
**Positive of previous rRT-PCR**	62 (15)	10 (24)	*0,1*

For peer review only

Supplementary Table 2. Clinical characteristics associated with SARS-CoV-2 positive serology among those who report rRT-PCR previous to study serology (n=469).

	Total participants n (%)	SARS-CoV-2 seroprevalence n (%)	Prevalence (95%CI)	p-value[1]	Adjusted PR (95% CI)[2]
**Reported rRT-PCR previous to serology**	469 (38.0)	86 (78.2)	18.34 (15.08-22.11)		
**Result of previous rRT-PCR**					
Negative	397 (84.6)	27 (31.0)	6.80 (4.70-9.74)		REF
Positive	72 (15.4)	59 (68.6)	81.94 (71.31-89.23)	<0.001	12.15 (7.54-19.57)
**Number of symptoms***(mean, standard deviation)*	1.65 (2.10)	3.08 (2.61)		<0.001	
None	217 (46.3)	21 (24.0)	9.68 (6.39-14.4)		REF
One	61 (13)	7 (8.1)	11.48 (5.56-22.21)		1.13 (0.48-2.67)
2-3	109 (23.2)	22 (25.6)	20.18 (13.66-28.78)		2.03 (1.10-3.73)
≥4	81 (17.3)	35 (40.7)	43.21 (32.87-54.18)	<0.001	4.33 (2.48-7.59)
p-trend (among exposed)					*<0.001*
**Reporting COVID-19 compatible symptoms when rRT-PCR was performed**					
No	217 (46.3)	21 (24.0)	9.68 (6.39-14.4)		REF
Yes	251 (53.5)	64 (74.4)	25.5 (20.48-31.27)	<0.001	2.49 (1.51-4.10)
**COVID-19 symptoms**					
Headache	126 (26.9)	36 (41.9)	28.57 (21.35-37.08)	<0.001	1.87 (1.20-2.93)
Cough	119 (25.4)	37 (43.0)	31.09 (23.42-39.97)	<0.001	2.25 (1.44-3.52)
Asthenia	110 (23.5)	36 (41.9)	32.73 (24.6-42.04)	<0.001	2.38 (1.53-3.72)
Arthromyalgia	80 (17.1)	57 (66.0)	36.25 (26.47-47.31)	<0.001	2.32 (1.47-3.67)
Low-grade fever (37.3ºC-38ºC)	73 (15.6)	26 (30.2)	35.62 (25.5-47.21)	<0.001	2.71 (1.67-4.39)
Odynophagia	64 (13.6)	14 (16.3)	21.88 (13.39-33.65)	0.40	1.18 (0.65-2.13)
Diarrhoea	58 (12.4)	16 (18.6)	27.59 (17.62-40.43)	0.05	1.47 (0.83-2.60)
Anosmia	42 (9)	33 (38.4)	78.57 (63.65-88.48)	<0.001	6.09 (3.86-9.60)
Dyspnoea	40 (8.5)	11 (12.8)	27.50 (15.91-43.2)	0.12	1.56 (0.81-3.00)
Fever (>38ºC)	28 (6)	15 (17.4)	53.57 (35.4-70.84)	<0.001	3.06 (1.71-5.46)
Nausea / vomiting	17 (3.6)	6 (7)	35.29 (16.75-59.66)	0.07	1.86 (0.80-4.36)
Skin lesions	8 (1.7)	1 (1.2)	12.50 (1.72-53.86)	0.66	0.74 (0.10-5.38)
Pneumonia	3 (0.6)	2 (2.3)	66.67 (15.27-95.69)	0.03	2.99 (0.71-12.63)
Myoclonus	2 (0.4)	0		0.50	

Numbers do not always sum up the total due to some missing values (none of the categories present more than 5% of missing values). PR: Prevalence Ratio, CI: Confidence Interval. [1] Chi-squared test for categorical variables (Fisher's exact test corrected for continuity) and median test for continuous variables. [2] Adjusted for sex, age (continuous), ICO center, care staff, telework and cohabitants.

For peer review only

STROBE 2007 (v4) Statement—Checklist of items that should be included in reports of *cross-sectional studies*

Section/Topic	Item #	Recommendation	Reported on page #
**Title and abstract**	1	(*a*) Indicate the study's design with a commonly used term in the title or the abstract	3
		(*b*) Provide in the abstract an informative and balanced summary of what was done and what was found	3
**Introduction**			
Background/rationale	2	Explain the scientific background and rationale for the investigation being reported	5
Objectives	3	State specific objectives, including any prespecified hypotheses	5
**Methods**			
Study design	4	Present key elements of study design early in the paper	5
Setting	5	Describe the setting, locations, and relevant dates, including periods of recruitment, exposure, follow-up, and data collection	5
Participants	6	(*a*) Give the eligibility criteria, and the sources and methods of selection of participants	5
Variables	7	Clearly define all outcomes, exposures, predictors, potential confounders, and effect modifiers. Give diagnostic criteria, if applicable	7
Data sources/ measurement	8*	For each variable of interest, give sources of data and details of methods of assessment (measurement). Describe comparability of assessment methods if there is more than one group	7
Bias	9	Describe any efforts to address potential sources of bias	5,6,19
Study size	10	Explain how the study size was arrived at	5
Quantitative variables	11	Explain how quantitative variables were handled in the analyses. If applicable, describe which groupings were chosen and why	7
Statistical methods	12	(*a*) Describe all statistical methods, including those used to control for confounding	8
		(*b*) Describe any methods used to examine subgroups and interactions	8
		(*c*) Explain how missing data were addressed	8
		(*d*) If applicable, describe analytical methods taking account of sampling strategy	NA
		(*e*) Describe any sensitivity analyses	NA
**Results**			

For peer review only

Participants	13*	(a) Report numbers of individuals at each stage of study—eg numbers potentially eligible, examined for eligibility, confirmed eligible, included in the study, completing follow-up, and analysed	9
		(b) Give reasons for non-participation at each stage	9
		(c) Consider use of a flow diagram	6
Descriptive data	14*	(a) Give characteristics of study participants (eg demographic, clinical, social) and information on exposures and potential confounders	9
		(b) Indicate number of participants with missing data for each variable of interest	9
Outcome data	15*	Report numbers of outcome events or summary measures	12
Main results	16	(*a*) Give unadjusted estimates and, if applicable, confounder-adjusted estimates and their precision (eg, 95% confidence interval). Make clear which confounders were adjusted for and why they were included	12
		(*b*) Report category boundaries when continuous variables were categorized	9
		(*c*) If relevant, consider translating estimates of relative risk into absolute risk for a meaningful time period	
Other analyses	17	Report other analyses done—eg analyses of subgroups and interactions, and sensitivity analyses	15
**Discussion**			
Key results	18	Summarise key results with reference to study objectives	17
Limitations	19	Discuss limitations of the study, taking into account sources of potential bias or imprecision. Discuss both direction and magnitude of any potential bias	21
Interpretation	20	Give a cautious overall interpretation of results considering objectives, limitations, multiplicity of analyses, results from similar studies, and other relevant evidence	17
Generalisability	21	Discuss the generalisability (external validity) of the study results	21
**Other information**			
Funding	22	Give the source of funding and the role of the funders for the present study and, if applicable, for the original study on which the present article is based	22

*Give information separately for cases and controls in case-control studies and, if applicable, for exposed and unexposed groups in cohort and cross-sectional studies.

Note: An Explanation and Elaboration article discusses each checklist item and gives methodological background and published examples of transparent reporting. The STROBE checklist is best used in conjunction with this article (freely available on the Web sites of PLoS Medicine at http://www.plosmedicine.org/, Annals of Internal Medicine at http://www.annals.org/, and Epidemiology at http://www.epidem.com/). Information on the STROBE Initiative is available at www.strobe-statement.org.

For peer review only

COVID-19 among workers of a Comprehensive Cancer Center between first and second epidemic waves (2020): a seroprevalence study in Catalonia, Spain.

Journal:	*BMJ Open*
Manuscript ID	bmjopen-2021-056637.R2
Article Type:	Original research
Date Submitted by the Author:	23-Feb-2022
Complete List of Authors:	Peremiquel-Trillas , Paul; Catalan Institute of Oncology, Tobacco Control Unit<br>Saura-Lazaro, Anna; Catalan Institute of Oncology, Tobacco Control Unit<br>Benavente Moreno, Yolanda; Catalan Institute of Oncology Cancer Epidemiology Research Programme<br>Casabonne, Delphinne; Catalan Institute of Oncology<br>Loureiro, Eva; Catalan Institute of Oncology<br>Cabrera, Sandra; Catalan Institute of Oncology<br>Duran, Angela; Catalan Institute of Oncology<br>Garrote, Lidia; Catalan Institute of Oncology<br>Brao, Immaculada; Catalan Institute of Oncology<br>Trelis, Jordi; Catalan Institute of Oncology<br>Galán, Maica; Catalan Institute of Oncology<br>Soler, Francesc; Catalan Institute of Oncology<br>Julia, Joaquim; Catalan Institute of Oncology<br>Cortasa, Dolça; Catalan Institute of Oncology<br>Domínguez, Maria Ángeles; Bellvitge University Hospital<br>Albasanz-Puig, Adaia; Catalan Institute of Oncology<br>Gudiol, Carlota; Catalan Institute of Oncology<br>Ramírez-Tarruella, Dolors; Catalan Institute of Oncology<br>Muniesa, Joan; Catalan Institute of Oncology<br>Rivas, Juan; Catalan Institute of Oncology<br>Muñoz-Montplet, Carles; Catalan Institute of Oncology<br>Sedano, Ana; Catalan Institute of Oncology<br>Plans, Àngel; Catalan Institute of Oncology<br>Calvo-Cerrada, Beatriz; Catalan Institute of Oncology<br>Calle, Candela; Catalan Institute of Oncology<br>Clopés, Ana; Catalan Institute of Oncology<br>Carnicer-Pont, Dolors; Hospital Duran i Reynals,<br>Alemany, L.; Institut Catala d' Oncologia,<br>Fernandez, Esteve; Catalan Institute of Oncology, Tobacco Control Unit
**Primary Subject Heading**:	Epidemiology
Secondary Subject Heading:	Infectious diseases, Oncology
Keywords:	Adult oncology < ONCOLOGY, Epidemiology < INFECTIOUS DISEASES, COVID-19, Epidemiology < ONCOLOGY

SCHOLARONE™
Manuscripts

I, the Submitting Author has the right to grant and does grant on behalf of all authors of the Work (as defined in the below author licence), an exclusive licence and/or a non-exclusive licence for contributions from authors who are: i) UK Crown employees; ii) where BMJ has agreed a CC-BY licence shall apply, and/or iii) in accordance with the terms applicable for US Federal Government officers or employees acting as part of their official duties; on a worldwide, perpetual, irrevocable, royalty-free basis to BMJ Publishing Group Ltd ("BMJ") its licensees and where the relevant Journal is co-owned by BMJ to the co-owners of the Journal, to publish the Work in this journal and any other BMJ products and to exploit all rights, as set out in our licence.

The Submitting Author accepts and understands that any supply made under these terms is made by BMJ to the Submitting Author unless you are acting as an employee on behalf of your employer or a postgraduate student of an affiliated institution which is paying any applicable article publishing charge ("APC") for Open Access articles. Where the Submitting Author wishes to make the Work available on an Open Access basis (and intends to pay the relevant APC), the terms of reuse of such Open Access shall be governed by a Creative Commons licence – details of these licences and which Creative Commons licence will apply to this Work are set out in our licence referred to above.

Other than as permitted in any relevant BMJ Author's Self Archiving Policies, I confirm this Work has not been accepted for publication elsewhere, is not being considered for publication elsewhere and does not duplicate material already published. I confirm all authors consent to publication of this Work and authorise the granting of this licence.

For peer review only

COVID-19 among workers of a Comprehensive Cancer Center between first and second epidemic waves (2020): a seroprevalence study in Catalonia, Spain.

Paula Peremiquel-Trillas, Anna Saura-Lázaro*, Yolanda Benavente-Moreno, Delphine Casabonne, Eva Loureiro, Sandra Cabrera, Angela Durán, Lidia Garrote, Immaculada Brao, Jordi Trelis, Maica Galán, Francesc Soler, Joaquim Julià, Dolça Cortasa, M. Angeles Domínguez, Adaia Albasanz-Puig, Carlota Gudiol, Dolors Ramírez, Joan Muniesa, Juan Pedro Rivas, Carles Muñoz-Montplet, Ana Sedano, Àngel Plans, Beatriz Calvo-Cerrada, Candela Calle, Ana Clopés, Dolors Carnicer-Pont†, Laia Alemany†, Esteve Fernández†*

*Contributed equally

†Joint senior authors

Authors' affiliations

PPT Cancer Epidemiology Research Programme, Cancer Epidemiology and Prevention Department, Institut Català d'Oncologia (ICO), L'Hospitalet de Llobregat, Spain; Epidemiology and Public Health Programme, Institut d'Investigació Biomèdica de Bellvitge (IDIBELL) , L'Hospitalet de Llobregat, Spain; CIBER of Epidemiology and Public Health (CIBERESP), Madrid, Spain; School of Medicine and Clinical Sciences, Universitat de Barcelona, Barcelona, Spain. @PeremiquelPaula. ORCID: 0000-0002-8636-1725

ASL Cancer Epidemiology Research Programme, Cancer Epidemiology and Prevention Department, Institut Català d'Oncologia (ICO), L'Hospitalet de Llobregat, Spain; Epidemiology and Public Health Programme, Institut d'Investigació Biomèdica de Bellvitge (IDIBELL) , L'Hospitalet de Llobregat, Spain. 0000-0001-9742-2725

YBM Cancer Epidemiology Research Programme, Cancer Epidemiology and Prevention Department, Institut Català d'Oncologia (ICO) , L'Hospitalet de Llobregat, Spain; Epidemiology and Public Health Programme, Institut d'Investigació Biomèdica de Bellvitge (IDIBELL) , L'Hospitalet de Llobregat, Spain; CIBER of Epidemiology and Public Health (CIBERESP), Madrid, Spain. ORCID: 0000-0003-1422-4614

DC Cancer Epidemiology Research Programme, Cancer Epidemiology and Prevention Department, Institut Català d'Oncologia (ICO) , L'Hospitalet de Llobregat, Spain; Epidemiology and Public Health Programme, Institut d'Investigació Biomèdica de Bellvitge (IDIBELL) , L'Hospitalet de Llobregat, Spain; CIBER of Epidemiology and Public Health (CIBERESP), Madrid, Spain. ORCID: 0000-0002-7874-3707

EL Computer Science Services, Technology & Physics, Institut Català d'Oncologia (ICO) , L'Hospitalet de Llobregat, Spain; Computational Science and Artificial Intelligence, Faculty of Computer Science of Coruña, University of Coruña (UDC), Coruña, Spain.

SC Research Nursing Department, Institut Català d'Oncologia (ICO), L'Hospitalet de Llobregat, Spain. @scabrerajaime. ORCID: 0000-0003-3013-8812

AD Nursing Department, Institut Català d'Oncologia (ICO), L'Hospitalet de Llobregat, Spain.

LG Nursing Department, Institut Català d'Oncologia – Badalona (ICO), Badalona, Spain.

IB Nursing Department, Institut Català d'Oncologia – Girona (ICO), Girona, Spain.

JT Palliative Care Department and Medical Director, Institut Català d'Oncologia (ICO), L'Hospitalet de Llobregat, Spain. @JordiTrelis.

MG Esofagogastric Tumours Functional Unit and Medical Director, Institut Català d'Oncologia (ICO), L'Hospitalet de Llobregat, Spain.

FS Pharmacy Service and Medical Director, Institut Català d'Oncologia – Girona (ICO), Girona, Spain.

JJT Palliative Care Department and Medical Director. Institut Català d'Oncologia (ICO) Badalona, Badalona, Spain. @drjjulia. ORCID: 0000-0002-1462-3167

DC Medical Director, Institut Català d'Oncologia -Tarragona/Terres de l'Ebre, Tarragona (ICO).

MADL Microbiology Department. Hospital Universitari de Bellvitge; Infectious Diseases Programme, Institut d'Investigació Biomèdica de Bellvitge (IDIBELL); Department of Pathology Experimental Therapeutics, Universitat de Barcelona, Barcelona, Spain.

For peer review only

For peer review only - http://bmjopen.bmj.com/site/about/guidelines.xhtml

AAP Infectious Disease Department, Hospital Universitari de Bellvitge, Institut Català d'Oncologia (ICO), L'Hospitalet de Llobregat, Spain. CIBER of Infectious Diseases (CIBERINFEC), Madrid, Spain. ORCID: 0000-0001-9852-5574.

CG Infectious Disease Department, Hospital Universitari de Bellvitge, Institut Català d'Oncologia (ICO), L'Hospitalet de Llobregat, Spain. CIBER of Infectious Diseases (CIBERINFEC), Madrid, Spain. @Carlotasway3. ORCID: 0000-0003-3095-4422

DR Preventive Medicine Unit, Institut Català d'Oncologia (ICO), L'Hospitalet de Llobregat, Spain.

JM Computer Science Services, Technology & Physics Department, Institut Català d'Oncologia (ICO), L'Hospitalet de Llobregat, Spain.

JPR Computer Science Services, Technology & Physics Department, Institut Català d'Oncologia (ICO), L'Hospitalet de Llobregat, Spain.

CMM Technology and Physics Department, Institut Català d'Oncologia (ICO), L'Hospitalet de Llobregat, Spain; Medical Physics and Radiation Protection Department, Institut Català d'Oncologia (ICO) – Girona, Girona, Spain; Department of Medical Sciences, Universitat de Girona, Girona, Spain. @CMunozMontplet. ORCID: 0000-0002-7324-8889.

AS Human Resources Department, Institut Català d'Oncologia (ICO), L'Hospitalet de Llobregat, Spain.

AC Scientific Direction, Institut Català d'Oncologia (ICO), L'Hospitalet de Llobregat, Spain. ORCID: 0000-0002-1857-362X.

BCC Occupational Health Unit, Institut Català d'Oncologia (ICO), L'Hospitalet de Llobregat, Spain.

CC General Direction, Institut Català d'Oncologia (ICO), L'Hospitalet de Llobregat, Spain. @CandelaCalle.

AP Occupational Health Unit, Institut Català d'Oncologia (ICO), L'Hospitalet de Llobregat, Spain. ORCID: 0000-0003-3199-0361.

DCP Cancer Prevention and Control Programme, Cancer Epidemiology and Prevention Department, Catalan Institute of Oncology (ICO) , L'Hospitalet de Llobregat, Spain; Epidemiology and Public Health Programme, Institut d'Investigació Biomèdica de Bellvitge (IDIBELL), L'Hospitalet de Llobregat, Spain. ; CIBER of Respiratory Diseases (CIBERES). ORCID: 0000-0002-3475-8704.

LA Cancer Epidemiology Research Programme, Cancer Epidemiology and Prevention Department, Institut Català d'Oncologia (ICO) , L'Hospitalet de Llobregat, Spain; Epidemiology and Public Health Programme, Institut d'Investigació Biomèdica de Bellvitge (IDIBELL), L'Hospitalet de Llobregat, Spain; CIBER of Epidemiology and Public Health (CIBERESP), Madrid, Spain. ORCID: 0000-0003-0945-6015.

EF Cancer Prevention and Control Programme, Cancer Epidemiology and Prevention Department, Institut Català d'Oncologia (ICO) , L'Hospitalet de Llobregat, Spain; Epidemiology and Public Health Programme, Institut d'Investigació Biomèdica de Bellvitge (IDIBELL), L'Hospitalet de Llobregat, Spain; School of Medicine and Clinical Sciences, Universitat de Barcelona, Barcelona, Spain; CIBER of Respiratory Diseases (CIBERES). @StvFdz. ORCID: 0000-0003-4239-723X.

Word counts.

Abstract: 296. Main text: 3,309. One figure, three tables, supplementary information.

Correspondence to: Prof. Esteve Fernández, MD, MPH, PhD. Tobacco Control Unit, Cancer Prevention and Control Programme, Institut Català d'Oncologia (ICO). Avinguda de la Granvia de l'Hospitalet, 199-203, 08908 L'Hospitalet de Llobregat, Barcelona, Spain. Phone: +34 93 2607345. Twitter: @StvFdz E-mail: efernandez@iconcologia.net (Prof. E. Fernández).

For peer review only - http://bmjopen.bmj.com/site/about/guidelines.xhtml

ABSTRACT

Objectives Cancer patients are at higher risk for severe COVID-19 infection. COVID-19 surveillance of workers in oncological centres is crucial to assess infection burden and prevent transmission. We estimate the SARS-CoV-2 seroprevalence among health care workers (HCW) of a comprehensive cancer centre in Catalonia, Spain, and analyse its association with sociodemographic characteristics, exposure factors and behaviours.

Design Cross-sectional study (21st May – 26th June 2020).

Setting A comprehensive cancer centre (Institut Català d'Oncologia) in Catalonia, Spain.

Participants All HCW (*N*=1,969) were invited to complete an online self-administered epidemiological survey and provide a blood sample for SARS-CoV-2 antibodies detection.

Primary outcome measure Prevalence (%) and 95% confidence intervals (CI) of seropositivity together with adjusted prevalence ratios (aPR) and 95%CI were estimated.

Results A total of 1,266 HCW filled the survey (participation rate: 64.0%) and 1,238 underwent serological testing (97.8%). The median age was 43.7 years (p25-p75: 34.8-51.0 years), 76.0% were female, 52.0% were nursing or medical staff, and 79.0% worked on-site during the pandemic period. SARS-CoV-2 seroprevalence was 8.9% (95%CI: 7.44-10.63), with no differences by age and sex. No significant differences in terms of seroprevalence were observed between onsite workers and teleworkers. Seropositivity was associated with living with a person with COVID-19 (aPR: 3.86, 95%CI: 2.49-5.98). Among on-site workers, seropositive participants were twofold more likely to be nursing or medical staff. Nursing and medical staff working in a COVID-19 area showed a higher seroprevalence than other staff (aPR: 2.45, 95% CI: 1.08-5.52).

Conclusions At the end of the first wave of the pandemic in Spain, SARS-CoV-2 seroprevalence among Institut Català d'Oncologia HCW was lower than the reported in other Spanish hospitals. The main risk factors were sharing household with infected people and contact with COVID-19 patients and colleagues. Strengthening preventive measures and health education among HCW is fundamental.

Keywords SARS-CoV-2; COVID-19; seroprevalence; antibody; health care workers; epidemiology.

For peer review only

ARTICLE SUMMARY

Strengths and limitations

- Seroepidemiological study with a large sample size settled in a comprehensive cancer center.
- Questionnaire completeness was very high, with no variables presenting more than 5% of missing values.
- Recall bias is possible as the data for the correlates of SARS-CoV-2 infection rely on a self-administered questionnaire.
- The accomplishment of preventive measures might be overestimated: response and perception biases must be considered, as well as complacency bias.
- Answers reported in the questionnaire could be influenced by the participants' knowledge regarding their COVID status.

INTRODUCTION

Frontline health care workers (HCW) dealing with COVID-19 have higher exposure to SARS-CoV-2 than the general population (1), and they can contribute to the spread of COVID-19 as per their exposure to vulnerable patients. Since the beginning of the pandemic, several studies have been published on SARS-CoV-2 infections prevalence in HCW, although with diverse results. A meta-analysis of 49 studies, including 127,480 health care workers, showed that the overall seroprevalence of SARS-CoV-2 antibodies in the European region was 8.5% (2). HCW in Spain have been highly affected: a total amount of 154,636 cases among HCW were already officially notified by December 2, 2021 at the onset of the sixth pandemic wave (3,4).

Cancer patients are vulnerable, presenting a high risk for COVID-19 infection and more severe outcomes due to their immunosuppression status (5). The pandemic has presented unprecedented professional and personal challenges for the oncology community (6). Data are lacking on the seroprevalence of SARS-CoV-2 among HCW in oncological centres, and small sample sizes limit the few published studies. The present study aims to estimate the seroprevalence of SARS-CoV-2 and associated sociodemographic and behavioural risk factors among workers of the Catalan Institute of Oncology (ICO), a Comprehensive Cancer Centre comprised of four hospitals in Catalonia (Spain), covering around 40% of the adult population in Catalonia (7).

For peer review only - http://bmjopen.bmj.com/site/about/guidelines.xhtml

PARTICIPANTS AND METHODS

Study design and setting

A cross-sectional study including blood sample collection and a self-administered questionnaire was conducted between 21st May and 26th June 2020 in the four ICO centres (L'Hospitalet de Llobregat, Badalona, Tarragona/Terres de l'Ebre and Girona).

The study population were HCW delivering care and services to patients (directly or indirectly) and support staff, including those who do not deliver care but work in other tasks within the hospital. A total of 1,969 employees of ICO were invited to participate in the study through an email that allowed access to the study information. The inclusion criteria were: a) to be an active worker during the epidemic period, (1st February - 26th June 2020) and b) to be aged ≥18years. The participants filled in an online epidemiological questionnaire and were scheduled for serology testing by the Occupational Health Department. 1,266 HCW filled in the online epidemiological questionnaire (participation rate: 64.3%) and 1,238 of them (97.8%) underwent a serology test. Three participants with inconclusive serological results were excluded. The final analysis included 1,235 participants (**Figure 1**).

Figure 1 about here

Epidemiological questionnaire and study variables

An epidemiological questionnaire was programmed online to collect information regarding sociodemographic characteristics, working information, compliance of personal protective equipment (PPE) measures at work, at home and history of previous COVID-19 infection (**Supplemental Material**). The questionnaire was developed based on previous epidemiological studies conducted within the ICO centres, and a modified version was used in another seroprevalence study performed among university personnel of the University of Barcelona (8).

Sociodemographic characteristics included information on age and sex, ICO centre of recruitment, presence of comorbidities, smoking history, pregnancy and cohabitants.

For peer review only - http://bmjopen.bmj.com/site/about/guidelines.xhtml

Work-related conditions included the professional category, teleworking status, type of shift, working on a COVID-19 area, contact with COVID-19 cases, contact with biological samples and reporting to be exposed to COVID-19.

Concerning PPE measures at work, participants were asked about feeling protected with PPE and compliance with PPE measures. Regarding the application of preventive measures outside the working setting, participants were asked if they got a shower after leaving the workplace or when arriving home, if they changed clothes after work or upon home arrival, as well as about hand washing and use of face mask when shopping. Information about COVID-19 cases and protective measures were also collected among those participants reporting cohabitants. Participants were also asked about the type of transport used to go to work.

Participants were asked about a previous diagnosis of COVID-19 performed by rRT-PCR or serology test and date of diagnosis, as well as reporting COVID-19 compatible symptoms, and the type of symptoms.

SARS-CoV-2 laboratory testing

Serum samples from participants at L'Hospitalet, Girona and Tarragona/Terres de l'Ebre were studied at the Microbiology Department of Hospital de Bellvitge and samples from health-care workers at ICO Badalona were analysed at the MetroNord Regional Clinical Laboratory, using the same procedures and techniques in both laboratories. Detection of SARS-CoV-2 antibodies was carried out using the quantitative SARS-CoV-2 S1/S2 IgG LIAISON® test (DiaSorin, Vercelli, Italy) on the LIAISON XL platform, following the manufacturer's instructions. This test discriminates among negative (<12AU/mL; with 3.8 as IgG detection limit), equivocal (12.0–15.0AU/mL) and positive (>15.0AU/mL) subjects. In those cases in which a) IgG anti S1/S2 quantification was higher than the limit of detection (i.e.>3.8AU/mL) but did not reach the limit of discrimination (i.e.<15AU/mL) and/or b) when the HCW answered the questionnaire saying that he or she had been diagnosed of COVID-19 but IgG anti S1/S2 where lower than 15 AU/ml, an additional serological study was performed using a different antigen (N) as a target. In this case, a SARS-CoV-2 IgG test (Abbott Diagnostics, Sligo, Ireland) was run on an Architect i2000 platform. This test discriminates among negative (<1.4Index (S/C)) and positive (≥1.4Index (S/C)) subjects.

For peer review only - http://bmjopen.bmj.com/site/about/guidelines.xhtml

Case definition

A seropositive case of SARS-CoV-2 was defined as seropositivity to IgG independently of previous self-reported results.

Patient and Public Involvement

No patient was involved in the study.

Statistical analysis

Crude global and by subgroups SARS-CoV-2 seroprevalence and 95% confidence intervals (CI) were calculated. Differences in the distribution of study variables between seropositive and seronegative participants were assessed using chi-squared test for categorical variables, and parametric or non-parametric tests were performed for normal and non-normal continuous variables, respectively. Prevalence ratios (PR) and 95% confidence intervals (CI) were estimated using Poisson regression models with robust variance (9). Prevalence ratios were adjusted (aPR) for statistically significant variables in the bivariate analysis and those considered relevant for the study design. Thus, adjusted models included sex, ICO centre of recruitment, age, type of HCW, teleworking and cohabitants. Linear trends for variables with ordinal categories was based in the likelihood ratio test of the model with the ordinal variable as a continuous one. *P*-values were based on 2-sided hypothesis tests and considered significant at $p<0.05$. All analyses were conducted using Stata version 16.0 (StataCorp LP, College Station, Texas).

For peer review only

Ethical considerations

The present study was approved by the Hospital Universitari de Bellvitge Ethics Committee (PR205/20). The study follows the Helsinki Declaration and subsequent amendments, and Spanish data confidentiality laws (General data protection regulation Organic Law 3/2018, EU General data protection Regulation 2016/679 and Law 14/2007 for biomedical research). All participants signed an informed consent form after receiving information of the study and prior to obtaining biological samples. The biological material obtained was kept at ICO and processed under the appropriate measures to preserve the confidentiality of the results and data.

For peer review only - http://bmjopen.bmj.com/site/about/guidelines.xhtml

RESULTS

A total of 1,235 HCW with serological results **(Figure 1)** were included in the analysis: 76.0% were female, the median age was 43.7 years (p25-p75: 34.8-51.0 years), 52.2% were nursing or medical staff, and 18.6% of the participants teleworked full-time during the study period (**Table 1**). Up to 14.7% of the participants reported at least one comorbidity. Regarding smoking habits, 16.0% were current smokers, and 28.2% reported to be former smokers (**Table 1**). Seven women were pregnant, and none of them showed seropositivity.

The overall crude SARS-CoV-2 seroprevalence was 8.9% (95%CI: 7.44-10.63), with no statistically significant differences by neither age group nor sex, and the seroprevalence for nursing and medical staff was 11.6% (95%CI: 9.37-14.34). After fully adjustment, the main determinants of higher seroprevalence included working at ICO Girona compared to workers at ICO L'Hospitalet (aPR: 1.52, 95%CI: 0.97-2.38), and nursing or medical staff compared to other groups (aPR: 2.04, 95%CI: 1.33-3.14) (**Table 1**).

For peer review only

Table 1. Sociodemographic characteristics associated with SARS-CoV-2 positive serology among study participants (*N*=1,235).

	**Total participants** *n* (%)	**SARS-CoV-2 seroprevalence** *n* (%)	**Prevalence (95%CI)**	***p-value*** [6]	**aPR (95% CI)** [7]
**Study participants**	1,235	110	8.91 (7.44-10.63)		
**Sex**					
Male	291 (23.6)	27 (24.5)	9.28 (6.44-13.20)		REF
Female	939 (76.0)	83 (75.5)	8.84 (7.18-10.83)	*0.82*	0.82 (0.53-1.28)
**Age [median, (p25-p75)]**	43.7 (34.8-51.0)	42.8 (32.0-50.1)		*0.62*	0.99 (0.97-1.01)
<35 years	313 (25.3)	33 (30.0)	10.54 (7.59-14.46)		REF
35-49 years	566 (45.8)	47 (42.7)	8.30 (6.29-10.88)		0.85 (0.55-1.34)
>49 years	356 (28.8)	30 (27.3)	8.43 (5.95-11.80)	*0.5*	0.88 (0.53-1.46)
**ICO Center**					
ICO L'Hospitalet	885 (71.7)	73 (66.4)	8.25 (6.61-10.25)		REF
ICO Girona	204 (16.5)	29 (26.4)	14.22 (10.06-19.72)		1.52 (0.97-2.38)
ICO Badalona	134 (10.9)	7 (6.4)	5.22 (2.51-10.56)		0.54 (0.25-1.19)
ICO Tarragona / Terres de l'Ebre	12 (1.0)	1 (0.9)	8.33 (1.16-41.38)	*0.02*	1.07 (0.15-7.83)
**Professional category**					
Nursing staff[1]	380 (30.8)	43 (39.0)	11.32 (8.50-14.92)		REF
Medical Staff[2]	265 (21.5)	32 (29.1)	12.08 (8.67-16.58)		1.07 (0.65-1.76)
Middle and superior technicians	285 (23.1)	14 (12.7)	4.91 (2.93-8.13)		0.41 (0.22-0.77)
Service staff[3]	114 (9.2)	2 (1.8)	7.02 (3.55-13.42)		0.69 (0.31-1.54)
Porter	21 (1.7)	8 (7.3)	9.52 (2.39-31.16)		0.74 (0.17-3.24)
Administrative	129 (10.4)	8 (7.3)	6.20 (3.13-11.92)		0.54 (0.25-1.16)
Other	20 (1.6)	1 (0.9)	5.00 (0.70-28.26)	*0.03*	0.50 (0.07-3.71)
Nursing or medical staff[4]	645 (52.2)	75 (68.2)	11.63 (9.37-14.34)	*<0.001*	2.04 (1.33-3.14)
Other staff[5]	569 (46.1)	33 (30.0)	5.80 (4.15-8.05)		REF
**Telework**					
Never/Occasionally	981 (79.4)	86 (78.1)	8.77 (7.15-10.71)		REF
Always	230 (18.6)	23 (20.9)	10.00 (6.72-14.63)	*0.56*	1.60 (0.98-2.59)

For peer review only - http://bmjopen.bmj.com/site/about/guidelines.xhtml

Table 1 (continued)

	Total participants	SARS-CoV-2 seroprevalence	Prevalence (95%CI)	*p-value*[7]	aPR (95% CI)[8]
**Shift work**					
Morning	545 (44.1)	49 (45.0)	8.99 (6.86-11.7)		REF
Evening	140 (11.3)	10 (9.1)	7.14 (3.88-12.77)		0.56 (0.34-0.93)
Split shift (morning-evening)	417 (33.8)	38 (34.5)	9.11 (6.7-12.28)		0.88 (0.57-1.37)
Night	88 (7.1)	10 (9.1)	11.36 (6.22-19.86)		0.95 (0.46-1.96)
Other	25 (2)	3 (2.7)	12 (3.92-31.32)	*0.83*	1.15 (0.35-3.75)
**Comorbidities**[6]					
None	1,054 (85.3)	99 (90.0)	9.39 (7.77-11.31)		REF
Yes	181 (14.7)	11 (10.0)	6.08 (3.4-10.64)	*0.15*	0.67 (0.36-1.25)
**Smoking history**					
Never	650 (52.6)	80 (72.7)	12.31 (9.99-15.07)		REF
Past	348 (28.2)	22 (20.0)	6.32 (4.20-9.42)		0.57 (0.35-0.93)
Current	198 (16.0)	8 (7.3)	4.04 (2.03-7.87)	*0.0002*	0.38 (0.18-0.79)
**Cohabitants**					
Yes	1,119 (90.6)	95 (86.0)	8.49 (6.99-10.27)		REF
No	104 (8.4)	15 (13.6)	14.42 (8.88-22.57)	*0.04*	1.48 (0.83-2.66)

194 Numbers do not always sum up the total due to some missing values (none of the categories present more than 5% of missing values).
195 aPR: adjusted Prevalence Ratio, CI: Confidence Interval, p25: 25% percentile, p75: 75% percentile.
196 [1] Nursing staff: nurses and nursing assistants.
197 [2] Medical staff: resident physicians and specialists.
198 [3] Service staff: security, maintenance, cleaning and kitchen.
199 [4] Nurses, nursing assistants, resident physicians and specialists.
200 [5] Middle and superior technicians, security, maintenance, cleaning, kitchen, porter, administrative, and other.
201 [6] Comorbidities: hypertension, obesity (BMI≥30), heart disease, liver disease, diabetes, chronic respiratory disease, renal disease, cancer, autoimmune disorders and other immunological
202 disorders.
203 [7] Chi-squared test for categorical variables (Fisher's exact test corrected for continuity) and median test for continuous variables.
204 [8] Adjusted for sex, age (continuous), ICO centre, type of health care workers, telework and cohabitants.

For peer review only - http://bmjopen.bmj.com/site/about/guidelines.xhtml

Seroprevalence among on-site workers was 8.8% (95%CI: 7.15-10.71) (**Table 2**). Onsite workers were younger, mostly health care workers, and reported more frequently rRT-PCR previous to serology than teleworkers, but no differences were observed in sex, self-reported comorbidities, smoking history, cohabiting with COVID-19 positive case between them and teleworkers (**Supplemental Material**). Among this group (*N*=981) of professionals who never or occasionally teleworked SARS-CoV-2 seropositivity was not associated with not working in a COVID-19 area (aPR: 1.29, 95%CI: 0.81-2.06), nor being in contact with COVID-19 biological samples (aPR: 1.30, 95%CI: 0.77-2.20) nor being in contact with patients with COVID-19 (aPR: 1.09, 95%CI: 0.66-1.79) were associated with SARS-CoV-2 positivity (**Table 2**). On-site nursing or medical staff who worked in a COVID area had twofold SARS-CoV-2 seroprevalence than others who did not work in COVID area (aPR: 2.45, 95%CI: 1.08-5.52). Seropositivity was higher among those whom referred being exposed by interacting with colleagues (aPR: 3.26, 95%CI: 1.49-7.15). On-site workers who self-reported symptoms of COVID-19 were almost 10-fold more likely to be seropositive than those who did not (aPR: 9.5, 95%CI: 5.34-17.03). Most of the on-site workers were highly adherent to the recommendation of hand hygiene at work. Hand washing before eating or working, were followed by more than 97% of on-site workers, whereas around 24% of them reported not hand hygiene after working or a low frequency of handwashing during the workday. In relation to protective measures at work, 17.4% of the on-site workers did not feel protected with PPE, and 12.1% did not use PPE with confirmed or suspicious COVID-19 cases. About colleagues' behaviour, 2m safety distance from colleagues when having lunch was reported to be unfollowed by 14.1% (**Table 2**).

For peer review only - http://bmjopen.bmj.com/site/about/guidelines.xhtml

Table 2. Occupational factors associated with SARS-CoV-2 positive serology among on-site workers (*N*=981).

	Total participants *n* (%)	SARS-CoV-2 seroprevalence *n* (%)	Prevalence (95%CI)	*p-value* [2]	Adjusted PR (95% CI) [3]
**On-site workers**	981 (79.4)	86 (78.1)	8.77 (7.15-10.71)	*0.56*	
**Type of transport to work**					
Private	751 (76.6)	66 (76.7)	8.79 (6.96-11.04)		REF
Public	154 (15.7)	15 (17.4)	9.74 (5.95-15.54)		1.32 (0.74-2.36)
Private and Public	35 (3.6)	2 (2.3)	5.71 (1.43-20.19)		0.63 (0.15-2.58)
Walking	37 (3.8)	3 (3.5)	8.11 (2.63-22.34)	*0.89*	0.57 (0.14-2.35)
**Working in a COVID-19 area**					
No	398 (40.6)	29 (33.7)	7.29 (5.11-10.29)		REF
Yes	545 (55.6)	55 (63.9)	10.09 (7.83-12.92)	*0.14*	1.29 (0.81-2.06)
**Type of and COVID area** [1]					
Non-assisting HCW & never worked in a COVID-19 area	148 (15.1)	7 (8.0)	4.73 (2.27-9.6)		REF
Non-assisting HCW & ever worked in a COVID-19 area	230 (23.4)	13 (15.1)	5.65 (3.31-9.5)		1.12 (0.44-2.82)
Assisting HCW & never worked in a COVID-19 area	244 (24.9)	22 (25.6)	9.02 (6.01-13.32)		1.81 (0.77-4.26)
Assisting HCW & ever worked in a COVID-19 area	311 (31.7)	40 (46.5)	12.86 (9.57-17.07)	*0.006*	2.45 (1.08-5.52)
p-trend					0.26
**Contact with COVID-19 cases**					
No	333 (33.9)	23 (26.7)	6.91 (4.63-10.18)		REF
Yes	536 (54.6)	57 (66.3)	10.63 (8.29-13.54)	*0.07*	1.30 (0.77-2.20)
**Contact with COVID-19 biological samples**					
No	646 (65.9)	51 (59.3)	7.89 (6.05-10.24)		REF
Yes	282 (28.7)	30 (34.9)	10.64 (7.54-14.81)	*0.17*	1.09 (0.66-1.79)
**Reporting to be exposed to COVID-19 by interacting with colleagues at work**					
No	242 (24.7)	66 (76.7)	2.89 (1.38-5.95)		REF
Yes	608 (62.0)	7 (8.1)	10.86 (8.62-13.59)	*<0.0001*	3.26 (1.49-7.15)
**Reporting COVID-19 compatible symptoms**					
No	623 (63.5)	15 (17.4)	2.41 (1.46-3.96)		REF
Yes	306 (31.2)	68 (79.1)	22.22 (17.91-27.23)	*<0.0001*	9.53 (5.34-17.03)

For peer review only - http://bmjopen.bmj.com/site/about/guidelines.xhtml

For peer review only

242

Table 2 (continued)

	Total participants	SARS-CoV-2 seroprevalence	Prevalence (95%CI)	*p-value* [2]	Adjusted PR (95% CI) [3]
**Not following protection measures at work**					
Felt protected with PPE	132 (17.4)	12 (16.9)	9.09 (5.23-15.34)	*0.83*	0.98 (0.51-1.88)
Colleagues cover themselves with their elbows when sneezing/coughing	155 (15.8)	21 (24.4)	13.55 (9.00-19.90)	*0.01*	1.70 (1.01-2.87)
2m safety distance from colleagues during lunch	127 (14.1)	12 (15.6)	9.45 (5.44-15.91)	*0.71*	1.06 (0.56-1.99)
Use of PPE with confirmed or suspicious COVID-19 patients	79 (12.1)	7 (10.45)	8.86 (4.28-17.46)	*0.63*	1.01 (0.45-2.26)
PPE removal safety	48 (7.3)	3 (4.6)	6.25 (2.03-17.68)	*0.33*	0.54 (0.17-1.74)
Personal use of mask	34 (3.5)	1 (1.2)	2.94 (0.41-18.17)	*0.21*	0.41 (0.06-2.99)
Colleagues use of surgical mask	7 (0.7)	1 (1.2)	14.29 (1.96-58.12)	*0.62*	1.68 (0.23-12.29)
**Not following hand hygiene at work**					
≤7 times during workday	233 (23.8)	15 (17.4)	6.44 (3.92-10.41)	*0.13*	0.71 (0.39-1.28)
After money, phone and other personal tools manipulation	175 (17.8)	16 (18.6)	9.14 (5.67-14.41)	*0.89*	1.00 (0.58-1.74)
Every time entering in a new workspace	102 (10.4)	5 (5.8)	4.90 (2.05-11.25)	*0.14*	0.55 (0.22-1.37)
Before working	21 (2.1)	3 (3.5)	14.29 (4.67-36.17)	*0.37*	1.72 (0.54-5.47)
After finishing the workday	17 (1.7)	1 (1.2)	5.88 (0.82-32.09)	*0.67*	0.65 (0.09-4.72)
Before eating	9 (0.9)	2 (2.3)	22.22 (5.59- 57.95)	*0.16*	2.67 (0.65-10.94)

243 Numbers do not always sum up the total due to some missing value (none of the categories present more than 5% of missing values).
244 PR: Prevalence Ratio, CI: Confidence Interval, HCW: Health Care Workers.
245 [1] Assisting HCW: nurses, nursing assistants, resident physicians and specialists; otherwise, classified and non-assisting HCW.
246 [2] Chi-squared test.
247 [3] Adjusted for sex, age (continuous), ICO centre, care staff, telework and cohabitants.

For peer review only - http://bmjopen.bmj.com/site/about/guidelines.xhtml

Concerning the correlates of seropositivity according to household factors for all participants (**Table 3**), seropositivity was associated with living with a COVID-19 positive person (aPR: 3.86, 95%CI: 2.49-5.98). Up to 17.3% of the participants did not take a shower nor change clothes upon home arrival, but the majority (99.0%) did hand hygiene. The least followed hand hygiene home practices were after money, phone and other personal tools manipulation, and after nose blowing, coughing or sneezing (23.5% and 22.7%). However, not following protection measures or hand hygiene at home were associated with a higher SARS-CoV-2 seroprevalence.

Clinical characteristics were collected for those participants (*N*=469) who reported a rRT-PCR performed previous to serology (**Supplemental material**). The majority of the patients with a positive serology and reporting a positive rRT-PCR presented compatible COVID-19 symptoms (74.4%). Among seropositive patients, the most common symptoms were arthromyalgia, cough, headache, asthenia and anosmia. Reporting a positive rRT-PCR when presenting compatible symptoms was associated with a threefold higher prevalence of seropositivity (aPR: 3.10, 95%CI: 1.78-5.31). An increased number of compatible symptoms was also associated with a higher seroprevalence (aPR: 7.4, 95%CI: 1.78-5.31, for presenting four or more symptoms compared to no symptoms).

For peer review only

Table 3. Household factors associated with SARS-CoV-2 positive serology among study participants (n=1,235).

	**Total participants** *n* (%)	**SARS-CoV-2 seroprevalence** *n* (%)	**Prevalence (95%CI)**	***p-value*** [2]	**Adjusted PR (95% CI)** [3]
**Study participants**	1,235	110	8.91 (7.44-10.63)		
**Cohabitants with COVID-19**[1]					
No	894 (79.9)	52 (54.7)	5.82 (4.46-7.56)		REF
Yes	141 (12.60)	34 (35.8)	24.11 (17.76-31.86)	*<0.0001*	3.86 (2.49-5.97)
**Cohabitants cover themselves with their elbow when sneezing**					
No	158 (14.1)	18 (18.9)	11.39 (7.29-17.37)		REF
Yes	919 (82.1)	73 (76.8)	7.94 (6.36-9.88)	*0.15*	0.73 (0.43-1.22)
**Not following protection measures at home**[4]					
Use of face mask when shopping	17 (1.4)	2 (1.8)	11.76 (2.95-36.86)	*0.67*	0.98 (0.24-4.05)
Shower and clothes changing afterwork or upon home arrival	214 (17.3)	20 (18.2)	9.35 (6.11-14.05)	*0.82*	1.02 (0.62-1.69)
**Not following hand hygiene at home**[4]					
Upon arrival	12 (1)	2 (1.8)	16.67 (4.19-47.76)	*0.35*	1.59 (0.39-6.60)
Before eating	60 (4.9)	9 (8.2)	15.00 (7.99-26.4)	*0.09*	1.55 (0.77-3.12)
After money, phone and other personal tools manipulation	290 (23.5)	27 (24.6)	9.31 (6.46-13.24)	*0.71*	1.01 (0.65-1.58)
After cleaning	110 (8.9)	8 (7.3)	7.27 (3.68-13.88)	*0.53*	0.78 (0.38-1.61)
After nose blowing	280 (22.7)	25 (22.7)	8.93 (6.1-12.88)	*0.99*	0.93 (0.58-1.48)

Numbers do not always sum up the total due to some missing values (none of the categories present more than 5% of missing values).
PR: Prevalence Ratio, CI: Confidence Interval.
[1] Analyses performed among those participants who reported having cohabitants (n=1,119).
[2] Chi-squared test.
[3] Adjusted for sex, age (continuous), ICO center, care staff, telework and cohabitants.
[4] Unfollowing the measures of protection and hand hygiene recommendations.

For peer review only - http://bmjopen.bmj.com/site/about/guidelines.xhtml

DISCUSSION

Despite the impact of COVID-19 in oncological patients (10), there are scarce SARS-CoV-2 seroprevalence studies in comprehensive cancer centres with large sample sizes. The global SARS-CoV-2 seroprevalence was 8.9% during the first wave of the COVID-19 pandemic, lower than expected, owing to the presumed higher risk among HCW. Also, it was lower than the reported estimates in two studies performed among HCW in Catalonia between March-April and May 2020, showing a seroprevalence of 11.2% (11) and 10.3% (12), respectively. In all cases, the seroprevalence was higher than in the general population, estimated to be of a maximum of 7.4% in the Barcelona metropolitan area when the study was conducted (13). Seroprevalence studies interpretation must be related to the average COVID-19 prevalence at the time of blood collection. Both of the mentioned studies were carried out earlier than ours, which was performed approximately one month later (21st May-26th June 2020), and two months after the first-wave peak in Catalonia (23th March) (14). Another explanation for this lower seroprevalence in our Centre concerns the participation: all active HCW, regardless of their teleworking status during the previous months or work absenteeism, were invited to participate, and most did (64%). In contrast, García-Basteiro's (11) and Barallat's (12) studies comprised general hospitals (10,11) and primary health care centers (12) in which the incidence could be higher than in a monographic cancer centre.

Several studies regarding COVID-19 infections in HCW in Spain have been published, although showing diverse results. In a tertiary-care hospital in Mallorca, with low regional seroprevalence in the general population (<2%), the prevalence of infected HCW (n=2,210) was 2.8%(15). Varona et al. performed a cross-sectional study evaluating 6,038 employees from the healthcare system of 17 hospitals across four regions in Spain (Madrid, Catalonia, Galicia and Castilla-Leon), showing an 11% seropositivity for SARS-CoV-2 IgG (16). Finally, other studies in Madrid, reported a seroprevalence between 16.6% and 36.5% among HCW in areas with high COVID-19 prevalence (17–19). These studies revealed seroprevalence of SARS-CoV-2 IgG antibodies in HCW tend to be higher than in the general population, at variance according to regional COVID-19 incidence.

The prevalence of SARS-CoV-2 antibodies among HCW has been increasingly investigated in many other countries showing a broad range of outcomes. So far, two systematic reviews estimated an overall seroprevalence of SARS-CoV-2 antibodies of 8.7% and 8.0% among 127,480 HCW and 168,200 HCW, respectively, before vaccination started (2,20).

For peer review only - http://bmjopen.bmj.com/site/about/guidelines.xhtml

Seroprevalence was higher in studies conducted in North America (12.7%) compared with those conducted in Europe (8.5%), Africa (8.2) and Asia (4%) (2).

In Europe, seroprevalence rates among HCW in Germany, Denmark and Belgium were low (1.6%, 4.0% and 6.4%, respectively) (21–23). These studies were conducted during early stages of the epidemic, and therefore, they derived that infection was community-acquired. Also, the Belgian study, with a sample size of almost 30,000 HCW, notes that the high availability of PPE, high standards of infection prevention, and PCR screening in symptomatic staff, coupled with contact tracing and quarantine, might explain the relatively low seroprevalence (23). An study performed in Lombardy, Italy (24), one of the Italian regions most hit by the first epidemic wave, showed a seroprevalence of 7.4% (3.8-11.0%), similar to the observed in the Catalan studies (11,12). Sweden and the UK were the two European countries reporting the highest seropositivity rates among HCW: 19.1% and between 18.0% and 45.3%, respectively (25–27). In the UK, this high seroprevalence was settled in London during the week with the highest number of new cases in the city in the first wave, with around 15% seropositivity among the general population. In the USA, the prevalence of infection among HCW was 10.7%, despite high variation, as low as 1.1% in California (28) to 13.7% in New York State (29).

Despite SARS-CoV-2 seropositivity rate in oncological HCW has significant implications for oncological patients, scant research has been done. The only study published with a large sample size was in Tokyo, Japan, and it showed a very low seroprevalence of 0.67% among 1,190 HCW. It was performed at the end of the first wave in Japan, between the 3rd of August and the 30th of October 2020, so this may explain the lower seroprevalence compared with our estimation. A French study performed among 663 HCW and 1,011 cancer patients, after the end of the first wave, showed also low seroprevalence both for HCW and patients (1.8% and 1.7%, respectively) (30). Other studies that have been published were based on small sample sizes and showed very variable seroprevalence rates (22,31–35).

In our study, we found no differences in HCW seroprevalence according to sex, age and presence of comorbidities. Current or past smoking was however inversely associated to SARS-CoV-2 seroprevalence. Early studies in selected cohorts of COVID-19 patients showed a paradoxical higher risk of SARS-CoV-2 infection among non-smokers (36) whilst ever smokers showed higher risk of COVID-19 progression, including severity of the disease, Intensive Care Unit admission and death (27,28,37).

For peer review only - http://bmjopen.bmj.com/site/about/guidelines.xhtml

It is worth mentioning that, unlike most of the other published seroepidemiological studies among HCW, the present study was performed among all the HCW of the institution, regardless they did full-time telework during the study period (21.6%). No differences by telework were found, and among all study participants the main factor associated with SARS-CoV-2 seropositivity was living with a COVID-19 case, with a times higher probability, similarly to what has been described in other studies (2,20). This finding supports the importance of community dissemination of the infection also for HCWs.

Our study shows that among on-site HCW in an oncological centre, working as medical care staff (nursing, nursing assistant, resident physicians and specialists) in COVID-19 areas stood out as one of the main factors associated with developing SARS-CoV-2 antibodies. Published results regarding the possibility of in-hospital infection among HCW and transmission at work are controversial. Some studies did not find any relation between working in COVID unit or professional category with seropositivity (11,24) whereas other studies reported that seroprevalence was strongly associated with patient related-work (16,22,25,38).

Contact with colleagues at work is potentially a risky situation for transmission among HCW as well as the relaxation of protective measures at the end of the working day. In our study, the on-site HCW who reported being exposed to COVID-19 by other colleagues presented an almost four-fold probability of being seropositive. Most of the HCW declared to follow the protective measures at the workplace, and no differences in seroprevalence were found according to protective measures and hand hygiene.

Protecting HWC health is of paramount importance for reducing morbidity and mortality, reducing transmission, and maintaining the health system capacity (39). Thus international health authorities recommend screening strategies for SARS-CoV-2 infection in exposed or high-risk HCW (40) as well as massive COVID-19 vaccination (41).

Significant differences exist in SARS-CoV-2 testing between countries, and existing programmes focus on screening symptomatic rather than asymptomatic staff. Published studies point out the fact that screening should be performed regardless of the absence of typical symptoms for COVID-19 disease. It has been demonstrated that seroconversion can occur in HCW who have suffered no previous symptoms of SARS-CoV-2 infection (42,43) as asymptomatic transmission is very relevant in SARS-CoV-2 spread (44,45). Thus, the approach for mass testing of both symptomatic and asymptomatic HCW could mitigate

For peer review only - http://bmjopen.bmj.com/site/about/guidelines.xhtml

workforce depletion by unnecessary quarantine, reduce spread in atypical, mild, or asymptomatic cases; and protect patients and health-care workforce.

Among the potential limitations of the study, some recall bias is possible as the data for the correlates of SARS-CoV-2 infection rely on a self-administered questionnaire. Also, response and perception biases must be considered, as well as complacency bias. Results, especially those regarding the accomplishment of preventive measures, might be overestimated. Answers reported in the questionnaire could be influenced by the participants' knowledge regarding their COVID status. However, this study is the first seroepidemiological study with such a large sample size settled in an oncological health centre. The sufficient sample size and high response rate (64.3%) are strengths of the study, although information regarding non-participants was not collected, and we cannot disregard a potential participation bias. However, the distribution by age and sex was similar between participants and non-participants and a possible reason for no participation is that professionals from ICO-Badalona had previously participated in a HCW county seroprevalence survey (12). Also, the fact that the information of the study and the questionnaire was published online and sent by e-mail, as well as the short period of time stablished to respond to it, could have limited the participation. Questionnaire completeness was very high, with no variables presenting more than 5% of missing values.

In conclusion, SARS-CoV-2 seroprevalence among ICO HCW at the end of the first wave of the pandemic was lower than the reported in other Catalan hospitals, but higher than among the general population living in the area. Whereas the main risk factor was living with infected people, among on-site workers, contact with colleagues was associated with SARS-CoV-2 infection. Knowing the seroprevalence rate and follow-up evaluation of persistence may help hospitals to characterize the staff at risk, rationalize their placement, prioritize the use of PPE, thereby potentially reducing the risk of infection. Follow-up studies to evaluate long term durability of antibodies among HCW will be of interest, after the introduction of COVID-19 vaccination among HCW, to better promote infection control in this group. Strengthening preventive measures and health education among HCW is fundamental, especially in oncological departments and centres.

For peer review only - http://bmjopen.bmj.com/site/about/guidelines.xhtml

Contributors EF, DCP, AP, CC, AC and AS contributed to study design. SC, AD, LG, IB, JT, MG, FS, JJT, DC, AS, BC, DR, CG and AP accrued participants and care for blood collection at ICO centres. Laboratory analyses were coordinated by MADL. The questionnaire was designed by DCP and EF, and revised by PPT, ASL, YB, DC, AP, and LA. Questionnaire's implementation was done by EL, JM, JPR, CMM. Data were analysed by YB and DC. PPT, ASL, YB, DC, LA, and EF interpreted the initial results and designed the tables. All authors contributed to interpretation of results. The first draft of the manuscript was prepared by PPT and ASL. PPT, ASL, YB, DC, LA, DC and EF were the main contributors to the writing of the manuscript. All authors assisted in manuscript review. The co-senior authors had full access to all the data in the study for interpretation and had final responsibility for manuscript generation and review, and the decision to submit for publication. EF is the guarantor.

Funding This work was supported by the Catalan Institute of Oncology. PPT is partially supported by the Instituto de Salud Carlos III (ISCIII), Government of the Kingdom of Spain, co-funded by FEDER funds/European Regional Development Fund (ERDF) - a way to build Europe (CM19/00216). The Ministry of Universities and Research, Government of Catalonia partly supports EF and DCP (2017SGR319) and PPT, AS, YBM, DC and LA (2017SGR1085). We thank CERCA Programme / Generalitat de Catalunya for institutional support.

Competing interests JJ received research funding from Kyowa Kirin and from Angelini Pharma for congress attendance. The Cancer Epidemiology Research Programme has received grants from Merck & Co., Roche, GSK, IDT, Hologic, and Seegene.

Acknowledgments The authors acknowledge all the health care workers who participated in the study as well as all the ICO staff involved in the logistics of the different aspects of the study.

Data availability statement No additional data available

For peer review only - http://bmjopen.bmj.com/site/about/guidelines.xhtml

REFERENCES

1. Ng K, Poon BH, Kiat Puar TH, Shan Quah JL, Loh WJ, Wong YJ, et al. COVID-19 and the Risk to Health Care Workers: A Case Report. Annals of internal medicine. 2020 Jun 2;172(11):766–7.
2. Galanis P, Vraka I, Fragkou D, Bilali A, Kaitelidou D. Seroprevalence of SARS-CoV-2 antibodies and associated factors in healthcare workers: a systematic review and meta-analysis. J Hosp Infect. 2021 Feb;108:120–34.
3. Informe nº 56. Situación de COVID-19 en España. Casos diagnosticados a partir de 10 de mayo. Instituto de Salud Carlos III; Red Nacional de Vigilancia Epidemiológica. España; 2020. Available: https://www.isciii.es/QueHacemos/Servicios/VigilanciaSaludPublicaRENAVE/EnfermedadesTransmisibles/Documents/INFORMES/Informes%20COVID-19/Informe%20COVID-19.%20N%c2%ba%2056_09%20de%20diciembre%20de%202020.pdf
4. Centro de Coordinación de Alertas y, Emergencias Sanitarias. Actualización no 517. Enfermedad por el coronavirus (COVID-19). 03.12.2021 [Internet]. [cited 2022 Feb 1]. Available from: https://www.sanidad.gob.es/profesionales/saludPublica/ccayes/alertasActual/nCov/documentos/Actualizacion_517_COVID-19.pdf
5. Dai M, Liu D, Liu M, Zhou F, Li G, Chen Z, et al. Patients with cancer appear more vulnerable to SARS-CoV-2: A multicenter study during the COVID-19 outbreak. Cancer Discovery. 2020 Jun 1;10(6):783.
6. Leung MST, Lin SG, Chow J, Harky A. COVID-19 and Oncology: Service transformation during pandemic. Cancer Medicine. 2020 Oct 18;9(19):7161–71.
7. Institut Català d'Oncologia [Internet]. [cited 2021 Mar 19]. Available from: http://ico.gencat.cat/ca/l_institut/
8. Videla S, Otero A, Martí S, Domínguez MÁ, Fabrellas N, Delgado-Hito MP, et al. Prevalence of SARS-CoV-2 Infection at the University of Barcelona during the Third COVID-19 Pandemic Wave in Spain. Int J Environ Res Public Health. 2021 Jan;18(12):6526.
9. Barros AJD, Hirakata VN. Alternatives for logistic regression in cross-sectional studies: An empirical comparison of models that directly estimate the prevalence ratio. BMC Medical Res Methodol. 2003 Oct 20;3:1–13.
10. Richards M, Anderson M, Carter P, Ebert BL, Mossialos E. The impact of the COVID-19 pandemic on cancer care. Nature Cancer. 2020 Jun;1(6):565–7.
11. Garcia-Basteiro AL, Moncunill G, Tortajada M, Vidal M, Guinovart C, Jiménez A, et al. Seroprevalence of antibodies against SARS-CoV-2 among health care workers in a large Spanish reference hospital. Nature Comm. 2020 Dec 1;11(1):1–9.
12. Barallat J, Fernández-Rivas G, -Sánchez B Q, Martinez-Caceres. Seroprevalence of SARS-CoV-2 IgG Specific Antibodies among Healthcare Workers in the Northern Metropolitan Area of Barcelona, Spain, after the first pandemic wave. PLoS One. 2020 Dec 28;15(12):e0244348.
13. Pollán M, Pérez-Gómez B, Pastor-Barriuso R, Oteo J, Hernán MA, Pérez-Olmeda M, et al. Prevalence of SARS-CoV-2 in Spain (ENE-COVID): a nationwide, population-based seroepidemiological study. The Lancet. 2020 Aug 22;396(10250):535–44.
14. COVID-19 data and indicators. Sistemes d'Informació dels Serveis d'Atenció Primària-SISAP, Institut Català de la Salut, Departament de Salut, Generalitat de Catalunya. [Internet]. 2020 [cited 2021 Mar 19]. Available from: http://eines.portalics/covid/

For peer review only - http://bmjopen.bmj.com/site/about/guidelines.xhtml

15. Rodriguez A, Arrizabalaga-Asenjo M, Fernandez-Baca V, Lainez MP, Al Nakeeb Z, Garcia JD, et al. Seroprevalence of SARS-CoV-2 antibody among healthcare workers in a university hospital in Mallorca, Spain, during the first wave of the COVID-19 pandemic. Int J Infect Dis. 2021 Apr;105:482–6.
16. Varona JF, Madurga R, Peñalver F, Abarca E, Almirall C, Cruz M, et al. Seroprevalence of SARS-CoV-2 antibodies in over 6000 healthcare workers in Spain. Int J Epidemiol. 2021 May 17;50(2):400–9.
17. Galán MI, Velasco M, Casas ML, Goyanes MJ, Rodríguez-Caravaca G, Losa JE, et al. SARS-CoV-2 Seroprevalence Among All Workers in a Teaching Hospital in Spain: Unmasking The Risk [Internet]. medRxiv; 2020 [cited 2022 Feb 2]. p. 2020.05.29.20116731. Available from: https://www.medrxiv.org/content/10.1101/2020.05.29.20116731v1
18. Pérez-García F, Pérez-Zapata A, Arcos N, De la Mata M, Ortiz M, Simón E, et al. Severe acute respiratory coronavirus virus 2 (SARS-CoV-2) infection among hospital workers in a severely affected institution in Madrid, Spain: A surveillance cross-sectional study. Infect Control Hosp Epidemiol. 2021 Jul;42(7):803–9.
19. Cantero M, Millán-Pérez R, Muñez E, Gómez-Lozano N, Anel-Pedroche J, Parra LM, et al. SARS-COV-2 seroprevalence among all healthcare workers in a tertiary hospital in Spain. Infectious Diseases. 2022 Jan 2;54(1):8–15.
20. Kayı İ, Madran B, Keske Ş, Karanfil Ö, Arribas JR, Pshenichnaya N, et al. The seroprevalence of SARS-CoV-2 antibodies among health care workers before the era of vaccination: a systematic review and meta-analysis. Clin Microbiol Infect. 2021 Sep;27(9):1242–9.
21. Korth J, Wilde B, Dolff S, Anastasiou OE, Krawczyk A, Jahn M, et al. SARS-CoV-2-specific antibody detection in healthcare workers in Germany with direct contact to COVID-19 patients. J Clin Virol. 2020 Jul 1;128:104437.
22. Iversen K, Bundgaard H, Hasselbalch RB, Kristensen JH, Nielsen PB, Pries-Heje M, et al. Risk of COVID-19 in health-care workers in Denmark: an observational cohort study. Lancet Infect Dis. 2020;20(12):1401–8.
23. Steensels D, Oris E, Coninx L, Nuyens D, Delforge ML, Vermeersch P, et al. Hospital-Wide SARS-CoV-2 Antibody Screening in 3056 Staff in a Tertiary Center in Belgium. JAMA. 2020;324(2):195–7.
24. Sotgiu G, Barassi A, Miozzo M, Saderi L, Piana A, Orfeo N, et al. SARS-CoV-2 specific serological pattern in healthcare workers of an Italian COVID-19 forefront hospital. BMC Pulmonary Med. 2020;20(1):1–6.
25. Rudberg AS, Havervall S, Månberg A, Jernbom Falk A, Aguilera K, Ng H, et al. SARS-CoV-2 exposure, symptoms and seroprevalence in healthcare workers in Sweden. Nature Comm. 2020 Dec 1;11(1):1–8.
26. Pallett SJC, Rayment M, Patel A, Fitzgerald-Smith SAM, Denny SJ, Charani E, et al. Point-of-care serological assays for delayed SARS-CoV-2 case identification among health-care workers in the UK: a prospective multicentre cohort study. Lancet Resp Med. 2020;8(9):885–94.
27. Houlihan CF, Vora N, Byrne T, Lewer D, Heaney J, Moore DA, et al. SARS-CoV-2 virus and antibodies in front-line Health Care Workers in an acute hospital in London: Preliminary results from a longitudinal study [pre-print]. Available: medhttps://www.medrxiv.org/content/10.1101/2020.06.08.20120584v1
28. Brant-Zawadzki M, Fridman D, Robinson PA, Zahn M, Chau C, German R, et al. SARS-CoV-2 antibody prevalence in health care workers: Preliminary report of a single center study. PLoS ONE. 2020 Nov 1;15.

For peer review only - http://bmjopen.bmj.com/site/about/guidelines.xhtml

29. Moscola J, Sembajwe G, Jarrett M, Farber B, Chang T, McGinn T, et al. Prevalence of SARS-CoV-2 Antibodies in Health Care Personnel in the New York City Area. JAMA 2020;324(9):893–5.
30. Ladoire S, Goussot V, Redersdorff E, Cueff A, Ballot E, Truntzer C, et al. Seroprevalence of SARS-CoV-2 among the staff and patients of a French cancer centre after first lockdown: The canSEROcov study. Eur J Cancer. 2021 May 1;148:359–70.
31. Favara DM, McAdam K, Cooke A, Bordessa-Kelly A, Budriunaite I, Bossingham S, et al. SARS-CoV-2 Infection and Antibody Seroprevalence among UK Healthcare Professionals Working with Cancer Patients during the First Wave of the COVID-19 Pandemic. Clin Oncol. 2021 Oct;33(10):667–75.
32. Epstude J, Harsch IA. Seroprevalence of COVID-19 antibodies in the cleaning and oncological staff of a municipal clinic. GMS Hyg Infect Control. 2020;15:5–9.
33. Fuereder T, Berghoff AS, Heller G, Haslacher H, Perkmann T, Strassl R, et al. SARS-CoV-2 seroprevalence in oncology healthcare professionals and patients with cancer at a tertiary care centre during the COVID-19 pandemic. ESMO Open. 2020 Sep 2;5(5):e000889.
34. Fong D, San Nicolò KO, Alber M, Mitterer M. Evaluating the longitudinal effectiveness of preventive measures against COVID-19 and seroprevalence of IgG antibodies to SARS-CoV-2 in cancer outpatients and healthcare workers. Wien Klin Wochenschr. 2021 Apr;133(7–8):359–63.
35. Marra A, Generali D, Zagami P, Cervoni V, Gandini S, Venturini S, et al. Seroconversion in patients with cancer and oncology health care workers infected by SARS-CoV-2. Ann Oncol. 2021 Jan;32(1):113–9.
36. Simons D, Shahab L, Brown J, Perski O. The association of smoking status with SARS-CoV-2 infection, hospitalisation and mortality from COVID-19: A living rapid evidence review with Bayesian meta-analyses (version 11). Qeios. 2021 Mar 2;
37. Patanavanich R, Glantz SA. Smoking is associated with COVID-19 progression: A meta-analysis. Vol. 22, Nicotine and Tobacco Research. Oxford University Press; 2020. p. 1653–6.
38. Korth J, Wilde B, Dolff S, Anastasiou OE, Krawczyk A, Jahn M, et al. SARS-CoV-2-specific antibody detection in healthcare workers in Germany with direct contact to COVID-19 patients. J Clin Virol. 2020;128:104437.
39. The Lancet. COVID-19: protecting health-care workers. COVID-19 Prioritization of Diagnostic Testing. Lancet. 2020;395: 922.
41. The Importance of COVID-19 Vaccination for Healthcare Personnel. CDC [Internet]. [cited 2021 Mar 19]. Available from: https://www.cdc.gov/coronavirus/2019-ncov/vaccines/recommendations/hcp.html
42. Black JRM, Bailey C, Przewrocka J, Dijkstra KK, Swanton C. COVID-19: the case for health-care worker screening to prevent hospital transmission. Lancet. 2020;395(10234):1418–20.
43. Rivett L, Sridhar S, Sparkes D, Routledge M, Jones NK, Forrest S, et al. Screening of healthcare workers for SARS-CoV-2 highlights the role of asymptomatic carriage in COVID-19 transmission. eLife. 2020;9:1–20.
44. Shields AM, Faustini SE, Perez-Toledo M, Jossi S, Aldera E, Allen JD, et al. SARS-CoV-2 seroprevalence and asymptomatic viral carriage in healthcare workers: a cross-sectional study. Thorax. 2020 Dec;75(12):1089-1094.
45. Bai Y, Yao L, Wei T, Tian F, Jin DY, Chen L, et al. Presumed Asymptomatic Carrier Transmission of COVID-19. JAMA. 2020;323:1406–7.

For peer review only

Figure 1

Participants' flowchart in the seroprevalence survey, Catalan Institute of Oncology. 21st May-26th June 2020; Spain.

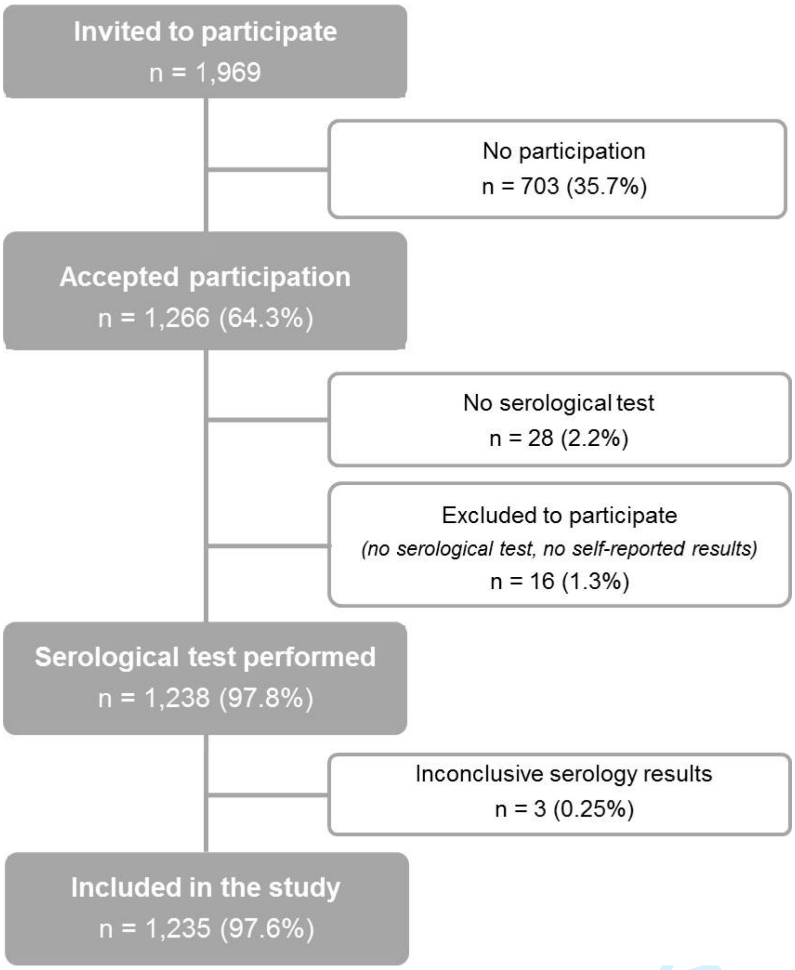

SUPPLEMENTARY MATERIAL

Accompanying the manuscript:

COVID-19 among workers of a Comprehensive Cancer Center between first and second epidemic waves (2020): a seroprevalence study in Catalonia, Spain.

Contents:

- Epidemiologic and behaviour questionnaire – ICO-Sero-COVID Study
- Supplementary Table 1. Demographic characteristics of on-site workers (always/ocassionally) and teleworkers
- Supplementary Table 2. Clinical characteristics associated with SARS-CoV-2 positive serology among those who report rRT-PCR previous to study serology (n=469).

For peer review only

Epidemiologic and behaviour questionnaire – ICO-Sero-COVID Study

I give my consent to participate in the study of seroprevalence of SARS-Cov-2 infection among ICO workers and related companies, which includes responding to an epidemiological survey with information on working conditions and obtaining a nasopharyngeal smear (to perform PCR test for virus detection) and/or to obtain blood sample by venipuncture (to perform serological tests for antibody determination and plasma cryopreservation at ICO biobank)

1 = Yes; 2 = No.

Thank you for participating in the COVID-19 seroprevalence survey among ICO workers. All information provided below will be treated confidentially, and all resulting results will be anonymized, with no individual data identifying participants.

A. Socio-demographic data.

1. Name string variable.
2. Last name1 string variable.
3. Last name2 string variable.
4. CIP string variable.
5. DNI numeric variable.
6. Sex numeric variable (1 = Woman; 2 = Man).
7. E-mail string variable.
8. ICO center or external company cathegoric variable (ICO-Gi, ICO-L'H, ICO-BDN, ICO-Tarragona-Terres Ebre, Arcasa, IDIBELL, ISS, Security, IDI, Pregecsa, Veolia).
9. Professional category numeric variable (1 = Nurse; 2 = Fac. Specialist (medicine, pharmacy, physics, psychologist); 3 = Higher Technician (Research, Predoc, Postdoc ...); 4 = MIR, FIR, PIR; 5 = Higher Technician; 6 = Porter; 7 = Administrative; 8 = Maintenance/Security; 9 = Cleaning; 10 = Restoration; 11 = Other (specify: string variable ___________________).
10. Work shift numeric variable (1 = Morning; 2 = Afternoon; 3 = Night; 4 = Other).
11. Did you telework for at least more than one day during the March to May 2020 period? numeric variable (1 = Yes; 2 = No).
12. How many days on average per week do you telework? | _ _ | numeric variable (1 to 7).

B. Exposure and occupational safety measures data.

13. **Have you worked in the "COVID area" during the period comprised between March and May 2020?** numeric variable (1 = No; 2 = Yes).
14. **Since the beginning of March 2020, have you had a suspected or confirmed clinical condition as COVID-19?** numeric variable (1 = Yes; 2 = No).
15. **Since the beginning of March 2020, have you had a nasopharyngeal smears sample?** numeric variable (1 = Yes; 2 = No).
16. **Do you belong to any of the groups considered to have an increased vulnerability to COVID -19?**
    a. **Cardiopathy / Hypertension** numeric variable (1 = Yes; 2 = No).
    b. **Respiratory disease** numeric variable (1 = Yes; 2 = No).
    c. **Hepatopathy** numeric variable (1 = Yes; 2 = No).
    d. **Nephropathy** numeric variable (1 = Yes; 2 = No).
    e. **Active Cancer** numeric variable (1 = Yes; 2 = No).
    f. **Immunosuppression** numeric variable (1 = Yes; 2 = No).
    g. **Diabetes mellitus** numeric variable (1 = Yes; 2 = No).
    h. **Pregnancy** numeric variable (1 = Yes; 2 = No).
17. **Have you had contact with patients with COVID-19 infection at ICO?** numeric variable (1 = Yes; 2 = No).
18. **Have you had contact with samples of COVID-19 patients at ICO?** numeric variable (1 = Yes; 2 = No).

For peer review only - http://bmjopen.bmj.com/site/about/guidelines.xhtml

19. **When you are in your workplace, do you wear a surgical mask?** numeric variable (1 = Yes; 2 = No).
20. **If you are in the COVID-19area, do you wear Personal Protective Equipment (PPE)?** numeric variable (1 = Yes; 2 = No, 3 = Not Applicable).
21. **Do you think that the Personal Protective Equipment (PPE) removal procedure is safe?** numeric variable (1 = Yes; 2 = No, 3 = I don't know, 4 = Not Applicable).
22. **Do you feel protected by the Personal Protective Equipment (PPE) used?** numeric variable (1 = Yes; 2 = No, 3 = I don't know, 4 = Not Applicable).
23. **Do you think that you may have been exposed to COVID-19 during personal relationships with your co-workers?** numeric variable (1 = Yes; 2 = No, 3 = I don't know).
24. **Do you think that the protection procedures implemented during this pandemic period will benefit you in your future professional development?** numeric variable (1 = Yes; 2 = No, 3 = I don't know).
25. **Do you think that the work activity carried out during this pandemic period has affected you or will affect you emotionally in the future?** numeric variable (1 = Yes; 2 = No, 3 = I don't know).

At work, do you wash your hands with soap or water or with a hydro-alcoholic solution...

26. **... before you start working?** numeric variable (1 = Yes; 2 = No).
27. **... every time you enter a new workspace?** numeric variable (1 = Yes; 2 = No).
28. **... before eating?** numeric variable (1 = Yes; 2 = No).
29. **... after handling money, mobile phone, other utensils ...?** numeric variable (1 = Yes; 2 = No).
30. **... less than 7 times during the working day?** numeric variable (1 = Yes; 2 = No).
31. **... at the end of the working day?** numeric variable (1 = Yes; 2 = No).

32. **When you eat, do you maintain a distance ≥ 2m from your colleagues?** numeric variable (1 = Yes; 2 = No, 3 = Not Applicable).
33. **Did your collegues cover thir face with their elbows when they sneeze / cough?** numeric variable (1 = Yes; 2 = No).

C. COVID-19 exposure outside working environament (home and social activities).

Outside working environment, do you wash your hands (with soap and water or hydro-alcoholic solution)...

34. **... when you get home?** numeric variable (1 = Yes; 2 = No).
35. **... before eating?** numeric variable (1 = Yes; 2 = No).
36. **... after handling money, mobile phone, other utensils** numeric variable (1 = Yes; 2 = No).
37. **... after cleaning?** numeric variable (1 = Yes; 2 = No).
38. **... after blowing your nose, sneezing or coughing?** numeric variable (1 = Yes; 2 = No).
39. **Do you shower and change clothes when you get home (or did you go to work)?:** numeric variable (1 = Yes; 2 = No).
40. **Do you wear a mask when you go shopping?** numeric variable (1 = Yes; 2 = No)
41. **Do the people you live with cover their elbows if they sneeze / cough?** numeric variable (1 = Yes; 2 = No)
42. **Do you have a cohabitant who has passed COVID-19 (with symptoms, with or without confirmation by PCR, or PCR + without symptoms)?** numeric variable (1 = Yes; 2 = No).
43. **Do you use public transport to go to work?** numeric variable (1 = Yes; 2 = No)
    If "yes", continue with question 44; if "no , jump to question 46.
44. **Which type of public transport?** numeric variable (1 = bus, 2 = metro, 3 = train, 4 = taxi, 5 = bicycle (multiple answer allowed)).
45. **How many days a week do you use public transport?** | _ _ | numeric variable (1 to 7).
46. **Do you use private transportation to get to work?** numeric variable (1 = Yes; 2 = No).
    If "yes", continue with question 47; if "no", jump to question 49.
47. **Which private transport?** numeric variable (1 = single use car, 2 = shared car, 3 = single use bike, 4 = shared bike, 5 = bike (multiple answer allowed)).
48. **How many days per week do you use private transport?** | _ _ | numeric variable (1 to 7).

For peer review only - http://bmjopen.bmj.com/site/about/guidelines.xhtml

49. **Do you walk the street for more than 15 minutes?** numeric variable (1 = Yes; 2 = No).
If "yes", continue with question 50; if "no", jump to question 52.
50. **How many days a week do you go for a walk?** | _ _ | numeric variable (1 to 7).
51. **For how many minutes a day do you go for a walk as an average:** | _ _ | minutes / day numeric variable.

D. COVID-19 tests performed

52. **Have you had a COVID-19 PCR test?** numeric variable (1 = Yes, only one; 2 = Yes, several; 3 = No).
If "1", continue with question 53; if "2", jump to question 55; if "3", jump to question 59.
53. **COVID-19 PCR test performed on day:** | *dd* | *mm* | *yy*|.
54. **COVID-19 PCR test result** numeric variable (1 = Negative; 2 = Positive).
55. **How many COVID-19 PCR test have you had in total?** | _ _ | numeric variable (1 to 10).
Depending on the answer, open as many questions with the number of PCR made to ask the date and result in the same format (A1 and A2; B1 and B2; etc).
A1. **COVID-19 PCR test performed on day:** | *dd* | *mm* | *yy*|.
A2. **COVID-19 PCR test result** numeric variable (1 = Negative; 2 = Positive).
56. **When you had your first COVID-19 PCR test, did you present any of these signs or symptoms?** numeric variable (multiple answer allowed) (1 = Febricula (>37.3ºC); 2 = Fever (>38°C); 3 = Cough; 4 = Odynophagia (sorethroat); 5 = Headache; 6 = Arthromyalgia (generalized pain); 7 = Asthenia (intense fatigue); 8 = Dyspnoea (shortness of breath); 9 = Anosmia (loss of smell); 10= Nausea, vomiting; 11 = Diarrhea; 12 = Skin lesions; 13 = Myoclonus (involuntary movements); 14 = Pneumonia; 15 = Other (specify: string variable __________________).
57. **Have you had a COVID-19 rapid antibody test?** numeric variable (1 = Yes; 2 = No).
58. **COVID-19 rapid antibody test result** numeric variable (1 = Negative; 2 = Positive).

Finally, we would like to complete the information provided with information about your lifestyle.

E. Lifestyle

59. **Do you drink any alcoholic beverage at least once a week?** numeric variable (1 = Yes; 2 = No).
If "yes", continue with question 60; if "no", jump to question 64.
60. **How many glasses of wine do you drink every week?** | _ _ | numeric variable
61. **How many beers do you drink every week?** | _ _ | numeric variable
62. **How many glasses of cognac, gin or other spirits do you drink every week?** | _ _ | numeric variable
63. **Has your alcohol consumption changed during the pandemic compared to your consumption previously?** numeric variable (1 = No, it is similar; 2 = Yes, it has increased; 3 = Yes, it has decreased).
64. **Regarding tobacco use:** numeric variable (1 = I have never smoked; 2 = I am a former smoker; 3 = I am a corrent smoker).
If "1" or "2", jump to question 67; If "3", continue to question 65.
65. **How many roll-ypur-own cigarrettes do you smoke every day?** | _ _ | numeric variable
66. **Has your tobacco consumption changed during the pandemic compared to your consumption previously?** numeric variable (1 = No, it is similar; 2 = Yes, it has increased; 3 = Yes, it has decreased).

F. End of the survey

Thank you very much for your participation. As mentioned before, all information from this survey and the tests performed is confidential and will be anonymized.

If you would like to leave us any further comments regarding the pandemic at the ICO Centers, please do so below:

67. **Commentaries**. *Open answer, leave space for about 5 lines of text.*

For peer review only - http://bmjopen.bmj.com/site/about/guidelines.xhtml

Supplementary Table 1. Demographic characteristics of on-site workers (always/ocassionally) and teleworkers

	Teleworking		
	**Never/ocassionally (n=981)** n (%)	**Always (n=230)** n (%)	***p-value***
**Sex**			
Male	240 (25)	47 (20)	
Female	736 (75)	183 (80)	*0,183*
**Age [median, (min-max)]**	43 (19-68.5)	44.9 (19-71.6)	*0,015*
<35y	271 (28)	38 (17)	
35-49y	429 (44)	122 (53)	
>49y	281 (29)	70 (30)	*0,002*
**ICO Center**			
ICO L'Hospitalet	684 (70)	184 (80)	
ICO Girona	182 (19)	17 (7)	
ICO Badalona	103 (11)	29 (13)	
ICO Tarragona / Terres de l'Ebre	12 (1)	0 (0)	*<0.0001*
**Health care workers**			
Yes	567 (59)	72 (32)	
No	402 (41)	152 (68)	*<0.0001*
Middle and superior technicians	*187 (19)*	*92 (41)*	
Porters	*17 (2)*	*3 (1)*	
Administrative staff	*90 (9)*	*35 (16)*	
Maintenance or security stafft	*29 (3)*	*2 (1)*	
Cleaning staff	*46 (5)*	*15 (7)*	
Restoration staff	*16 (2)*	*2 (1)*	
Others	*17 (2)*	*3 (1)*	*<0.0001*
**Any Comorbidity**	142 (15)	38 (17)	*0,4*
**Smoking history**			
Never	511 (54)	126 (56)	
Ever	438 (46)	98 (44)	*0,5*
Past	277 (29)	66 (29)	
Current	161 (17)	32 (14)	*0,6*
**Cohabiting**	889 (91)	209 (92)	*0,8*
**Cohabiting with covid-19**	115 (14)	27 (14)	*0,9*
**Reported rRT-PCR previous to serology**	422 (84)	42 (75)	*0,1*
**Positive of previous rRT-PCR**	62 (15)	10 (24)	*0,1*

r review only

Supplementary Table 2. Clinical characteristics associated with SARS-CoV-2 positive serology among those who report rRT-PCR previous to study serology (n=469).

	Total participants n (%)	SARS-CoV-2 seroprevalence n (%)	Prevalence (95%CI)	p-value[1]	Adjusted PR (95% CI)[2]
**Reported rRT-PCR previous to serology**	469 (38.0)	86 (78.2)	18.34 (15.08-22.11)		
**Result of previous rRT-PCR**					
Negative	397 (84.6)	27 (31.0)	6.80 (4.70-9.74)		REF
Positive	72 (15.4)	59 (68.6)	81.94 (71.31-89.23)	<0.001	12.15 (7.54-19.57)
**Number of symptoms***(mean, standard deviation)*	1.65 (2.10)	3.08 (2.61)		<0.001	
None	217 (46.3)	21 (24.0)	9.68 (6.39-14.4)		REF
One	61 (13)	7 (8.1)	11.48 (5.56-22.21)		1.13 (0.48-2.67)
2-3	109 (23.2)	22 (25.6)	20.18 (13.66-28.78)		2.03 (1.10-3.73)
≥4	81 (17.3)	35 (40.7)	43.21 (32.87-54.18)	<0.001	4.33 (2.48-7.59)
p-trend (among exposed)					*<0.001*
**Reporting COVID-19 compatible symptoms when rRT-PCR was performed**					
No	217 (46.3)	21 (24.0)	9.68 (6.39-14.4)		REF
Yes	251 (53.5)	64 (74.4)	25.5 (20.48-31.27)	<0.001	2.49 (1.51-4.10)
**COVID-19 symptoms**					
Headache	126 (26.9)	36 (41.9)	28.57 (21.35-37.08)	<0.001	1.87 (1.20-2.93)
Cough	119 (25.4)	37 (43.0)	31.09 (23.42-39.97)	<0.001	2.25 (1.44-3.52)
Asthenia	110 (23.5)	36 (41.9)	32.73 (24.6-42.04)	<0.001	2.38 (1.53-3.72)
Arthromyalgia	80 (17.1)	57 (66.0)	36.25 (26.47-47.31)	<0.001	2.32 (1.47-3.67)
Low-grade fever (37.3ºC-38ºC)	73 (15.6)	26 (30.2)	35.62 (25.5-47.21)	<0.001	2.71 (1.67-4.39)
Odynophagia	64 (13.6)	14 (16.3)	21.88 (13.39-33.65)	0.40	1.18 (0.65-2.13)
Diarrhoea	58 (12.4)	16 (18.6)	27.59 (17.62-40.43)	0.05	1.47 (0.83-2.60)
Anosmia	42 (9)	33 (38.4)	78.57 (63.65-88.48)	<0.001	6.09 (3.86-9.60)
Dyspnoea	40 (8.5)	11 (12.8)	27.50 (15.91-43.2)	0.12	1.56 (0.81-3.00)
Fever (>38ºC)	28 (6)	15 (17.4)	53.57 (35.4-70.84)	<0.001	3.06 (1.71-5.46)
Nausea / vomiting	17 (3.6)	6 (7)	35.29 (16.75-59.66)	0.07	1.86 (0.80-4.36)
Skin lesions	8 (1.7)	1 (1.2)	12.50 (1.72-53.86)	0.66	0.74 (0.10-5.38)
Pneumonia	3 (0.6)	2 (2.3)	66.67 (15.27-95.69)	0.03	2.99 (0.71-12.63)
Myoclonus	2 (0.4)	0		0.50	

Numbers do not always sum up the total due to some missing values (none of the categories present more than 5% of missing values). PR: Prevalence Ratio, CI: Confidence Interval. [1] Chi-squared test for categorical variables (Fisher's exact test corrected for continuity) and median test for continuous variables. [2] Adjusted for sex, age (continuous), ICO center, care staff, telework and cohabitants.

For peer review only

STROBE 2007 (v4) Statement—Checklist of items that should be included in reports of *cross-sectional studies*

Section/Topic	Item #	Recommendation	Reported on page #
**Title and abstract**	1	(*a*) Indicate the study's design with a commonly used term in the title or the abstract	3
		(*b*) Provide in the abstract an informative and balanced summary of what was done and what was found	3
**Introduction**			
Background/rationale	2	Explain the scientific background and rationale for the investigation being reported	5
Objectives	3	State specific objectives, including any prespecified hypotheses	5
**Methods**			
Study design	4	Present key elements of study design early in the paper	5
Setting	5	Describe the setting, locations, and relevant dates, including periods of recruitment, exposure, follow-up, and data collection	5
Participants	6	(*a*) Give the eligibility criteria, and the sources and methods of selection of participants	5
Variables	7	Clearly define all outcomes, exposures, predictors, potential confounders, and effect modifiers. Give diagnostic criteria, if applicable	7
Data sources/ measurement	8*	For each variable of interest, give sources of data and details of methods of assessment (measurement). Describe comparability of assessment methods if there is more than one group	7
Bias	9	Describe any efforts to address potential sources of bias	5,6,19
Study size	10	Explain how the study size was arrived at	5
Quantitative variables	11	Explain how quantitative variables were handled in the analyses. If applicable, describe which groupings were chosen and why	7
Statistical methods	12	(*a*) Describe all statistical methods, including those used to control for confounding	8
		(*b*) Describe any methods used to examine subgroups and interactions	8
		(*c*) Explain how missing data were addressed	8
		(*d*) If applicable, describe analytical methods taking account of sampling strategy	NA
		(*e*) Describe any sensitivity analyses	NA
**Results**			

For peer review only

Participants	13*	(a) Report numbers of individuals at each stage of study—eg numbers potentially eligible, examined for eligibility, confirmed eligible, included in the study, completing follow-up, and analysed	9
		(b) Give reasons for non-participation at each stage	9
		(c) Consider use of a flow diagram	6
Descriptive data	14*	(a) Give characteristics of study participants (eg demographic, clinical, social) and information on exposures and potential confounders	9
		(b) Indicate number of participants with missing data for each variable of interest	9
Outcome data	15*	Report numbers of outcome events or summary measures	12
Main results	16	(*a*) Give unadjusted estimates and, if applicable, confounder-adjusted estimates and their precision (eg, 95% confidence interval). Make clear which confounders were adjusted for and why they were included	12
		(*b*) Report category boundaries when continuous variables were categorized	9
		(*c*) If relevant, consider translating estimates of relative risk into absolute risk for a meaningful time period	
Other analyses	17	Report other analyses done—eg analyses of subgroups and interactions, and sensitivity analyses	15
**Discussion**			
Key results	18	Summarise key results with reference to study objectives	17
Limitations	19	Discuss limitations of the study, taking into account sources of potential bias or imprecision. Discuss both direction and magnitude of any potential bias	21
Interpretation	20	Give a cautious overall interpretation of results considering objectives, limitations, multiplicity of analyses, results from similar studies, and other relevant evidence	17
Generalisability	21	Discuss the generalisability (external validity) of the study results	21
**Other information**			
Funding	22	Give the source of funding and the role of the funders for the present study and, if applicable, for the original study on which the present article is based	22

*Give information separately for cases and controls in case-control studies and, if applicable, for exposed and unexposed groups in cohort and cross-sectional studies.

Note: An Explanation and Elaboration article discusses each checklist item and gives methodological background and published examples of transparent reporting. The STROBE checklist is best used in conjunction with this article (freely available on the Web sites of PLoS Medicine at http://www.plosmedicine.org/, Annals of Internal Medicine at http://www.annals.org/, and Epidemiology at http://www.epidem.com/). Information on the STROBE Initiative is available at www.strobe-statement.org.

For peer review only
